# Nivolumab versus placebo in patients with relapsed malignant mesothelioma (CONFIRM): a multicentre, double-blind, randomised, phase 3 trial

**DOI:** 10.1016/S1470-2045(21)00471-X

**Published:** 2021-11

**Authors:** Dean A Fennell, Sean Ewings, Christian Ottensmeier, Raffaele Califano, Gerard G Hanna, Kayleigh Hill, Sarah Danson, Nicola Steele, Mavis Nye, Lucy Johnson, Joanne Lord, Calley Middleton, Peter Szlosarek, Sam Chan, Aarti Gaba, Liz Darlison, Peter Wells-Jordan, Cathy Richards, Charlotte Poile, Jason F Lester, Gareth Griffiths, CONFIRM trial investigators, Gillian Price, Paul Shaw, Judith Cave, Jay Naik, Amy Ford, Tom Geldhart, Gairin Dancey, Dionysis Papadatos, Andy Polychronis, Petra Jankowska, Angela Scott, Jill Gardiner, Mathilda Cominos, Lynn Campbell, Carol MacGregor, Lois Mullholand, Meenali Chitnis, Gary Dougherty

**Affiliations:** a Mesothelioma Research Programme, Leicester Cancer Research Centre, University of Leicester, Leicester, UK; b University Hospitals of Leicester NHS Trust, Leicester, UK; c Cancer Research UK, Southampton Clinical Trials Unit, University of Southampton, Southampton, UK; d Mavis Nye Foundation, University of Southampton, Southampton, UK; e Southampton Health Technology Assessments Centre, University of Southampton, Southampton, UK; f Department of Molecular & Clinical Cancer Medicine, University of Liverpool, Liverpool, UK; g Department of Medical Oncology, Wythenshaw Hospital, Manchester, UK; h Peter MacCullum Cancer Centre, University of Melbourne, Melbourne, VIC, Australia; i Department of Radiation Oncology, University of Sheffield, Sheffield, UK; j Department of Oncology and Metabolism University of Glasgow, Glasgow, UK; k Cancer Research UK Barts Cancer Institute, Queen Mary University of London, London, UK; l York Teaching Hospital NHS Foundation Trust, York, UK; m Department of Oncology, Mesothelioma UK, Leicester, UK; n The Rutherford Cancer Centre, Newport, UK

## Abstract

**Background:**

No phase 3 trial has yet shown improved survival for patients with pleural or peritoneal malignant mesothelioma who have progressed following platinum-based chemotherapy. The aim of this study was to assess the efficacy and safety of nivolumab, an anti-PD-1 antibody, in these patients.

**Methods:**

This was a multicentre, placebo-controlled, double-blind, parallel group, randomised, phase 3 trial done in 24 hospitals in the UK. Adult patients (aged ≥18 years) with an Eastern Cooperative Oncology Group performance status of 0 or 1, with histologically confirmed pleural or peritoneal mesothelioma, who had received previous first-line platinum-based chemotherapy and had radiological evidence of disease progression, were randomly assigned (2:1) to receive nivolumab at a flat dose of 240 mg every 2 weeks over 30 min intravenously or placebo until disease progression or a maximum of 12 months. The randomisation sequence was generated within an interactive web response system (Alea); patients were stratified according to epithelioid versus non-epithelioid histology and were assigned in random block sizes of 3 and 6. Participants and treating clinicians were masked to group allocation. The co-primary endpoints were investigator-assessed progression-free survival and overall survival, analysed according to the treatment policy estimand (an equivalent of the intention-to-treat principle). All patients who were randomly assigned were included in the safety population, reported according to group allocation. This trial is registered with Clinicaltrials.gov, NCT03063450.

**Findings:**

Between May 10, 2017, and March 30, 2020, 332 patients were recruited, of whom 221 (67%) were randomly assigned to the nivolumab group and 111 (33%) were assigned to the placebo group). Median follow-up was 11·6 months (IQR 7·2–16·8). Median progression-free survival was 3·0 months (95% CI 2·8–4·1) in the nivolumab group versus 1·8 months (1·4–2·6) in the placebo group (adjusted hazard ratio [HR] 0·67 [95% CI 0·53–0·85; p=0·0012). Median overall survival was 10·2 months (95% CI 8·5–12·1) in the nivolumab group versus 6·9 months (5·0–8·0) in the placebo group (adjusted HR 0·69 [95% CI 0·52–0·91]; p=0·0090). The most frequently reported grade 3 or worse treatment-related adverse events were diarrhoea (six [3%] of 221 in the nivolumab group *vs* two [2%] of 111 in the placebo group) and infusion-related reaction (six [3%] *vs* none). Serious adverse events occurred in 90 (41%) patients in the nivolumab group and 49 (44%) patients in the placebo group. There were no treatment-related deaths in either group.

**Interpretation:**

Nivolumab represents a treatment that might be beneficial to patients with malignant mesothelioma who have progressed on first-line therapy.

**Funding:**

Stand up to Cancer–Cancer Research UK and Bristol Myers Squibb.

## Introduction

Malignant mesothelioma is a universally lethal cancer that is usually caused by exposure to asbestos fibres. It commonly arises in the thoracic parietal pleura or, less frequently, in the abdominal peritoneal lining, in the sac surrounding the heart, and the testes. Mesothelioma comprises three principal histological subtypes that are associated with decreasing survival: epithelioid, biphasic, and sarcomatoid. A pressing unmet need remains for new treatments in the relapsed mesothelioma setting.^1^ Following the approval of pemetrexed and cisplatin for the treatment of pleural mesothelioma in 2004,^2^ no randomised phase 3 trial assessing any novel drug or combination of drugs has yet shown an improvement in overall survival^3^, ^4^ in patients with malignant mesothelioma following disease progression.

Mesotheliomas express PD-L1 in a minority of patients.^5^, ^6^ Promising clinical activity of nivolumab, a fully humanised, IgG4, PD-1-immune checkpoint inhibitor antibody, has been reported in phase 2 trials of patients with malignant pleural mesothelioma.^7^, ^8^, ^9^ In the MERIT trial,^7^ nivolumab was associated with a median overall survival of 17·3 months (95% CI 11·5–not reached), median progression-free survival of 6·1 months (95% CI 2·9–9·9), and a response rate of 26% in patients with advanced or metastatic malignant pleural mesothelioma, leading to its approval in Japan. In the CheckpOiNt Blockade For the Inhibition of Relapsed Mesothelioma (CONFIRM) trial we aimed to evaluate the efficacy of nivolumab on overall survival and progression-free survival in patients with malignant mesothelioma whose disease had progressed following at least one course of platinum-based chemotherapy.

**Research in context**

**Evidence before this study**

We searched MEDLINE from Jan 1, 2009, to Dec 31, 2020, for clinical trials using the terms “mesothelioma”, “relapsed”, or “pleural”, and “peritoneal”, “phase III”, “programmed death-1 or PD-1”, “placebo”, “nivolumab”, without any language restrictions. This search revealed no evidence of any published double-blind, phase 3, trial of an anti-PD-1 checkpoint inhibitor in patients with relapsed mesothelioma. Previous single-group phase 2A studies of nivolumab and other anti-PD-1 inhibitors have shown some clinical activity in this setting (level 2b evidence). However, no phase 3 trial has evaluated an anti-PD-1 immune checkpoint inhibitor as monotherapy versus placebo (ie, level 1A evidence). No randomised phase 3 trials have enrolled patients with peritoneal mesothelioma. PD-L1 has been shown to be a useful predictive biomarker for stratifying therapy with an anti-PD-1 inhibitor (eg, in non-small-cell lung cancer). No study has yet done a double-blind analysis of PD-L1 to determine its prognostic or predictive value for mesothelioma in a placebo-controlled, randomised, phase 3 trial in this setting.

**Added value of this study**

To our knowledge, the CONIFRM trial is the first randomised, phase 3 study to show improved overall survival in patients with relapsed malignant pleural and peritoneal mesothelioma. CONFIRM also met its co-primary endpoint of progression-free survival, with an acceptable level of safety and tolerability. PD-L1 was not found to be predictive or prognostic in the CONFIRM trial, highlighting the need for other predictors of efficacy for this drug class in mesothelioma. CONFIRM builds on three phase 2 clinical trials in the relapsed setting that showed promising single agent efficacy.

**Implications of all the available evidence**

The absence of an international standard of care or evidence for improved survival conferred by any drug in relapsed mesothelioma underpinned the design of the CONFIRM trial, with the goal of determining the specific efficacy of the anti-PD1 inhibitor nivolumab versus placebo in patients with either pleural or peritoneal mesothelioma of any histology. This study confirms nivolumab as an effective treatment that could be a new option in patients with relapsed mesothelioma. Efforts to identify predictive biomarkers of nivolumab are warranted.

## Methods

### Study design and participants

This multicentre, double-blind, placebo-controlled, parallel group, randomised phase 3 trial was designed by the lead authors in collaboration with the sponsor (University of Southampton, Southampton, UK) and done in 24 hospitals in the UK (appendix p 50). Patients (aged ≥18 years) with histologically confirmed pleural of peritoneal mesothelioma of any subtype, who had received at least one course of platinum-based chemotherapy and had subsequently had radiological evidence of disease progression, an Eastern Cooperative Oncology Group (ECOG) performance status score of 0 or 1, measurable disease according to modified Response Evaluation Criteria in Solid Tumors (RECIST) or RECIST version 1.1, and archival tumour biopsy for biomarker analyses were eligible for enrolment into the CONFIRM trial. Patients also had to meet the following laboratory criteria: white blood cell count of at least 2 × 10^9^ cells per L, neutrophil count at least 1·5 × 10^9^ cells per L, platelet count at least 100 × 10^9^ per L, haemoglobin concentration at least 90 g/L, serum creatinine concentration of up to 1·5 × the upper limit of normal (ULN) or creatinine clearance higher than 50 mL/min (using the Cockcroft-Gault formula), aspartate aminotransferase concentration up to 3 × ULN or alanine aminotransferase concentration up to 3 × ULN (if both are assessed, both need to be up to 3 × ULN), and total bilirubin concentration up to 1·5 × ULN (except patients with Gilbert syndrome, who had to have total bilirubin <5·13 μmol/L). Patients were approached in the hospital setting by research staff. There was no restriction on the number of previous therapies received. Key exclusion criteria included previous treatment with an immune checkpoint inhibitor, uncontrolled metastasis involving the CNS, and autoimmune disease. The complete eligibility criteria are provided in the study protocol (appendix). Median survival with no additional treatment was expected to be approximately 6 months for eligible patients.^3^

The study protocol was approved by the West Midlands, Edgbaston Research Ethics Committee (16/WM/0472; appendix p 67). The study was done in accordance with the provisions of the Declaration of Helsinki and Good Clinical Practice guidelines as defined by the International Conference on Harmonisation. Written informed consent was obtained from all patients before enrolment.

### Randomisation and masking

Participants were randomly assigned (2:1) to receive either nivolumab (nivolumab group) or placebo (placebo group). Randomisation was done after baseline measures were recorded and successful screening with an interactive web response system. The randomisation sequence was generated with Alea. Patients were stratified according to epithelioid versus non-epithelioid histology, with random block sizes of 3 and 6. Treating clinicians and participants were masked to group allocation, but unmasking could be requested by the treating clinician following disease progression.

### Procedures

A flat dose of 240 mg of nivolumab or placebo was administered intravenously over 30 min every 2 weeks. Treatment was continued until disease progression, withdrawal from treatment, or for a maximum of 12 months, whichever occurred first. The key reason for the 12-month cap was based on the expected magnitude of both progression-free survival (median 3 months) and overall survival (median 6 months) durations, which are short for patients with mesothelioma in the relapsed treatment setting, and agreed as a pragmatic cap with Bristol Myers Squibb (the supplier of nivolumab). Dose delays were permitted for up to 4 weeks from the previous dose. Criteria for dose delay included any grade 2 non-skin, drug-related adverse events; any grade 3 skin drug-related adverse events; and any grade 3 drug-related laboratory abnormality adverse events. Treatment interruptions were permitted; restarting infusion was recommended for grade 2 symptoms, but discontinuation was recommended for grade 3 adverse events or worse. Full requirements for treatment delay or discontinuation because of treatment-related adverse events are specified in the protocol. Reduction in the treatment dose was not permitted.

Participants were assessed with CT scans on day 1 of each 2-week cycle and 4 weeks after treatment discontinuation. CT scans were not centrally reviewed. Adverse events were assessed at day 1 of each cycle, 4 weeks after discontinuation, and then at 100 days, and up to 100 days after treatment discontinuation, and graded using the National Cancer Institute Common Terminology Criteria for Adverse Events version 4.03. Laboratory parameters (serum chemistry, full blood count, liver function tests, and thyroid function tests) were assessed on day 1 of each cycle until disease progression and 4 weeks after treatment discontinuation.

PD-L1 protein expression was evaluated retrospectively in pretreatment tumour-biopsy specimens with a validated automated immunohistochemical assay that used a rabbit monoclonal antihuman PD-L1 antibody (clone 22-C3) according to guidelines laid out in accordance with PD-L1 immunohistochemistry 22C3 pharmDx (Agilent, Santa Clara, CA, USA). Evaluation was independently validated by a consultant histopathologist (CR) and advanced biomedical scientist (PWJ). Samples were categorised as positive for PD-L1 when the staining of the tumour cell membrane (at any intensity) was observed at a prespecified expression threshold of 1% of cells in a section that included at least 100 evaluable tumour cells.

### Outcomes

The co-primary endpoints were progression-free survival (time from randomisation to disease progression according to masked investigator assessment or death, whichever occurred first) and overall survival (time from randomisation to death from any cause). The co-primary endpoints were monitored every 3 months following discontinuation of treatment. Secondary endpoints were overall response to treatment, defined as either complete or partial response according to masked investigator assessment, stable disease or progressive disease; 12-month overall survival and progression-free survival; safety; and efficacy (for progression-free survival and overall survival) according to tumour PD-L1 tumour proportion score. Quality of life (EQ-5D) and cost per QALY data were collected as part of the trial but are not reported here to allow for full follow-up to take place.

### Statistical analysis

Sample size was based on a hazard ratio (HR) of 0·7 for overall survival (equivalent to an improvement in median overall survival from 6·0 to 8·5 months), 80% power, 4 years of recruitment, and 6 months of follow-up. This led to a target sample size of 336 participants (291 events). A two-sided α of 0·04 was chosen based on interim analyses for efficacy for overall survival. One formal interim analysis for futility was carried out after 74 (25%) overall survival events had occurred in June, 2019, (median follow up 5·09 months (IQR 3·91–6·90). The study continued as planned after this interim analysis.

Almost 2 years into recruitment (Feb 14, 2019; protocol amendment 6), progression-free survival was added as a co-primary endpoint due to concerns that immunotherapy might be increasingly used off-study following progression, thus affecting the estimate of the effect of nivolumab on overall survival. Following the addition of progression-free survival as a co-primary endpoint, an α of 0·04 was maintained for overall survival, based on a hierarchical testing procedure,^10^ designed to maintain an overall α of 0·05 across the co-primary endpoints. This procedure used two α values to determine significance for progression-free survival depending on whether overall survival was significant (α 0·05) or not (α 0·01). The sample size of 336 participants gave more than 80% power for a HR of 0·65 for progression-free survival (with α 0·01). This change was approved by the independent masked Trial Steering Committee and was included in a protocol amendment 6.

On Jan 13, 2020, it was agreed, and approved by the independent masked Trial Steering Committee, that the preplanned interim efficacy and futility analyses should be removed (approximately 3 months before the anticipated end of recruitment; protocol amendment 7; June 11, 2020). The efficacy analysis was based on PD-L1 status, and recruitment was almost complete once sufficient samples were obtained and analysed. Interim futility analyses were removed due to them being done too near to or after the end of recruitment (as a consequence of faster than anticipated recruitment), restricting their perceived value. No other modifications were made to the study.

All statistical analyses were done with Stata (version 16.0). Investigator-reported progression-free survival and overall survival were analysed with a Cox proportional hazards model, adjusted for epithelioid type (because this was a stratification factor). Significance thresholds were 0·04 for overall survival, and either 0·05 if overall survival was significant or 0·01 if overall survival was not significant for progression-free survival. Survival curves for each group were estimated with the Kaplan-Meier method, and non-proportionality was assessed visually. Survival rates were derived from the Kaplan-Meier estimates. Prespecified sensitivity analyses were done to evaluate the predictive role of pre-study status with respect to PD-L1 expression defined as either positive or negative, using a group by expression interaction term in the Cox model. Response rates were compared with a crude odds ratio test (ie, no adjustment factors). Median time to onset of treatment-related adverse events and median time to resolution of treatment-related adverse events were assessed in a post-hoc analysis using the observed median time.

Both co-primary and secondary efficacy analyses and safety analyses include all participants who were randomly assigned. The only exception is for the PD-L1 analysis, for which only patients with assessable tissue samples were included. Analysis was done based on the treatment policy estimand, in which participants were analysed according to the group they were randomly assigned to and regardless of other treatments, such as off-trial immunotherapy (equivalent to the intention-to-treat principle). A prespecified analysis of progression-free survival and overall survival across prespecified baseline characteristics with forest plots was done. Median time to response and duration of response were included as post-hoc analyses. A prespecified efficacy analysis by PD-L1 subgroups were assessed for progression-free survival and overall survival.

This trial is registered with ClinicalTrials.gov, NCT03063450.

### Role of the funding source

The funder of the study, Cancer Research UK–Stand Up to Cancer, had no role in trial design, data collection, data analysis, data interpretation, or writing of the report. Bristol Myers Squibb provided nivolumab.

## Results

**Figure 1:**
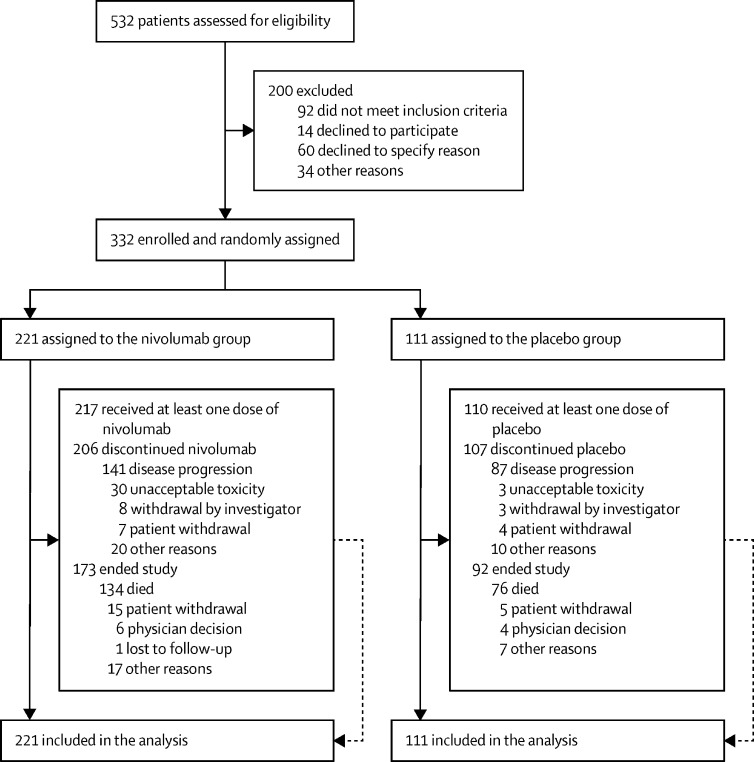
Trial profile

Between May 10, 2017, and March 30, 2020, we enrolled 332 participants, of whom 221 (67%) were assigned to the nivolumab group and 111 (33%) to the placebo group (figure 1). Recruitment was paused on March 30, 2020, when 332 people had been recruited, due to the COVID-19 pandemic. Following a COVID-19 impact review, the Independent Data Monitoring Committee and Trial Steering Committee did not require the trial to reopen to recruitment for the remaining four patients after the UK COVID-19 lockdown (March to June, 2020). This decision was made primarily due to the trial recruiting ahead of time, allowing for longer follow-up to reach the required number of events. Following a meeting in August, 2020, the Independent Data Monitoring Committee recommended the immediate release of preliminary data, approximately 1 year earlier than planned, for investigator-reported progression-free survival and overall survival. This decision was ratified by the Trial Steering Committee. We report the results of the preliminary analysis based on data collected from May 10, 2017, to Jan 4, 2021. All 332 participants were included in the analysis of the co-primary outcomes. The median follow-up (of those still alive on Jan 4, 2021) was 11·6 months (IQR 7·2–16·8).

The baseline characteristics of the patients are shown in table 1. The median age of the patients was 70 years (IQR 65–75). 253 (76%) of the 332 patients were male; 266 (80%) patients had an ECOG performance status score of 1; 316 (95%) patients had pleural mesothelioma; 293 (88%) patients had epithelioid histology; and 230 (69%) patients had been exposed to asbestos (table 1). PD-L1 data were available for 252 (76%) patients, of whom 86 (34%) were positive and 166 (66%) negative. All of the enrolled patients had received platinum-based therapy previously; 322 (97%) had received pemetrexed (appendix p 66).

**Table 1:** Baseline characteristics

		**Nivolumab (n=221)**	**Placebo (n=111)**
Median age, years (IQR)		70 (65–74)	71 (65–76)
Sex			
	Male	167 (76%)	86 (78%)
	Female	54 (24%)	25 (23%)
ECOG performance status score			
	1	177 (80%)	89 (80%)
	0	44 (20%)	22 (20%)
Smoking status			
	Ex-smoker	105 (48%)	52 (47%)
	Non-smoker	100 (45%)	52 (47%)
	Current smoker	15 (7%)	6 (5%)
	Missing	1 (<1%)	1 (1%)
Site of mesothelioma			
	Pleural	211 (95%)	105 (95%)
	Non-pleural	10 (5%)	6 (5%)
PD-L1 status			
	<1% (negative)	101 (46%)	65 (59%)
	≥1% (positive)	60 (27%)	26 (23%)
	Missing	60 (27%)	20 (18%)
Histology			
	Epithelioid	195 (88%)	98 (88%)
	Non-epithelioid	26 (12%)	13 (12%)
Asbestos exposure			
	Yes	150 (68%)	80 (72%)
	No	65 (29%)	30 (27%)
	Missing	6 (3%)	1 (1%)
Line of treatment			
	Second line	63 (29%)	37 (33%)
	Third line	124 (56%)	66 (60%)
	Later than third line	34 (15%)	8 (7%)
Time since mesothelioma diagnosis			
	Median time since diagnosis, months (IQR)	17·8 (11·7–27·4)	17·7 (10·9–25·7)
	Missing	0	1 (1%)

Data are n (%), unless otherwise stated.

217 (98%) of 221 patients in the nivolumab group and 110 (99%) of 111 patients in the placebo group received at least one dose of treatment. A median of six doses (IQR 3–12) of nivolumab and four doses of placebo (3–7) were administered. At least one dose delay occurred in 96 (44%) of the 217 patients in the nivolumab group and 34 (31%) of the 110 patients in the placebo group. Overall, 144 (8%) of the 1872 treatment cycles in the nivolumab group had delays and 45 (6%) of the 747 treatment cycles in the placebo group had delays. Of the participants who had dose delays, most patients had only one dose delay (66 [69% of 96 patients in the nivolumab group and 26 [77%] of 34 patients in the placebo group), ranging from 1 to 263 days in duration (appendix p 66).

12 (6%) of 217 patients in the nivolumab group and three (3%) of 110 patients in the placebo group completed protocol treatment. After discontinuation of treatment, 77 (35%) of 217 patients in the nivolumab group and 39 (35%) of 110 patients in the placebo group received subsequent systemic cancer therapy. In the placebo group, 12 (11%) of 111 patients received nivolumab following unmasking (requested by the clinical team primarily following progression).

**Figure 2:**
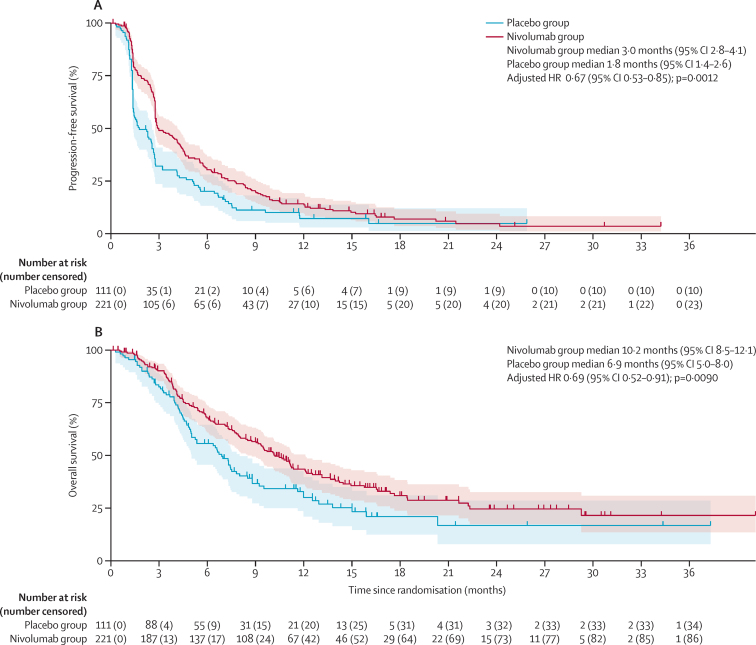
Kaplan–Meier curves of progression-free survival (A) and overall survival (B) HR=hazard ratio. Shaded areas represent 95% CIs.

**Figure 3:**
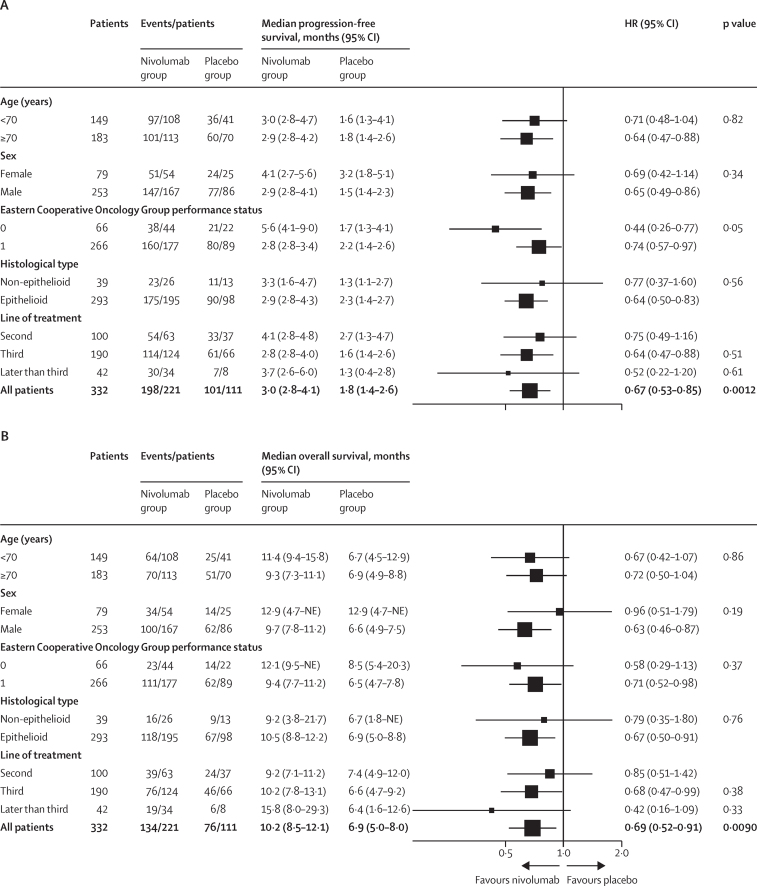
Forest plots showing subgroup analyses of progression-free survival (A) and overall survival (B) HR=hazard ratio. NE=not estimable.

At the time of the preliminary analysis database lock (Jan 4, 2021), a recorded date of death had been reported for 210 (63%) of the 332 participants who had undergone randomisation (72% of the 291 deaths required for the final analysis). 299 (90%) had investigator-reported dates of progression (more than the target of 284 [86%] patients). Median investigator-reported progression-free survival was 3·0 months (95% CI 2·8–4·1; events reported for 198 [90%] of 221 patients) in the nivolumab group, compared with 1·8 months (1·4–2·6; events reported for 101 [91%] of 111 patients) in the placebo group (adjusted HR 0·67 [95% CI 0·53–0·85]; p=0·0012; significance threshold of 0·05; figure 2A). Progression-free survival at 1 year was 14·2% (95% CI 9·9–19·3) in the nivolumab group versus 7·2% (3·1–13·8) in the placebo group. Progression-free survival in the prespecified subgroups of patients with epithelioid or non-epithelioid histology and by PD-L1 positivity status are shown in the appendix (pp 51–52, 55). Figure 3A shows a forest plot of progression-free survival across baseline characteristics.

Median overall survival was 10·2 months (95% CI 8·5–12·1; deaths reported in 134 [61%] of 221 patients) in the nivolumab group compared with 6·9 months (5·0–8·0; deaths reported in 76 [68%] of 111 patients) in the placebo group (adjusted HR 0·69 [95% CI 0·52–0·91]; p=0·0090; significance threshold of 0·04; figure 2B). Overall survival at 1 year was 43·4% (95% CI 36·3–50·4) in the nivolumab group versus 30·1% (21·0–39·6) in the placebo group. Overall survival in the prespecified subgroups of patients with epithelioid or non-epithelioid histology and by PD-L1 positivity status are shown in the appendix (pp 53–54, 56). Figure 3B shows a forest plot of overall survival across baseline characteristics.

For both progression-free survival and overall survival, the Kaplan-Meier plots were assessed visually for proportional hazards. Although there was a delay in divergence of the curves immediately following randomisation, the research team felt there to be no strong evidence of non-proportionality.

The overall response rate was significantly higher in the nivolumab group (25 [11%] of 221 patients had a partial response) than in the placebo group (one [1%] of 111 patients had a partial response; odds ratio 14·0 [95% CI 2·4–not estimable, p=0·00086; table 2). The median time to response was 84 days (95% CI 81–145) in the nivolumab group, and the median duration of response was 143 days (92–211). One (1%) patient in the placebo group partially responded with a time to response of 132 days, lasting a duration of 212 days.

**Table 2:** Responses to nivolumab and placebo

		**Nivolumab group (n=221)**			**Placebo group (n=111)**		
		PD-L1-positive patients (n=60)	PD-L1-negative patients (n=101)	Overall (n=221)*	PD-L1-positive patients (n=26)	PD-L1-negative patients (n=65)	Overall (n=111)*
Progressive disease		11 (18%)	25 (25%)	51 (23%)	7 (27%)	29 (45%)	46 (41%)
	Stable disease	34 (57%)	50 (50%)	117 (53%)	14 (54%)	33 (51%)	54 (49%)
	Partial response	7 (12%)	10 (10%)	25 (11%)	1 (4%)	0	1 (1%)
	Not evaluable	0	2 (2%)	2 (1%)	0	0	0
Missing		8 (13%)	14 (14%)	26 (12%)	4 (15%)	3 (5%)	10 (9%)

Data are n (%).

* All patients are included, including those with missing PD-L1 status.

252 (76%) of the 332 randomly assigned patients had quantifiable PD-L1 expression. On the basis of a positivity threshold of 1%, there was no evidence of PD-L1 expression being predictive of response to treatment for either overall survival (HR for interaction 1·16 [95% CI 0·56–2·40]; p=0·70) or progression-free survival (HR for interaction 1·55 [0·85–2·83]; p=0·16). A sensitivity analysis revealed no interaction between PD-L1 expression and clinical outcome (appendix pp 55–56).

Adverse events leading to treatment discontinuation occurred in 30 (14%) of 217 patients in the nivolumab group compared with three (3%) of 110 patients in the placebo group (appendix p 64). The most common adverse events that led to treatment discontinuation in the nivolumab group were infusion-related reactions (four [13%]) and diarrhoea (three [10%]). In the control group one (33%) patient discontinued due to pneumonia and one (33%) due to hyponatraemia; the adverse event was not reported for the third patient.

Serious adverse events occurred in 90 (41%) of 221 patients in the nivolumab group and 49 (44%) of 111 patients in the placebo group. The most frequent serious adverse events were dyspnoea (18 [8%] of 221 patients in the nivolumab group *vs* ten [9%] of 111 patients in the placebo group), pneumonia (14 [6%] *vs* six [5%]) and lower respiratory tract infection (eight [4%] *vs* eight [7%]; appendix pp 57–60). Causes of death related to any serious adverse events are summarised in the appendix (p 65). There were no treatment-related deaths in either group.

Treatment-related adverse events, including haematological and non-haematological events, occurred in 163 (74%) of 221 patients in the nivolumab group and 62 (56%) of 111 patients in the placebo group (table 3; appendix pp 61–63) No grade 5 events were reported in either group. The most frequently reported grade 3 or worse treatment-related adverse events diarrhoea (six [3%] of 221 in the nivolumab group *vs* two [2%] of 111 in the placebo group), and infusion-related reaction (six [3%] *vs* none).

**Table 3:** Treatment-related adverse events

	**Nivolumab group (n=221)**		**Placebo group (n=111)**	
	Grade 1–2	Grade 3	Grade 1–2	Grade 3
Anaemia	5 (2%)	1 (1%)	0	0
Thrombocytopenia	0	1 (1%)	0	0
Cardiac dysfunction	0	1 (1%)	0	0
Tachycardia	0	0	0	1 (1%)
Hypothyroidism	10 (5%)	1 (1%)	1 (1%)	0
Macular oedema	0	0	0	1 (1%)
Abdominal pain	2 (1%)	1 (1%)	1 (1%)	0
Ascites	1 (1%)	1 (1%)	0	0
Colitis	1 (1%)	3 (1%)	0	0
Colitis microscopic	0	1 (1%)	0	0
Diarrhoea	29 (13%)	6 (3%)	8 (7%)	2 (2%)
Nausea	32 (15%)	0	9 (8%)	0
Stomatitis	4 (2%)	2 (1%)	1 (1%)	0
Vomiting	9 (4%)	1 (1%)	4 (4%)	0
Chest pain	0	1 (1%)	0	0
Fatigue	59 (27%)	1 (1%)	20 (18%)	1 (1%)
Generalised oedema	0	1 (1%)	0	0
Malaise	1 (1%)	0	0	1 (1%)
Autoimmune hepatitis	0	2 (1%)	0	0
Hepatotoxicity	1 (1%)	1 (1%)	0	0
Infusion-related reaction	12 (5%)	6 (3%)	1 (1%)	0
Alanine aminotransferase increased	5 (2%)	4 (2%)	1 (1%)	0
Aspartate aminotransferase increased	5 (2%)	4 (2%)	0	0
Blood alkaline phosphatase increased	5 (2%)	4 (2%)	0	0
Blood bilirubin increased	4 (2%)	1 (1%)	1 (1%)	0
Blood creatinine increased	1 (1%)	0	2 (2%)	1 (1%)
Gamma-glutamyltransferase increased	0	1 (1%)	0	0
Lipase increased	0	1 (1%)	0	0
Hyponatraemia	0	0	0	1 (1%)
Arthralgia	13 (6%)	1 (1%)	5 (5%)	0
Arthritis	2 (1%)	1 (1%)	1 (1%)	0
Back pain	3 (1%)	1 (1%)	2 (2%)	0
Myositis	0	0	0	0
Ataxia	0	1 (1%)	0	0
Carpal tunnel syndrome	0	1 (1%)	0	0
Facial paralysis	0	1 (1%)	0	0
Headache	4 (2%)	1 (1%)	2 (2%)	0
Tremor	1 (1%)	1 (1%)	0	0
Anxiety	0	1 (1%)	0	0
Prostatism	0	0	0	1 (1%)
Chronic obstructive pulmonary disease	0	1 (1%)	0	0
Dyspnoea	15 (7%)	2 (1%)	5 (5%)	1 (1%)
Dyspnoea exertional	0	1 (1%)	0	0
Immune-mediated pneumonitis	0	1 (1%)	0	0
Pneumonitis	0	1 (1%)	0	0
Pulmonary embolism	1 (1%)	1 (1%)	0	0
Erythema	1 (1%)	1 (1%)	0	0
Pruritus	24 (11%)	0	10 (9%)	0

Data are n (%). Two grade 4 events were reported in the nivolumab group: one (1%) patient had grade 4 gamma-glutamyltransferase increase and one (1%) patient had grade 4 myositis. There were no grade 4 events reported in the placebo group.

Treatment-related serious adverse events also occurred in 28 (13%) of 221 patients in the nivolumab group and eight (7%) of 111 patients in the placebo group. Diarrhoea (five [2%] of 28 patients in the nivolumab group and three [3%] of eight patients in the placebo group) and infusion-related reactions (four [2%] of 28 patients in the nivolumab group and one [1%] of eight patients in the placebo group) were the most frequent treatment-related serious adverse events.

The most frequently reported immune-related treatment related adverse events of any grade were gastrointestinal (76 [34%] of 221 patients in the nivolumab group *vs* 29 [26%] of 111 patients in the placebo group) and skin (51 [23%] in the nivolumab group *vs* 14 [13%] in the placebo group). Median time to onset of treatment-related adverse events was 15 days (IQR 4–31) across categories in the nivolumab group and 11 days (2–26) in the placebo group.

The median time to resolution of treatment-related adverse events was 7 days (IQR 1–29) in the nivolumab group and 5 days (1–22) in the placebo group. The median time to onset of treatment-related adverse pulmonary events was 84 days (IQR 42–126) with a median time of resolution of 56 days (2–110) in the nivolumab group; there were no events in the placebo group. There were no recurrences of pneumonitis in both groups.

## Discussion

The CONFIRM trial showed longer progression-free survival and overall survival with nivolumab compared with placebo in patients with relapsed mesothelioma. To our knowledge, CONFIRM is the first randomised phase 3 trial to show significantly improved overall survival for patients with relapsed mesothelioma following platinum-based doublet chemotherapy. Crossover to immunotherapy in the placebo group was infrequent, probably because of the general scarcity of availability of off-label immune checkpoint inhibitors in the UK during this study. CONFIRM justifies the use of single drug anti-PD-1 inhibition in patients who have received first-line platinum-doublet therapy. These results also support the findings of other single group phase 1 trials, including the MERIT trial^7^ which led to the approval of nivolumab in Japan in 2018. The much larger size of the CONFIRM cohort, might account for any differences in outcome compared with the MERIT trial.

Patients, particularly those in the placebo group, with rapid disease progression and clinical deterioration were unable to attend a CT scan, especially in cases where this required longer travel (sometimes with a substantial distance) to the CONFIRM trial centre.

A key limitation of the study was the absence of centrally reviewed radiology, despite the use of progression-free survival as a co-primary endpoint; the study was not funded to include a central review. Radiological interpretation of progression (or response) in patients with mesothelioma is challenging, with the accuracy of assessment dependent on the use of modified RECIST criteria. Another limitation of CONFIRM was that most of the recruited patients were in the third line setting, given the current the absence of a post-platinum approved therapy. This was in part due to patients having had rechallenge platinum-based therapy or vinorelbine as a second-line treatment. Whether the efficacy of nivolumab could have differed if this study had been designed for a strictly second-line population remains unknown. For this reason, the results can only be extrapolated to the second-line setting.

This trial enrolled patients with peritoneal mesothelioma; there were several reasons for this decision. First, this group of patients is under-served in terms of access to novel drugs. Second, there are no data, to our knowledge, on the efficacy of single-drug anti-PD-1 or anti-PD-L1 immunotherapy in patients with relapsed peritoneal mesothelioma; this is probably because of the very small number of patients with the disease, which would make a placebo-controlled randomised trial in this group very challenging. Third, given the similar genomic landscape of both pleural and peritoneal mesothelioma (copy number-driven somatic alterations including high *BAP1* and *CDKN2A* inactivation frequency), there was no expectation of widely differing responses to anti-PD-1 checkpoint inhibition between these subtypes. The prognostic variation within the pleural subgroup (based on histology and genotype) is probably significantly higher than between pleural and peritoneal mesothelioma based on the DETERMINE trial.^4^

For the most common immune-related treatment related adverse events, nivolumab had additional toxicity in the order of 10%. For other treatment related adverse events, nivolumab was primarily seen to cause an increase in fatigue, diarrhoea, nausea, and rash, but the increases were generally modest (5–10%) compared with placebo.

The PROMISE meso trial,^11^ which reported its results in 2020, did not show superiority of the PD-1 immune checkpoint inhibitor, pembrolizumab, compared with chemotherapy (vinorelbine or gemcitabine) for overall survival. The choice of either vinorelbine or gemcitabine was based on previous single-group phase 2 studies, showing variable levels of useful activity.^12^, ^13^ So far, no randomised trial of vinorelbine has been reported in the relapsed setting. The VIM randomised phase 2 trial^14^ of vinorelbine versus active symptom control was reported to meet its primary endpoint of significantly improved progression-free survival. Gemcitabine showed a promising signal of activity in the post first-line chemotherapy setting as switch maintenance in the randomised phase 2 trial NVALT19.^15^ On the basis of the absence of a licenced therapy in the relapsed setting, placebo was chosen as the control for the CONFIRM trial.

The DETERMINE trial^9^ was a placebo-controlled, randomised, phase 2 trial of the anti-CTLA-4 immune checkpoint inhibitor, tremelimumab, in patients with relapsed mesothelioma. There was no effect on overall survival; however, ipilimumab (anti-CTLA-4) in combination with nivolumab versus nivolumab alone probably showed synergy in the relapsed setting in the non-comparative, randomised, phase 2 MAPS2 trial.^8^ Ipilimumab and nivolumab showed superiority compared with pemetrexed–platinum doublet in the first line setting in the Checkmate 743 phase 3 trial,^16^ with an overall survival HR of 0·74; ipilimumab and nivolumab combination therapy has since been approved by the US Food and Drug Administration and the European Medicines Agency (Oct 2, 2020) as first-line treatment of patients with unresectable malignant pleural mesothelioma. However, a subgroup analysis showed a larger overall survival benefit with nivolumab plus ipilimumab compared with chemotherapy for patients with non-epithelioid histology (HR 0·46 [95% CI 0·31–0·68]) than those with epithelioid histology (0·86 [0·69–1·08]),^16^ although the study was not powered to test superiority within subgroups.

By contrast, our prespecified subgroup analysis seemed to suggest superiority for nivolumab versus placebo in patients with epithelioid disease who had previously received platinum doublet regimens. This finding was not observed in patients with non-epithelioid mesothelioma, although this could be accounted for by the small number of patients with this relatively rare histological subtype, and the immature number of survival events in that subgroup at the time of this analysis. Furthermore, selection bias of patients with non-epithelioid mesotheliomas could arise if they did not survive long enough to enrol in the CONFIRM trial, highlighting the importance of treating these patients as early as possible with immunotherapy, as suggested by the Checkmate 743 trial.^16^

PD-L1 is an established predictive biomarker for immune checkpoint therapy in non-small-cell lung cancer.^17^ Robust evidence for PD-L1 as a predictive factor for PD-1 inhibition in mesothelioma is scarce.^11^ Overexpression of PD-L1 has been associated with a poor prognosis.^18^ Using the Dako 22C3 PD-L1 tumour proportion score above 1%, we found no evidence to support a role for PD-L1 as a predictive biomarker. However, caution is required in interpretation due to the immaturity of the data and the low PD-L1 expression (consistent with other studies employing the 22C3 antibody),^6^ which is underpowered to detect a statistically significant effect at more than 1% or more than 50% (appendix pp 52, 54–56). For this reason, use of a different threshold (eg, >50%) was not applied. Although pretreatment biopsies were used, there is no evidence for statistically significant changes in PD-L1 expression longitudinally following platinum-based chemotherapy.^19^ PD-L1 evaluation was done in 76% of participants in CONFIRM, in part due to diagnostic biopsies being either unevaluable or missing at the time of collection (which in some cases was after a substantial period following enrolment).

The cellular and molecular determinants of response to PD-1 checkpoint inhibition in mesothelioma remain elusive. Accordingly, extensive translational research studies have been initiated in CONFIRM to explore the genomic and tumour microenvironmental interactions with outcome and to understand the molecular determinants of sensitivity in mesothelioma. We will continue to follow-up participants in the CONFIRM trial for overall survival and progression-free survival, until the original planned study end (expected July, 2021). A final analysis is planned following completion of the study, which will include an updated analysis and a health economics analysis.

## Data sharing

Trial data relating to this publication shall remain confidential to the sponsor organisation and will not be disclosed, except when disclosure might be required in accordance with pharmacovigilance duties of the parties involved. Individual participant data can be made available, after deidentification, to investigators who provide a written request in accordance with General Data Protection Regulation and following authorisation from the sponsor organisation, starting immediately and ending 3 years after publication. Data sharing requests should be directed to DAF and GOG. Southampton Clinical Trials Unit (SCTU), University of Southampton, Southampton, UK, is committed to the responsible sharing of clinical trial data and trial samples with the wider research community. Data access is administered through the SCTU Data Release Committee. Requests for data access and sharing for SCTU trials should be emailed to the SCTU Data Release Committee Coordinator at ctu@soton.ac.uk.

## Declaration of interests

DAF reports grants from Astex Therapeutics, Boehringer Ingelheim, Merck Sharp & Dohme, and Bayer; personal fees from Aldeyra, Inventiva, and Paredox; non-financial support from Clovis, Eli Lilly, and Bristol Myers Squibb; and personal fees and non-financial support from Roche, during the study. GG reports grants from Jannsen-Cilag, Novartis, Astex, Roche, Heartflow, Bristol Myers Squibb, BioNtech; grants and personal fees from AstraZeneca; and personal fees from Celldex, outside the submitted work. JL reports grants from Cancer Research UK and non-financial support from Bristol Myers Squibb, during the study. CO reports personal fees from Bristol Myers Squibb, outside the submitted work. RC reports personal fees from Bristol Myers Squibb, Merck Sharp & Dohme, Roche, and AstraZeneca, during the study. All other authors declare no competing interests.
