# Correction to *Lancet Oncol* 2021; 22: 1530–40

**DOI:** 10.1016/S1470-2045(25)00221-9

**Published:** 2025-05

*Fennell DA, Ewings S, Ottensmeier C, et al. Nivolumab versus placebo in patients with relapsed malignant mesothelioma (CONFIRM): a multicentre, double-blind, randomised, phase 3 trial.* Lancet Oncol *2021; **22:** 1530–40*—The appendix of this Article has been corrected as of April 30, 2025.

