# Supplementary material for: Nivolumab versus placebo in patients with relapsed malignant mesothelioma (CONFIRM): a multicentre, double-blind, randomised, phase 3 trial
Source: Lancet Oncol. 2021 Nov;22(11):1530–40. doi: 10.1016/S1470-2045(21)00471-X (PMC8560642; doi:10.1016/S1470-2045(21)00471-X)
Supplement: Supplementary appendix [file mmc1.pdf]

THE LANCET
Oncology

Supplementary appendix

This appendix formed part of the original submission and has been peer reviewed. We post it as supplied by the authors.

Supplement to: Fennell DA, Ewings S, Ottensmeier C, et al. Nivolumab versus placebo in patients with relapsed malignant mesothelioma (CONFIRM): a multicentre, double-blind, randomised, phase 3 trial. *Lancet Oncol* 2021; published online Oct 14. http://dx.doi.org/10.1016/S1470-2045(21)00471-X.

12-month Survival - % (95% CI)	Nivolumab (n=221)	Placebo (n=111)	Total (n=332)
**PFS**	14.2%<br>(9.9%-19.3%)	7.2%<br>(3.1%-13.8%)	12.0%<br>(8.7%-19.9%)
**OS**	43.4%<br>(36.3%-50.4%)	30.1%<br>(21.0%-39.6%)	39.1%<br>(33.3%-44.7%)

Southampton Clinical Trials Unit

CONFIRM

Statistical Analysis Plan

Trial name:	CONFIRM
Trial registration number:	EudraCT: 2016-003111-35
Protocol title and version number:	**C**heckp**O**i**N**t blockade **F**or **I**nhibition of **R**elapsed **M**esothelioma (CONFIRM): A Phase III Double-Blind, Placebo Controlled Trial to Evaluate the Efficacy of Nivolumab in Relapsed Mesothelioma v7
SAP version number:	V1.0
SAP date:	26/10/2020

To be approved and reviewed by:

	Name	Signature	Date
**Chief Investigator**	Professor Dean Fennell	[signature]	27/10/2020
**Trial Statistician (author)**	Kayleigh Hill		
**Senior Clinical Trial Statistician**	Sean Ewings		

Table of Contents

List of Abbreviations 3
Keywords 4
1 Introduction 5
1.1 Purpose of SAP 5
1.2 Trial Personnel 5
1.3 Trial background and rationale (short synopsis) 6
1.4 Objectives 6
1.5 Definition of endpoints 6
1.6 Analysis principles 7
2 Design considerations 8
2.1 Description of trial design 8
2.2 Trial power and sample size 8
2.3 Randomisation details 9
2.4 Timing of planned analyses 9
***Statistical considerations* 10**
2.5 Definition of analysis populations 10
2.6 Analysis software 10
2.7 Methods for handling data 10
2.8 Definition of key derived variables 11
2.9 General principles for reporting and analysis 11
3 Planned analyses and reporting 12
3.1 Disposition of the study population 12
3.2 Protocol deviations and unblinding summary 12
3.3 Post-study therapy 12
3.4 Baseline characteristics 12
3.5 Treatment Information 13
3.6 Primary endpoints 13
3.7 Secondary endpoints 14
3.8 Translational endpoints 14
3.9 Safety reporting 14
4 Tables, listings and figures templates 16

4.1 List of tables, figures and listings 16
4.2 Patient disposition 17
4.3 Protocol deviations and unblinding 19
4.4 Demographic and baseline characteristics 21
4.5 Treatment information 24
4.6 Primary Endpoint Analysis 25
4.7 Other Secondary Endpoint Analysis 28
4.8 Biomarker subgroup Analysis 30
4.9 Safety reporting 37

References 46

5 SAP revision history 47

List of Abbreviations

Abbreviation		Abbreviation	
AACR	American Association of Cancer Research	MHRA	Medicines and Healthcare products Regulatory Agency
AE	Adverse Event	NCI	National Cancer Institute
ALT	Alanine aminotransferase	NHS IC	National Health Service Identification Centre
AR	Adverse Reaction	NSCLC	Non-small cell lung cancer
AST	Aspartate aminotransferase	ORR	Objective response rate
BMS	Bristol-Myers Squibb	PR	Partial response
BP	Blood pressure	Q2W	Every two weeks
BUN	Blood urea nitrogen	REC	Research Ethics Committee
CI	Chief Investigator	RR	Respiratory rate
CRF	Case Report Form	SAE	Serious Adverse Event
CTA	Clinical Trial Authorisation	SAP	Statistical Analysis Plan
CTCAE	Common Terminology Criteria for Adverse Events	SAR	Serious Adverse Reaction
DMP	Data Management Plan	SCNA	Somatic copy number alteration
ECOG	Eastern Cooperative Oncology Group	SCTU	Southampton Clinical Trials Unit
EQ-5D-5L	European Quality of Life-5 Dimensions-5 Levels	SUSAR	Suspected Unexpected Serious Adverse Reaction
FFPE	Formalin-fixed, paraffin-embedded	TMF	Trial Master File
GCP	Good Clinical Practice	TMG	Trial Management Group
HR	Hazard Ratio	TSC	Trial Steering Committee
IASLC	International Association for the Study of Lung Cancer	TSH	Thyroid-stimulating hormone

IB	Investigator's Brochure	T3	Triiodothyronine
IDMC	Independent data monitoring committee	T4	Thyroxine
IMP	Investigational Medicinal Product	UAR	Unexpected Adverse Reaction
ISF	Investigator Site File	ULN	Upper limits of normal
LDH	Lactate dehydrogenase	WOCBP	Women of childbearing potential

Keywords

Mesothelioma; anti PD-1; Nivolumab; immunotherapy; RECIST; quality of life; survival; immune checkpoint inhibition; PD-L1

1 Introduction

1.1 Purpose of SAP

The independent data monitoring committee (IDMC) met on 13th August 2020 and recommended early release of the progression free survival (PFS) and overall survival (OS) results; this was later ratified by the Trial Steering Committee (TSC). The use of investigator reported PFS (i.e., not independently confirmed PFS, through review of RECIST measures) was agreed by the Trial Management Group (TMG), and the TSC chair and statistician, to be a meaningful measure of PFS, and should form the basis of the early PFS results given the incomplete RECIST data at the time. It was agreed by the IDMC and TSC that OS results could be released even if had not reached its required number events.

This statistical analysis plan (SAP) describes in detail the methods that will be used to analyse the data collected as part of the phase III double-blind, placebo controlled CONFIRM trial for the International Association for the Study of Lung Cancer (IASLC) abstract submission and presentation. This will form the basis of the preliminary data trial publication to be submitted in 2021. The final analysis of mature data (based on overall survival, with additional analysis based on RECIST-guided PFS) will follow a new updated SAP. Both this and the future SAP will ensure that the analyses are conducted in a scientifically valid manner and to avoid post hoc decisions that may affect the interpretation of the statistical analysis. Any deviations from the SAPs will be detailed in the preliminary publication and final report/publication. A Health Economics Analysis Plan will be developed separately to this SAP.

1.2 Trial Personnel

**Chief Investigator:**	**Professor Dean Fennell**<br>**Tel:** 0116 252 3170<br>**Email:** df132@le.ac.uk<br><br>**Professor Gareth Griffiths (Co-Chief Investigator)**<br>**Tel:** 023 8120 6184<br>**Email:** G.O.Griffiths@soton.ac.uk
**Co-Investigators:**	**Dr Jason Lester, Velindre NHS Trust**<br>**Dr Gerard Hanna, Queen's University Belfast**<br>**Dr Nicola Steele, Beatson West Of Scotland Cancer Centre**<br>**Dr Peter Szlosarek, Queen Mary, University Of London**<br>**Professor Sarah Danson, University Of Sheffield**<br>**Professor Joanne Lord, University Of Southampton**<br>**Professor Christian Ottensmeier, University Of Southampton**
**Trial Statistician:**	**Kayleigh Hill**<br>**Tel:** 023 8120 5689<br>**Email:** K.Hill@soton.ac.uk
**Senior Statistician(s):**	**Sean Ewings**<br>**Tel:** 023 8120 5674<br>**Email:** sean.ewings@soton.ac.uk

1.3 Trial background and rationale (short synopsis)

Effective therapy for relapsed mesothelioma is an unmet need. Despite a significant number of clinical studies in the second line setting, no randomised study to date has been positive. The James Lind Alliance Priority Setting Partnership funded by the NIHR has identified immunotherapy as the number one UK research priority. To date there have been no placebo controlled randomised trials for mesothelioma using PD-L1 or PD-1 checkpoint inhibition.

Early promising signals of activity relating to both PD-L1 and PD-1 targeted treatment in mesothelioma implicate a dependency of mesothelioma on this immune checkpoint, and support the development of a randomised phase III trial to evaluate the efficacy of nivolumab. CONFIRM will be the first ever placebo controlled, randomised phase III trial of a PD-1 immune checkpoint inhibitor.

PD-1 checkpoint inhibition has revolutionised the treatment of melanoma and is now standard of care in non-small cell lung cancer, squamous cell cancer head and neck and classical Hodgkin's lymphoma. It is being assessed rigorously in numerous other cancers making its evaluation in mesothelioma timely in CONFIRM.

1.4 Objectives

Primary objectives:

- To compare overall survival (OS) of nivolumab with placebo in patients with relapsed mesothelioma (co-primary endpoint)
- To compare progression-free survival (PFS) of nivolumab with placebo, as determined by investigator (PFSi; co-primary endpoint)

Secondary objectives:

- To compare objective response rate (ORR), as determined by investigator, of nivolumab to placebo
- To compare progression-free survival (PFS), of nivolumab to placebo, based on mRECIST and RECIST1.1 (PFSr)
- To determine the safety profile of nivolumab in relapsed mesothelioma patients

Translational objectives:

- To define the association of PD-L1 status with PFS and OS in patients with relapsed mesothelioma
- To correlate:
    - a) Mutation burden with OS
    - b) Immunosuppressive landscape (immune checkpoint expression and infiltration of immune cell) and nivolumab efficacy

1.5 Definition of endpoints

1.5.1 Definition of primary endpoint

Overall survival is defined as time from randomisation to death from any cause.

Progression-free survival (investigator reported) is defined as time from randomisation to progression (according to investigator reported progression), or death from any cause (whichever event comes first).

1.5.2 Definition of secondary endpoints

- **Progression-free survival** (based on mRECIST and RECIST1.1) is defined as time from randomisation to progression (according to modified RECIST or RECIST 1.1 of target lesion, assessment/appearance of non-target lesion), or death from any cause (whichever event comes first).

N.B. mRECIST or RECIST 1.1 will be assessed at week 6 (±3 days) and week 12 (±3 days) (regardless of the number of cycles of treatment the patient has received and whether trial treatment has stopped early for reasons other than progression).

Toxicity will be assessed using CTCAE v4.03 at baseline, after each treatment cycle, and for 100 days post treatment discontinuation and for ongoing drug-related AEs until resolved, return to baseline or deemed irreversible.

1.5.3 Definition of translational endpoints

Subgroup analysis of response to nivolumab according to expression of PD-L1: PD-L1 expression (<1%, 1-49%, ≥50%; and positive (≥1%) or negative (<1%)), will be determined by immunohistochemistry and sensitivity to nivolumab assessed by group.

Correlation between mutational burden and immune gene regulation profile and overall survival: this will be determined using genomic and transcriptomic analysis.

An analysis plan for mutational burden and gene regulation will be developed separate to this SAP.

1.6 Analysis principles

All analyses will be reported according to CONSORT (2010) and Southampton Clinical Trials Unit (SCTU) standard operating procedure (SOP) on planning, implementing and reporting statistical analyses (CTU/SOP/5058).

2 Design considerations

2.1 Description of trial design

A double blind, placebo controlled randomised phase III trial comparing nivolumab (anti PD-1 antibody) monotherapy 240mg every two weeks versus placebo. The treatment allocation ratio will be 2:1 in favour of nivolumab.

Patients will receive treatment with nivolumab at a dose of 240mg (or placebo) as a 30-minute IV infusion, on Day 1 (±2) of every 14-day treatment cycle, until progression, unacceptable toxicity, withdrawal of consent, or the maximum treatment duration of 12 months is reached, whichever occurs first. There will be no dose escalations or reductions of IMP allowed. Patients may be dosed no less than 12 days from the previous dose.

2.2 Trial power and sample size

The original sample size calculation was based on overall survival (OS) only (see full details below). This calculation accounted for a number of interim analyses (efficacy and futility) by using an alpha of 0.04 for the sample size (and ultimately the interpretation). During the course of the trial, the independent Trial Steering Committee (TSC) approved the inclusion of progression-free survival (PFS) as a co-primary endpoint in order to mitigate against the risk of treatment cross-over impacting the trial results, on the condition of maintaining the original sample size for OS. The analysis was planned to follow the Fallback procedure [25], though no explicit correction or acknowledgement of the impact of the analysis (where alpha is split across the endpoints) on sample size was initially proposed.

Following a later review by the TSC (meeting date 13th January 2020), all future interim analyses (which were formal rule-based efficacy and futility analyses) were removed; this decision was taken for three reasons: 1) a subset of the interim analyses were based on the PD-L1 positive subgroup, but analyses based on this could not take place until after recruitment was complete (due to delays in collecting samples and processing the results), lessening the potential benefit of carrying out these analyses; 2) the percentage of the samples that were PD-L1 positive was lower than anticipated at the start of the trial (due to changes in how the analysis is carried out using an approved 22C3 antibody assay - meaning a greater number of patients would need to be recruited to obtain a sufficient number who were PD-L1 +ve to conduct the analysis); and 3) the futility analyses would also not take place until very near the end (or after) the recruitment phase had completed. Due to 1) and 2) above it was not possible to undertake the PD-L1 +ve analysis during the recruitment period of the trials and as there was also little benefit in being able to stop the study for futility after the recruitment period, the TSC were keen to allow the study to run to completion. (Note: one analysis for harm had been carried out prior to this meeting, which was not relevant for the original sample size calculation.)

It was agreed that removing the formal interim analyses and accounting for the Fallback procedure in the final analysis would have no net effect on the sample size calculation, meaning the original sample size calculations hold. Although the original sample size calculation accounted for interim analyses that have been subsequently removed, the Fallback procedure was not originally accounted for. The result is that an alpha of 0.04 is to be used for the analysis of OS (as originally proposed), and, according to the Fallback procedure, an alpha for PFS of 0.05 (if OS null hypothesis is rejected) or 0.01 (if OS null hypothesis is not rejected). This approach maintains an overall 5% type 1 error rate across the co-primary endpoints, and will serve the purpose of demonstrating a treatment effect on either OS or PFS that is sufficient to establish clinical benefit; i.e., if the analysis of OS shows a statistically significant effect (at 0.04) this will be sufficient to establish clinical benefit (irrespective of whether PFS is

statistically significant or not), but if OS is non-significant, clinical benefit may still be established if PFS is statistically significant (at remaining unused alpha level of 0.01).

The independent data monitoring committee (IDMC) met on 13th August 2020 and recommended early release of the PFS and OS results, subsequently ratified by the TSC (who were unblinded at this point on order to review the relevant IDMC reports). It was agreed between the TMG and TSC that investigator reported PFS would be an appropriate definition of PFS, given that the study was blinded and that this was more likely to reflect clinical decision-making (e.g., stopping of treatment).

2.2.1 Power calculation for Overall Survival

Based on the VANTAGE trial [20] the expected median survival of patients on placebo is approximately 6 months.

Sample size assumptions (using artsurv in Stata): Based on a hazard ratio of 0.70 (equivalent to extending the median overall survival rate from 6 months to 8.5 months, or increasing the 6 months overall survival rate from 50% to 61.5% - considered a clinically significant difference by the research team); 80% power; recruitment period of 4 years, then 6 months follow-up period; 2-sided significance level of 4% (based on Fallback procedure); negligible drop out. Number of patients required: 336 (224 in the experimental arm and 112 in the control arm), a total of 291 events (deaths).

2.2.2 Power calculation for Progression Free Survival

Based on the DETERMINE trial [24] the expected median PFS of patients on placebo is approximately 3 months.

The original sample size of 336 patients will also provide 80% power to detect a hazard ratio of 0.65 (equivalent to extending the median PFS from 3 months to 4.6 months or increasing the 3 month PFS rate from 50% to 63.7% - considered a clinically significant difference by the research team); with recruitment period of 4 years; 6 months follow-up period; 2-sided significance level of 1% (remaining unused alpha from Fallback procedure for 5% level overall); negligible drop out. This will require a total of 284 events (progression or deaths) (calculated using artsurv in Stata).

2.3 Randomisation details

Patients will be randomised to either nivolumab or the control arm on a 2:1 allocation. Patients will be stratified according to epithelioid versus non-epithelioid.

2.4 Timing of planned analyses

2.4.1 Interim analyses and early stopping

As per protocol v7, there are no planned stopping guidelines for this trial.

2.4.2 Preliminary data analysis

The independent data monitoring committee (IDMC) met on 13th August 2020 and recommended early release of the progression free survival (PFS) and overall survival (OS) results; this was later ratified by the Trial Steering Committee (TSC). The use of investigator reported PFS (i.e., not independently confirmed PFS, through review of RECIST measures) was agreed by the Trial Management Group (TMG), and the TSC chair and statistician, to be a meaningful measure of PFS, and should form the basis of the early PFS results given the incomplete RECIST data at the time. It was agreed by the IDMC and TSC that OS results could be released even if had not reached its required number events.

2.4.3 **Final data analysis**

This will be documented in a final SAP once OS data is mature and RECIST PFS is clean; this is anticipated in Q2/3 2021 as per the original study timelines. This SAP will form the basis of the final SAP, and differences between the two will be documented in the final SAP.

Statistical considerations

2.5 Definition of analysis populations

2.5.1 Intention-to-treat analysis population

This population consists of all patients who have consented and been randomised to a treatment arm. All summaries and analyses will be on the ITT population unless otherwise specified.

2.5.2 Safety analysis population

This population consists of the ITT population who have received at least one dose of treatment.

2.6 Analysis software

All analyses will be carried out using STATA v16 or higher and/or SAS v9.4 or higher.

2.7 Methods for handling data

2.7.1 Withdrawal from trial

All data up until the point of patient withdrawal from the trial will be used in analyses unless the patient withdrew consent and does not wish for the data already collected prior to withdrawal to be used for the trial. Participants who initially consented to be registered for patient flagging will remain on the system so that important research information on date and cause of death can be requested from NHS Digital.

2.7.2 Missing data

The default position is that no imputation of missing or incomplete data is planned for the primary or secondary analyses, as PFS and OS are expected to largely be complete.

2.7.3 Outliers

There is no method planned for the handling of outliers in the data for the primary or secondary analyses.

2.7.4 Assumption checking and alternative methods

The proportional hazards assumptions underlying the Cox regression models described will be checked by plotting the log cumulative hazard against log time, which will yield roughly parallel lines for each study arm should the proportional hazards assumption be valid. Schoenfeld residuals will also be examined.

If the proportional hazards assumption does not appear to hold, other statistical methods will be explored, e.g., time-varying Cox model (e.g. piecewise model) and/or flexible parametric model.

2.7.5 Data transformations

There are no data transformations planned for this study.

2.8 Definition of key derived variables

2.8.1 Time since historical diagnosis of mesothelioma

Time in months from the date of historical diagnosis of mesothelioma to the date of the baseline visit.

2.8.2 Progression-free survival (PFS investigator reported; PFSi)

PFSi is defined as time from randomisation to progression (according to investigator reported progression), or death from any cause (whichever event comes first). Those alive with no investigator reported progression will be censored at the time last seen (or spoken to, if a COVID-19 telephone consultation took place).

2.8.3 Overall Survival (OS)

Overall survival is defined as time from randomisation to death from any cause. Those alive will be censored at the time last seen (or spoken to if a COVID-19 telephone consultation took place).

2.9 General principles for reporting and analysis

The following principles should be applied:

- A 2-sided 95% confidence interval (CI) will be presented for the primary analysis of the primary endpoint, alongside p-values. The co-primary endpoints will be assessed under the Fallback procedure, designed to control overall type 1 error rate. A difference in OS will be considered statistically significant for $p \leq 0.04$. Dependent on the result for OS, PFSi will be considered statistically significant at either $p \leq 0.01$ (if OS not statistically significant) or $p \leq 0.05$ (if OS is statistically significant).
- For all other analyses, 2-sided 95% CIs, p-values, and 5% significance level will be applied, unless otherwise stated.
- There will be no adjustments for multiplicity, besides those applied through the Fallback procedure for the co-primary endpoints.
- Descriptive statistics will be presented as appropriate to the nature of the data. For example, continuous variables will usually be summarised by the number of observations, mean and standard deviation (or median and IQR if the data appears skewed/not normal), minimum, and maximum. Categorical variables may be summarised by frequency counts and percentages for each category.
- Unless otherwise stated, percentages will be presented to one decimal place.
- For continuous data, the mean, standard deviation, median and quartiles will be rounded to one additional decimal place compared to the original data. Minimum and maximum will be displayed with the same accuracy as the original data.
- Unless otherwise stated, baseline data will be defined as the last available recorded measurement prior to administration of the first treatment on cycle 1 day 1.
- The labelling of the study arms will be as follows:
  - Nivolumab for the Nivolumab treatment group
  - Placebo for the Placebo treatment group
- In the case of partial dates, the RAVE imputed date will be used (1st Month/Jan).

3 Planned analyses and reporting

3.1 Disposition of the study population

Patient disposition will be summarised in a CONSORT flow diagram, showing a clear account of all patients who entered the study. These will be based on the enrolled population and will include:

- The number of patients assessed for eligibility and reason for not randomising
- The start and stop date of recruitment
- The number of patients randomised to each study arm
- The number of patients in the ITT population
- The number of patients in the safety population (who received at least one dose of study medication)
- The number of patients with ongoing treatment (Nivolumab/ Placebo treatment) & reasons for discontinuation (a patient is considered to be ongoing if they have not had the EOT visit)
- The number of patients ongoing (in the study) and reasons for end of study
- Duration of follow-up (median and 95% CI follow-up obtained from reversing the OS endpoint, where death counts as being censored) will be presented if <60% of participants have reached the OS endpoint

The disposition and treatment information above will also be summarised, along with the following:

- Stratification factors
  - The number of patients in each arm with epithelioid vs non-epithelioid mesothelioma
- Subgroup populations
  - The number of patients in each arm by PD-L1 status

3.2 Protocol deviations and unblinding summary

Any major Corrective And Preventative Actions (CAPAs) reported to the SCTU will be listed. Unblindings will be summarised by arm, alongside reasons, reported as frequencies and percentages.

3.3 Post-study therapy

Treatment received post-study will be summarised by arm, focusing on chemotherapy and immunotherapy, reported as frequencies and percentages.

3.4 Baseline characteristics

- Age
- Sex
- ECOG performance status
- Histology (epithelioid versus non-epithelioid)
- Asbestos exposure (yes/no)
- Smoking status
- Time since histological diagnosis of mesothelioma
- Stage
- Extra-thoracic metastases
- Site of mesothelioma (pleural or non-pleural)
- Line of treatment

3.5 Treatment Information

- Total duration of treatment received (minutes)
- Dose delay
- Dose interruptions
- Number of days on treatment

3.6 Primary endpoints

3.6.1 Overall Survival (OS)

OS is one of two co-primary endpoints in this trial, and the first in the hierarchy to be tested according to the Fallback procedure used in the study. Analysis will be based on the ITT population. The potential for participants to become unblinded (usually after progression, with the aim of seeking further treatment beyond the trial) is classed an intercurrent event, according to the ICH E9 addendum on estimands and sensitivity analysis. Using the ITT population is equivalent to the treatment policy strategy, as defined by this addendum.

The co-primary analysis will be based on OS compared between study arms using a Cox proportional hazards regression model adjusted for the stratification factor (epithelioid vs. non-epithelioid) and line of therapy. The p-value associated with study arm will be determined from this model together with the estimated hazard ratio and associated 95% 2-sided confidence interval. If the observed OS difference in favour of Nivolumab (based on the hazard ratio) is statistically significant at the 2-sided 4% level, this will be considered a signal of clinical benefit.

OS will also be presented graphically using a Kaplan-Meier (K-M) plot, with survival curves for each arm. In addition, the median OS and 12-month OS survival probabilities, and associated 95% CI, will be obtained. A reverse K-M will be applied to obtain the median maturity follow-up (if <60% of participants have reached the OS endpoint).

The proportional hazards model assumption will be evaluated, as described in section 2.7.4. The use of parametric models (e.g., Weibull) will be considered if there is evidence of non-proportional hazards.

The primary analysis will be repeated with an interaction term for PD-L1 status, to assess if the positive group are more likely to respond to treatment. This model will include PD-L1 status as a main effect.

3.6.2 Progression-Free Survival (PFS) - investigator reported

PFSi is the second co-primary endpoint according to the hierarchy of the Fallback procedure used in the study. It will be analysed according to the ITT principle. Non-adherence to treatment may be an intercurrent event, but the treatment strategy policy is the targeted estimand.

The co-primary analysis will be based on PFSi compared between study arms, using a Cox regression model adjusted stratification factor (epithelioid vs. non-epithelioid) and line of therapy. The p-value associated with study arm will be determined from this model together with the estimated hazard ratio and associated 95% 2-sided confidence interval. Analysis will follow the Fallback procedure, where the level used to judge statistical significance for PFS will depend on the result for OS. OS will be judged at an alpha of 0.04, and PFSi will be 0.05 or 0.01 depending on the statistical significance or not, respectively, of OS. If the observed PFSi difference

in favour of Nivolumab (based on the hazard ratio) is statistically significant at the appropriate level based on the OS result, this will be a signal of clinical benefit.

PFS will also be presented graphically using Kaplan-Meier plots. In addition, the median PFS and 12-month PFS survival probabilities, and associated 95% CI, will be obtained. A reverse K-M will be applied to obtain the median maturity follow-up (if <60% of participants have reached the PFSi endpoint).

The proportional hazards model assumption will be evaluated, as described in section 2.7.4. The use of parametric models (e.g., Weibull) will be considered if there is evidence of non-proportional hazards.

The primary analysis will be repeated with an interaction term for PD-L1 status, to assess if the positive group are more likely to respond to treatment. This model will include PD-L1 status as a main effect.

3.7 Secondary endpoints

- Best response – determined by the best response (complete response, partial response, stable disease progression) while on treatment; frequencies and percentages will be reported with corresponding 95% CIs for the latter, based on the Wilson Score method.
- Progression-free survival based on mRECIST and RECIST1.1. Will be analysed as per for PFS (investigator reported) above without p-value criteria.
- Treatment compliance: cycles and duration of treatment will be summarised based on the information available in the Nivolumab/Placebo administration forms, first date of treatment and last dose information. The start of treatment is defined as cycle 1 when the first dose is administrated. End of treatment information will be summarised as:
    - Number of patients who received at least one dose (Nivolumab/Placebo)
    - Discontinued Nivolumab/Placebo and reason for discontinuation

  Treatment duration will be summarised as follows:
    - The total number of cycles received
    - The median duration of treatment of Nivolumab or placebo (cycle 1 day 1 to last dose, inclusive)
    - Number of patients who had at least one Nivolumab /Placebo dose reduction
    - Number of patients who had at least one Nivolumab/Placebo dose missed

3.7.1 Quality of Life and Health Economic data

Health economics analysis will be described in a separate analysis plan.

3.8 Translational endpoints

The co-primary analyses will be repeated with the addition of PD-L1 status in two ways (1: positive [1+%] or negative [<1%]; 2: high positive [50+%], low positive [1-49%] and negative [<1%]). The role of PD-L1 status will be explored through the use of an interaction with treatment term in the Cox models.

Further translational analyses will be described in a separate analysis plan.

3.9 Safety reporting

All safety analyses will be performed on the safety population unless otherwise stated. Further summaries other than those described below (e.g. AEs leading to discontinuations, AE's leading to deaths, AE causality or other displays) may be reported at the discretion of the CI, following review.

3.9.1 Adverse events

Overall toxicity will be summarised by arm using frequencies and percentages. This will involve summarising the worst grade experienced by participants, the number of participants experiencing at least one AE, and the number of participants experiencing an AE by preferred terms and system organ class according to MedDRA coding.

3.9.2 Severe Adverse Events

For each study arm, the number of patients that experienced at least one AE graded 3 or above will be summarised overall, by system organ class (SOC) and preferred term. Patients with any AE graded 5 will be noted in the footnote of the table.

3.9.3 Serious Adverse Events

Serious toxicity includes serious adverse events (SAEs), serious adverse reactions (SARs) and suspected unexpected serious adverse reactions (SUSARs). In this section the term “serious adverse events (SAEs)” encompasses SAEs, SARs, SUSARs.

The total number of SAEs, the number of patients that experienced at least one SAE and the number of SAEs per patient (for patients experiencing at least one SAE, median and range) will be presented by study arm. The PI assessment, the number of SAEs by CTCAE grade and the reason for their seriousness will be presented for all SAEs by study arm.

The number of patients with at least one SAE will be summarised by system organ class (SOC) and preferred term for each study arm. This table will also be presented for SAEs grade 3 and above.

4 Tables, listings and figures templates

4.1 List of tables, figures and listings

Figure 1 CONSORT flow diagram of patient disposition 17
Figure 2 Kaplan-Meier plot for overall survivial by treatment arm 26
Figure 3 Kaplan-Meier plot for progresion free survivial (investigator reported) by treatment arm .. 28
Figure 4 Kaplan-Meier plot for overall survivial by treatment arm and PD-L1 status 32
Figure 5 Kaplan-Meier plot for progression free survivial by treatment arm and PD-L1 status 35

Table 1 Disposition of patients, End of Study 18
Table 2 Major protocol violations (excluding occurrences of unblinding) 19
Table 3 Unblindings summary (by arm) (as of xx-xxx-xxxx) 20
Table 4 Demographic information recorded at Baseline 21
Table 5 Disease Characteristics 22
Table 6 Treatment Information 24
Table 7 Overall survival information, including Cox models 25
Table 8 Progression-free survival (investigator reported) information, including Cox models 27
Table 9 Progression-free survival (determined by RECIST) information, including Cox models 28
Table 10 Progression data by arm 29
Table 11 Overall survival information for the PD-L1 subgroup, including Cox models 30
Table 12 Overall survival information for the PD-L1 subgroup, including Cox models 31
Table 13 Progression free survival information for the PD-L1 subgroup, including Cox models 33
Table 14 Progression free survival information for the PD-L1 subgroup, including Cox models 34
Table 15 Progression data by arm .... **Error! Bookmark not defined.**
Table 16 Overall toxicity by CTCAE Grade 37
Table 17 Overall Toxicity 37
Table 18 Overall Toxicity (Grade 3 or above) 39
Table 19 Summary of the SAEs reported 41
Table 20 Summary of the main symptom(s) reported on the SAE form 42
Table 21 Summary of the main symptom(s) reported on the SAE form (Grade 3 or above) 44

4.2 Patient disposition

Figure 1 CONSORT flow diagram of patient disposition

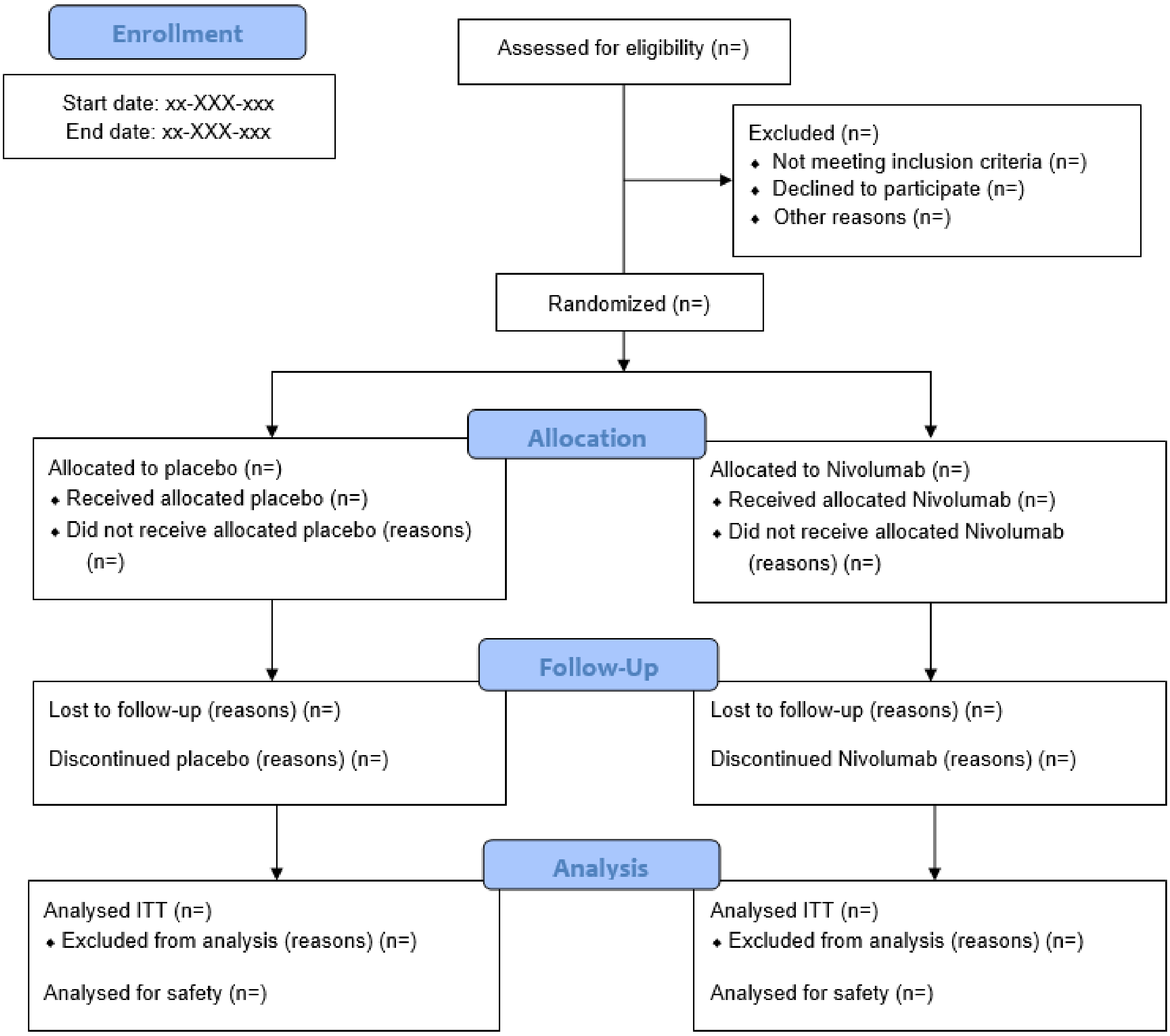

Table 1 Disposition of patients, End of Study

End of Study	Nivolumab (n=xx)	Placebo (n=xx)	Total (n=xx)
**Number of subjects ongoing[1]**	**xx (xx.x%)**	**xx (xx.x%)**	**xx (xx.x%)**
Primary reason for end of study			
**Completed**	**xx (xx.x%)**	**xx (xx.x%)**	**xx (xx.x%)**
**Death**	**xx (xx.x%)**	**xx (xx.x%)**	**xx (xx.x%)**
[Reasons]	xx (xx.x%)	xx (xx.x%)	xx (xx.x%)
**Lost to follow-up**	**xx (xx.x%)**	**xx (xx.x%)**	**xx (xx.x%)**
…	xx (xx.x%)	xx (xx.x%)	xx (xx.x%)
**Physician's decision**	**xx (xx.x%)**	**xx (xx.x%)**	**xx (xx.x%)**
…	xx (xx.x%)	xx (xx.x%)	xx (xx.x%)
**Withdrawal by subject**	**xx (xx.x%)**	**xx (xx.x%)**	**xx (xx.x%)**
…	xx (xx.x%)	xx (xx.x%)	xx (xx.x%)
Missing from eCRF – n(%)[3]	xx (xx.x%)	xx (xx.x%)	xx (xx.x%)

Note: Percentages are based on the number of patients per arm in the population

[1] Patients without EOS information and therefore remain in the study

4.3 Protocol deviations and unblinding

Table 2 Major protocol violations (excluding occurrences of unblinding)

Violation	Patient/site affected (if applicable)	Comments	Actions

Table 3 Unblindings summary (by arm) (as of xx-xxx-xxxx)

Characteristic	Nivolumab (n=xxx)	Placebo (n=xxx)	Total (n=xxx)
**No. of patients unblinded – n(%)[1]**	**xx (xx.x%)**	**xx (xx.x%)**	**xx (xx.x%)**
**Reason for unblinding – n(%)[2]**			
Consideration for further treatment	xx (xx.x%)	xx (xx.x%)	xx (xx.x%)
Consideration for another trial	xx (xx.x%)	xx (xx.x%)	xx (xx.x%)
Emergency Unblinding	xx (xx.x%)	xx (xx.x%)	xx (xx.x%)
For PV Team - for SAE Reporting	xx (xx.x%)	xx (xx.x%)	xx (xx.x%)
Patients request	xx (xx.x%)	xx (xx.x%)	xx (xx.x%)
Progression	xx (xx.x%)[3]	xx (xx.x%)[4]	xx (xx.x%)

[1] Denominator is the number of patients randomised.

[2] Denominator is the number of patients randomised and who were unblinded.

[3] Includes x patient who also requested for unblinding, x patients who were considered for further treatment, and x patients who were considered for another trial.

[4] Includes x patient who also requested for unblinding, x patients who were considered for further treatment.

4.4 Demographic and baseline characteristics

Table 4 Demographic information recorded at Baseline

Characteristic	Nivolumab (n=xxx)	Placebo (n=xxx)	Total (n=xxx)
**No. of patients with a Baseline eCRF available – n(%)[1]**	**xx (xx.x%)**	**xx (xx.x%)**	**xx (xx.x%)**
**Age**time point **(years)[2]**			
Mean	xx.x	xx.x	xx.x
Standard deviation	x.x	x.x	x.x
Range	xx to xx	xx to xx	xx to xx
Missing from eCRF[3]	xx (xx.x%)	xx (xx.x%)	xx (xx.x%)
**Sex – n(%)[2]**			
Male	xx (xx.x%)	xx (xx.x%)	xx (xx.x%)
Female	xx (xx.x%)	xx (xx.x%)	xx (xx.x%)
Missing from eCRF[3]	xx (xx.x%)	xx (xx.x%)	xx (xx.x%)
**ECOG – n(%)[2]**			
0	xx (xx.x%)	xx (xx.x%)	xx (xx.x%)
1	xx (xx.x%)	xx (xx.x%)	xx (xx.x%)
Missing from eCRF[3]	xx (xx.x%)	xx (xx.x%)	xx (xx.x%)
**PD-L1 Status – n(%)[2]**			
Highly positive (≥50%)	xx (xx.x%)	xx (xx.x%)	xx (xx.x%)
Positive (1-49%)	xx (xx.x%)	xx (xx.x%)	xx (xx.x%)
All positive (≥1%)	*xx (xx.x%)*	*xx (xx.x%)*	*xx (xx.x%)*
Negative (<1%)	xx (xx.x%)	xx (xx.x%)	xx (xx.x%)
Missing from eCRF[3]	xx (xx.x%)	xx (xx.x%)	xx (xx.x%)
**Asbestos Exposure – n(%)**			
Yes	xx (xx.x%)	xx (xx.x%)	xx (xx.x%)
No	xx (xx.x%)	xx (xx.x%)	xx (xx.x%)
Missing from eCRF[3]	xx (xx.x%)	xx (xx.x%)	xx (xx.x%)
**Smoking Status – n(%)**			
No Smoker	xx (xx.x%)	xx (xx.x%)	xx (xx.x%)
Ex-Smoker	xx (xx.x%)	xx (xx.x%)	xx (xx.x%)
Missing from eCRF[3]	xx (xx.x%)	xx (xx.x%)	xx (xx.x%)
**Time since historical diagnosis of mesothelioma (months)**			
Median	xxx.x	xxx.x	xxx.x
Quartiles	xxx.x, xxx.x	xxx.x, xxx.x	xxx.x, xxx.x
Range	xxx.x to xxx.x	xxx.x to xxx.x	xxx.x to xxx.x
Missing from eCRF[3]	xx (xx.x%)	xx (xx.x%)	xx (xx.x%)

[1] Denominator is the number of patients randomised.

[2] These statistics or percentages are calculated using the number of patients with non-missing information available at the time the report is produced
[3] This percentage is calculated as the number of patients with this information missing divided by those with Baseline information available at the time the report is produced.

Table 5 Disease Characteristics

Characteristic	Nivolumab (n=xxx)	Placebo (n=xxx)	Total (n=xxx)
**No. of patients with disease characteristic eCRF available – n(%)[1]**	**xx (xx.x%)**	**xx (xx.x%)**	**xx (xx.x%)**
**Epithelioid vs non-epithelioid – n(%)[2]**			
Epithelioid	xx (xx.x%)	xx (xx.x%)	xx (xx.x%)
Non-epithelioid	xx (xx.x%)	xx (xx.x%)	xx (xx.x%)
**Site of mesothelioma – n(%)[2]**			
Pleural	xx (xx.x%)	xx (xx.x%)	xx (xx.x%)
Non-pleural	xx (xx.x%)	xx (xx.x%)	xx (xx.x%)
Missing from eCRF[3]	xx (xx.x%)	xx (xx.x%)	xx (xx.x%)
**Extra-thoracic metastases – n(%)[2]**			
Yes	xx (xx.x%)	xx (xx.x%)	xx (xx.x%)
No	xx (xx.x%)	xx (xx.x%)	xx (xx.x%)
Missing from eCRF[3]	xx (xx.x%)	xx (xx.x%)	xx (xx.x%)
**TNM staging**			
**T stage – n(%)[2]**			
T0	xx (xx.x%)	xx (xx.x%)	xx (xx.x%)
T1	xx (xx.x%)	xx (xx.x%)	xx (xx.x%)
T2	xx (xx.x%)	xx (xx.x%)	xx (xx.x%)
T3	xx (xx.x%)	xx (xx.x%)	xx (xx.x%)
T4	xx (xx.x%)	xx (xx.x%)	xx (xx.x%)
Missing from eCRF[3]	xx (xx.x%)	xx (xx.x%)	xx (xx.x%)
	xx (xx.x%)	xx (xx.x%)	xx (xx.x%)
**N stage – n(%)[2]**			
N0	xx (xx.x%)	xx (xx.x%)	xx (xx.x%)
N1	xx (xx.x%)	xx (xx.x%)	xx (xx.x%)
N2	xx (xx.x%)	xx (xx.x%)	xx (xx.x%)
N3	xx (xx.x%)	xx (xx.x%)	xx (xx.x%)
Missing from eCRF[3]	xx (xx.x%)	xx (xx.x%)	xx (xx.x%)
	xx (xx.x%)	xx (xx.x%)	xx (xx.x%)
**M stage – n(%)[2]**			
M0	xx (xx.x%)	xx (xx.x%)	xx (xx.x%)
M1	xx (xx.x%)	xx (xx.x%)	xx (xx.x%)
Missing from eCRF[3]	xx (xx.x%)	xx (xx.x%)	xx (xx.x%)

Characteristic	Nivolumab (n=xxx)	Placebo (n=xxx)	Total (n=xxx)
**Line of treatment – n(%)[2]**			
2nd line	xx (xx.x%)	xx (xx.x%)	xx (xx.x%)
3rd line	xx (xx.x%)	xx (xx.x%)	xx (xx.x%)
Greater than 3rd line	xx (xx.x%)	xx (xx.x%)	xx (xx.x%)
Missing from eCRF[3]	xx (xx.x%)	xx (xx.x%)	xx (xx.x%)

[1] Denominator is the number of patients randomised.

[2] These statistics or percentages are calculated using the number of patients with non-missing information available at the time the report is produced

[3] This percentage is calculated as the number of patients with this information missing divided by those with Disease Characteristics information available at the time the DMEC report is produced.

4.5 Treatment information

Table 6 Treatment Information

Characteristic	Nivolumab (n=xxx)	Placebo (n=xxx)	Total (n=xxx)
**No. of patients with a treatment information eCRF available – n(%)[1]**	**xx (xx.x%)**	**xx (xx.x%)**	**xx (xx.x%)**
**Total duration of treatment received (minutes)[2]**			
Median	xxx.x	xxx.x	xxx.x
IQR	xxx to xxx	xxx to xxx	xxx to xxx
Missing from CRF – n(%)[3]	xx (xx.x%)	xx (xx.x%)	xx (xx.x%)
**Any dose delay – n(%)[2]**			
Yes	xx (xx.x%)	xx (xx.x%)	xx (xx.x%)
No	xx (xx.x%)	xx (xx.x%)	xx (xx.x%)
Total	xx (xx.x%)	xx (xx.x%)	xx (xx.x%)
Missing from CRF – n(%)[3]	xx (xx.x%)	xx (xx.x%)	xx (xx.x%)
**Any dose interruption – n(%)[2]**			
Yes	xx (xx.x%)	xx (xx.x%)	xx (xx.x%)
No	xx (xx.x%)	xx (xx.x%)	xx (xx.x%)
Total	xx (xx.x%)	xx (xx.x%)	xx (xx.x%)
Missing from CRF – n(%)[3]	xx (xx.x%)	xx (xx.x%)	xx (xx.x%)
**Number of days on treatment[1]**			
Median	xx.x	xx.x	xx.x
IQR	xxx to xxx	xxx to xxx	xxx to xxx
Range	xxx to xxx	xxx to xxx	xxx to xxx
Mean	xx.x	xx.x	xx.x
SD	xx.x	xx.x	xx.x
Missing from CRF – n(%)[3]	xx (xx.x%)	xx (xx.x%)	xx (xx.x%)

[1] Denominator is the number of patients randomised.
[2] These statistics or percentages are calculated using the number of patients with non-missing information available at the time the report is produced
[3] This percentage is calculated as the number of patients with this information missing divided by those with Treatment information available at the time the report is produced.

4.6 Primary Endpoint Analysis

4.6.1 Overall Survival Information

Table 7 Overall survival information, including Cox models

Characteristic	Nivolumab (n=xx)	Placebo (n=xx)	Total (n=xx)
Median follow-up – median (95%CI)*	xx (xx to xx)	xx (xx to xx)	xx (xx to xx)
Number of events observed – n(%)	xx (xx%)	xx (xx%)	xx (xx%)
Median overall survival – median (95% CI)	xx (xx to xx)	xx (xx to xx)	xx (xx to xx)
12-month overall survival – % (95% CI)	xx% (xx% to xx%)	xx% (xx% to xx%)	xx% (xx% to xx%)
Cox's proportional hazards model[1] – unadjusted			
P-value		xx	
Hazard Ratio (95% CI)		xx (xx, xx)	
Cox's proportional hazards model[1,2] – adjusted			
P-value		xx	
Hazard Ratio (95% CI)		xx (xx, xx)	

* Not included if number of events occurred in >60% of participants

[1] Reference category=Placebo:

- A HR<1 favours Nivolumab
- A HR=1 indicates neither favoured.
- A HR>1 favours Placebo

[2] Adjusting for epithelioid vs. non-epithelioid

Overall survival is deemed significant if the p-value is ≤0.04, as per the Fallback procedure.

Figure 2 Kaplan-Meier plot for overall survivial by treatment arm

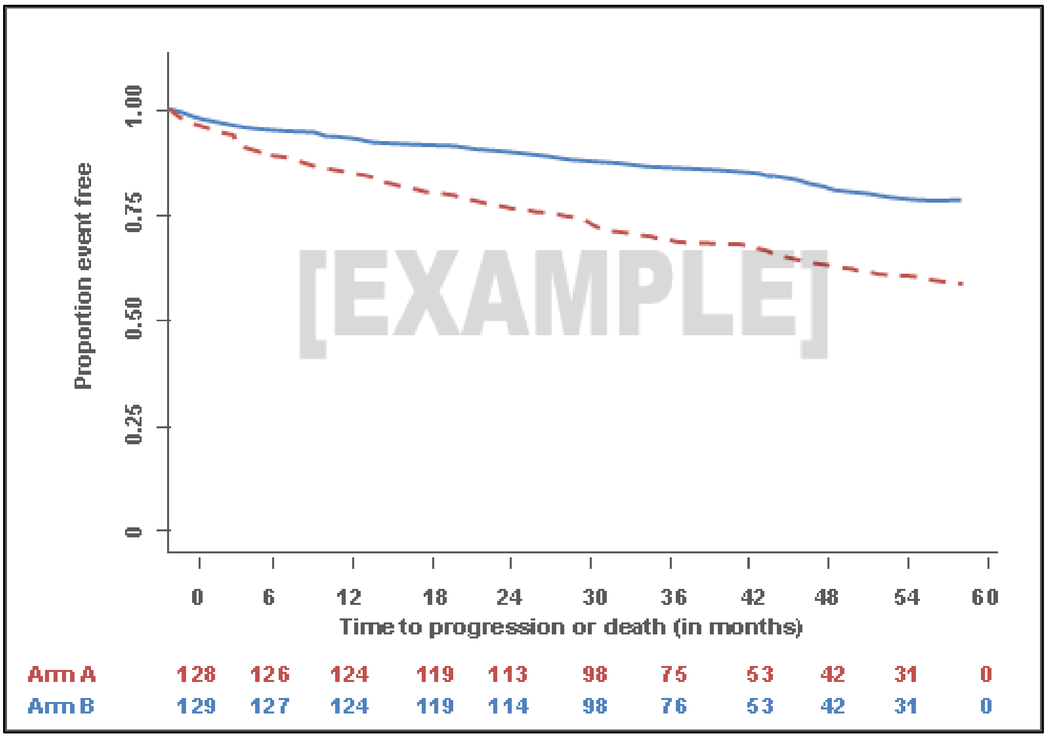

4.6.2 Progression-Free Survival Information

Table 8 Progression-free survival (investigator reported) information, including Cox models

Characteristic	Nivolumab (n=xx)	Placebo (n=xx)	Total (n=xx)
Number of events observed – n(%)	xx (xx%)	xx (xx%)	xx (xx%)
Median progression free survival – median (95% CI)	xx (xx to xx)	xx (xx to xx)	xx (xx to xx)
12-month progression free survival – % (95% CI)	xx% (xx% to xx%)	xx% (xx% to xx%)	xx% (xx% to xx%)
Cox's proportional hazards model[1] – unadjusted			
P-value		xx	
Hazard Ratio (95% CI)		xx (xx, xx)	
Cox's proportional hazards model[1, 2] – adjusted			
P-value		xx	
Hazard Ratio (95% CI)		xx (xx, xx)	

[1] Reference category=Placebo:
- A HR<1 favours Nivolumab
- A HR=1 indicates neither favoured.
- A HR>1 favours Placebo

[2] Adjusting for epithelioid vs. non-epithelioid

Progression free survival is deemed significant if the p-value is ≤0.01 (if OS is not significant) or ≤0.05 (if OS is significant), as per the Fallback procedure.

Figure 3 Kaplan-Meier plot for progresion free survivial (investigator reported) by treatment arm

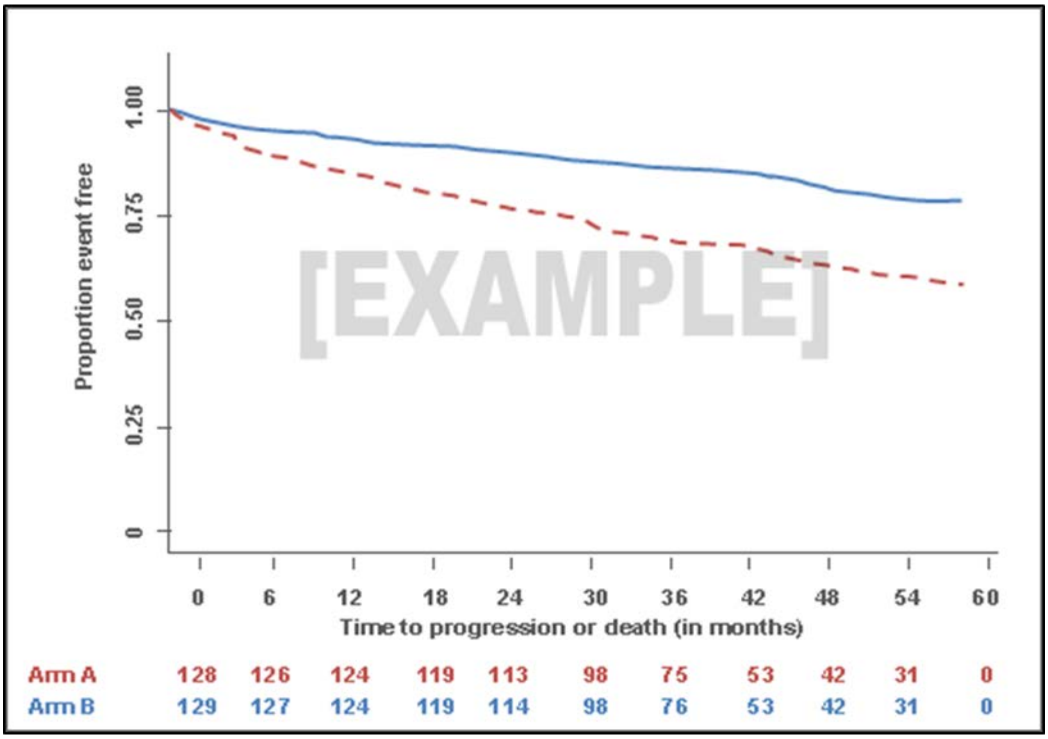

4.7 Other Secondary Endpoint Analysis

Table 9 Progression-free survival (determined by RECIST) information, including Cox models

Characteristic	Nivolumab (n=xx)	Placebo (n=xx)	Total (n=xx)
Number of events observed – n(%)	xx (xx%)	xx (xx%)	xx (xx%)
Median progression free survival – median (95% CI)	xx (xx to xx)	xx (xx to xx)	xx (xx to xx)
12-month progression free survival – % (95% CI)	xx% (xx% to xx%)	xx% (xx% to xx%)	xx% (xx% to xx%)
Cox's proportional hazards model[1] – unadjusted			
P-value		xx	
Hazard Ratio (95% CI)		xx (xx, xx)	
Cox's proportional hazards model[1, 2] – adjusted			
P-value		xx	
Hazard Ratio (95% CI)		xx (xx, xx)	

[1] Reference category=Placebo:

- A HR<1 favours Nivolumab
- A HR=1 indicates neither favoured.
- A HR>1 favours Placebo

[2] Adjusting for epithelioid vs. non-epithelioid

Figure 4 Kaplan-Meier plot for progresion free survivial (determined by RECIST) by treatment arm

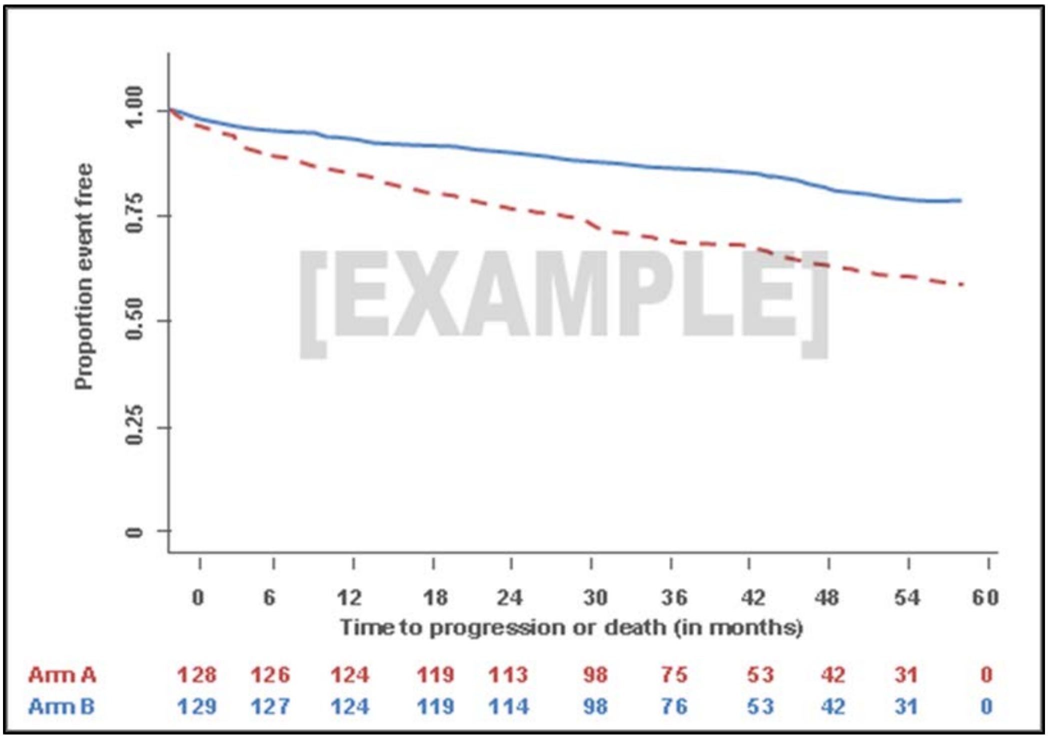

Table 10 Progression data by arm

Characteristic	Nivolumab (n=xx)	Placebo (n=xx)	Total (n=xx)
**Best overall response**[1]			
Complete response	xx (xx%)	xx (xx%)	xx (xx%)
Partial response	xx (xx%)	xx (xx%)	xx (xx%)
Stable disease	xx (xx%)	xx (xx%)	xx (xx%)
Progression	xx (xx%)	xx (xx%)	xx (xx%)

[1]Investigator reported

4.8 Biomarker subgroup Analysis

4.8.1 Overall Survival Information

Table 11 Overall survival information for the PD-L1 subgroup, including Cox models

**Characteristic**	**Nivolumab (n=xxx)**		**Placebo (n=xxx)**		**Total (n=xxx)**	
	**PD-L1 +ve (n=xxx)**	**PD-L1 -ve (n=xxx)**	**PD-L1 +ve (n=xxx)**	**PD-L1 -ve (n=xxx)**	**PD-L1 +ve (n=xxx)**	**PD-L1 -ve (n=xxx)**
Number of events observed – n(%)	xx (xx.x%)	xx (xx.x%)	xx (xx.x%)	xx (xx.x%)	xx (xx.x%)	xx (xx.x%)
Median overall survival – median (95% CI)	xx.x (xx.x to xx.x)	xx.x (xx.x to xx.x)	xx.x (xx.x to xx.x)	xx.x (xx.x to xx.x)	xx.x (xx.x to xx.x)	xx.x (xx.x to xx.x)
12 month overall survival – % (95% CI)	xx.x (xx.x to xx.x)	xx.x (xx.x to xx.x)	xx.x (xx.x to xx.x)	xx.x (xx.x to xx.x)	xx.x (xx.x to xx.x)	xx.x (xx.x to xx.x)

Cox's proportional hazards model including treatment and PD-L1 status interaction[1, 2, 3] – adjusted	
**Treatment**	
P-value	x.xxx
Hazard Ratio (95% CI)	x.xxx (x.xxx, x.xxx)
**PD-L1 status**	
P-value	x.xxx
Hazard Ratio (95% CI)	x.xxx (x.xxx, x.xxx)
**Treatment*PD-L1 status**	
P-value	x.xxx
Hazard Ratio (95% CI)	x.xxx (x.xxx, x.xxx)

[1] Reference category=Placebo
[2] Reference category=Negative PD-L1 status
[3] Adjusted for epithelioid status and line of treatment

Table 12 Overall survival information for the PD-L1 subgroup, including Cox models

Characteristic	Nivolumab (n=xxx)			Placebo (n=xxx)			Total (n=xxx)		
	PD-L1 high +ve (n=xxx)	PD-L1 low +ve (n=xxx)	PD-L1 -ve (n=xxx)	PD-L1 high +ve (n=xxx)	PD-L1 low +ve (n=xxx)	PD-L1 -ve (n=xxx)	PD-L1 high +ve (n=xxx)	PD-L1 low +ve (n=xxx)	PD-L1 -ve (n=xxx)
Number of events observed – n(%)	xx (xx.x%)	xx (xx.x%)	xx (xx.x%)	xx (xx.x%)	xx (xx.x%)	xx (xx.x%)	xx (xx.x%)	xx (xx.x%)	xx (xx.x%)
Median overall survival – median (95% CI)	xx.x (xx.x to xx.x)	xx.x (xx.x to xx.x)	xx.x (xx.x to xx.x)	xx.x (xx.x to xx.x)	xx.x (xx.x to xx.x)	xx.x (xx.x to xx.x)	xx.x (xx.x to xx.x)	xx.x (xx.x to xx.x)	xx.x (xx.x to xx.x)
12 month overall survival – % (95% CI)	xx.x (xx.x to xx.x)	xx.x (xx.x to xx.x)	xx.x (xx.x to xx.x)	xx.x (xx.x to xx.x)	xx.x (xx.x to xx.x)	xx.x (xx.x to xx.x)	xx.x (xx.x to xx.x)	xx.x (xx.x to xx.x)	xx.x (xx.x to xx.x)

Cox's proportional hazards model including treatment and PD-L1 status interaction[1, 2, 3] – adjusted	
**Treatment**	
P-value	x.xxx
Hazard Ratio (95% CI)	x.xxx (x.xxx, x.xxx)
**PD-L1 high +ve**	
P-value	x.xxx
Hazard Ratio (95% CI)	x.xxx (x.xxx, x.xxx)
**PD-L1 low +ve**	
P-value	x.xxx
Hazard Ratio (95% CI)	x.xxx (x.xxx, x.xxx)
**Treatment*PD-L1 high +ve**	
P-value	x.xxx
Hazard Ratio (95% CI)	x.xxx (x.xxx, x.xxx)
**Treatment*PD-L1 low +ve**	
P-value	x.xxx
Hazard Ratio (95% CI)	x.xxx (x.xxx, x.xxx)

[1] Reference category=Placebo
[2] Reference category=Negative PD-L1 status
[3] Adjusted for epithelioid status and line of treatment

Figure 4 Kaplan-Meier plot for overall survivial by treatment arm and PD-L1 status

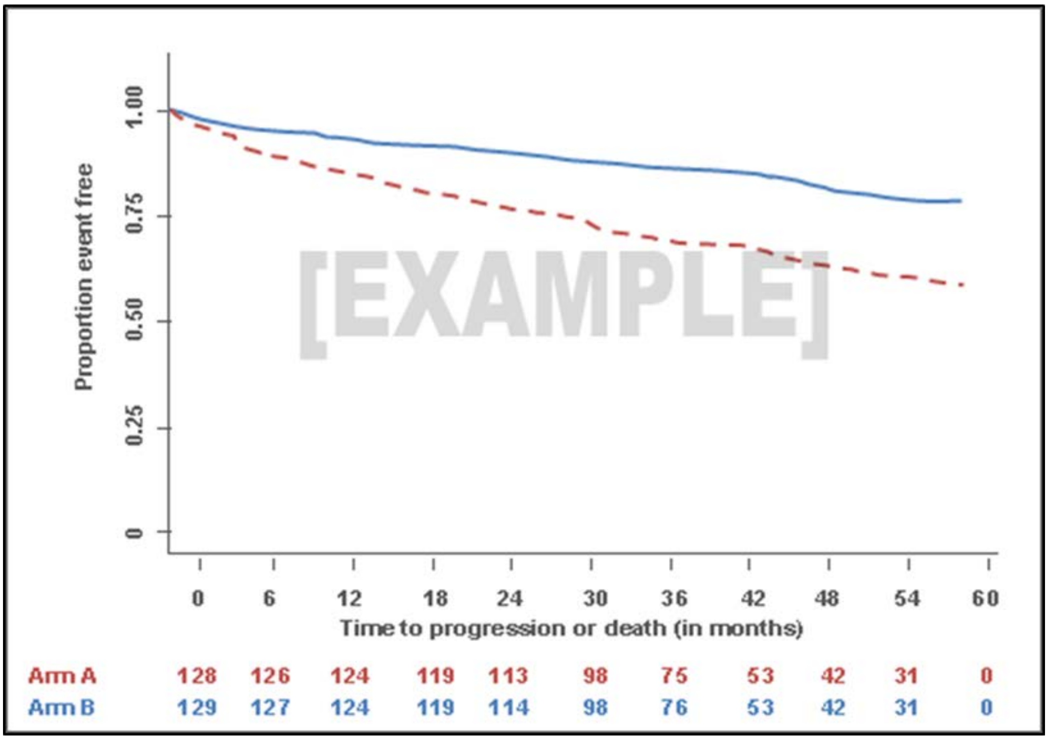

4.8.2 Progression Free Survival Information

Table 13 Progression free survival information for the PD-L1 subgroup, including Cox models

Characteristic	Nivolumab (n=xxx)		Placebo (n=xxx)		Total (n=xxx)	
	**PD-L1 +ve (n=xxx)**	**PD-L1 -ve (n=xxx)**	**PD-L1 +ve (n=xxx)**	**PD-L1 -ve (n=xxx)**	**PD-L1 +ve (n=xxx)**	**PD-L1 -ve (n=xxx)**
Number of events observed – n(%)	xx (xx.x%)	xx (xx.x%)	xx (xx.x%)	xx (xx.x%)	xx (xx.x%)	xx (xx.x%)
Median overall survival – median (95% CI)	xx.x (xx.x to xx.x)	xx.x (xx.x to xx.x)	xx.x (xx.x to xx.x)	xx.x (xx.x to xx.x)	xx.x (xx.x to xx.x)	xx.x (xx.x to xx.x)
12 month overall survival – % (95% CI)	xx.x (xx.x to xx.x)	xx.x (xx.x to xx.x)	xx.x (xx.x to xx.x)	xx.x (xx.x to xx.x)	xx.x (xx.x to xx.x)	xx.x (xx.x to xx.x)
Cox's proportional hazards model including treatment and PD-L1 status interaction[1, 2, 3] – adjusted						
**Treatment**						
P-value	x.xxx					
Hazard Ratio (95% CI)	x.xxx (x.xxx, x.xxx)					
**PD-L1 status**						
P-value	x.xxx					
Hazard Ratio (95% CI)	x.xxx (x.xxx, x.xxx)					
**Treatment*PD-L1 status**						
P-value	x.xxx					
Hazard Ratio (95% CI)	x.xxx (x.xxx, x.xxx)					

[1] Reference category=Placebo
[2] Reference category=Negative PD-L1 status
[3] Adjusted for epithelioid status and line of treatment

Table 14 Progression free survival information for the PD-L1 subgroup, including Cox models

Characteristic	Nivolumab (n=xxx)			Placebo (n=xxx)			Total (n=xxx)		
	PD-L1 high +ve (n=xxx)	PD-L1 low +ve (n=xxx)	PD-L1 -ve (n=xxx)	PD-L1 high +ve (n=xxx)	PD-L1 low +ve (n=xxx)	PD-L1 -ve (n=xxx)	PD-L1 high +ve (n=xxx)	PD-L1 low +ve (n=xxx)	PD-L1 -ve (n=xxx)
Number of events observed – n(%)	xx (xx.x%)	xx (xx.x%)	xx (xx.x%)	xx (xx.x%)	xx (xx.x%)	xx (xx.x%)	xx (xx.x%)	xx (xx.x%)	xx (xx.x%)
Median overall survival – median (95% CI)	xx.x (xx.x to xx.x)	xx.x (xx.x to xx.x)	xx.x (xx.x to xx.x)	xx.x (xx.x to xx.x)	xx.x (xx.x to xx.x)	xx.x (xx.x to xx.x)	xx.x (xx.x to xx.x)	xx.x (xx.x to xx.x)	xx.x (xx.x to xx.x)
12 month overall survival – % (95% CI)	xx.x (xx.x to xx.x)	xx.x (xx.x to xx.x)	xx.x (xx.x to xx.x)	xx.x (xx.x to xx.x)	xx.x (xx.x to xx.x)	xx.x (xx.x to xx.x)	xx.x (xx.x to xx.x)	xx.x (xx.x to xx.x)	xx.x (xx.x to xx.x)

Cox's proportional hazards model including treatment and PD-L1 status interaction[1, 2, 3] – adjusted	
**Treatment**	
P-value	x.xxx
Hazard Ratio (95% CI)	x.xxx (x.xxx, x.xxx)
**PD-L1 high +ve**	
P-value	x.xxx
Hazard Ratio (95% CI)	x.xxx (x.xxx, x.xxx)
**PD-L1 low +ve**	
P-value	x.xxx
Hazard Ratio (95% CI)	x.xxx (x.xxx, x.xxx)
**Treatment*PD-L1 high +ve**	
P-value	x.xxx
Hazard Ratio (95% CI)	x.xxx (x.xxx, x.xxx)
**Treatment*PD-L1 low +ve**	
P-value	x.xxx
Hazard Ratio (95% CI)	x.xxx (x.xxx, x.xxx)

[1] Reference category=Placebo
[2] Reference category=Negative PD-L1 status
[3] Adjusted for epithelioid status and line of treatment

Figure 5 Kaplan-Meier plot for progression free survivial by treatment arm and PD-L1 status

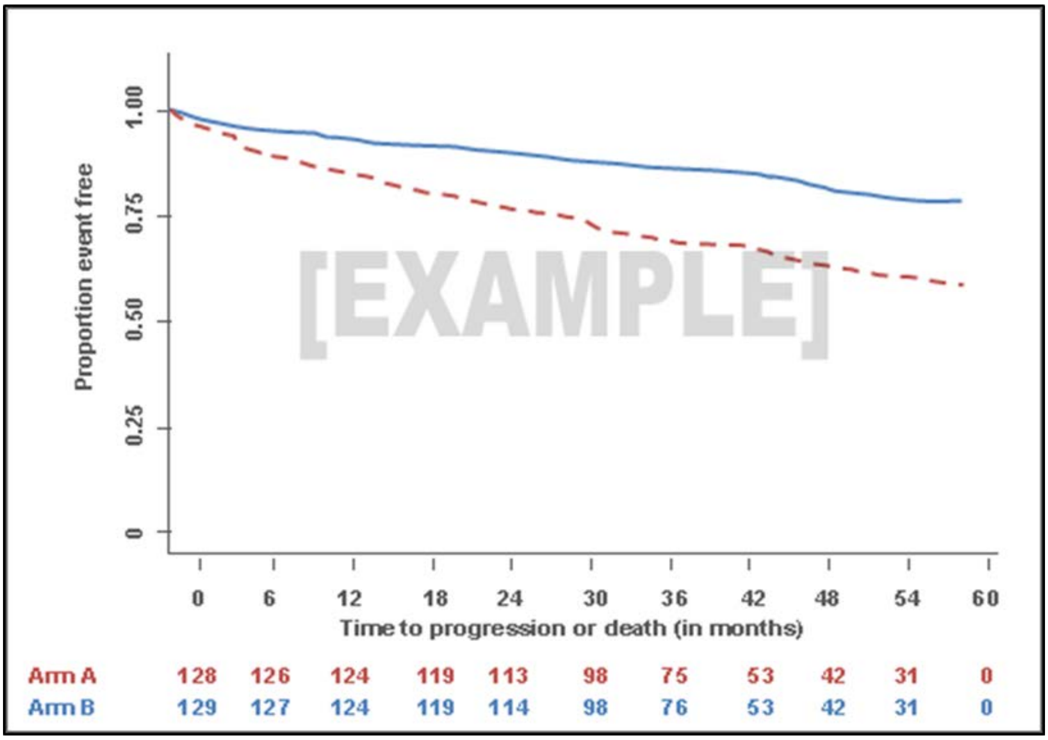

Table 15 Progression data by arm

Characteristic	Nivolumab		Placebo		Total	
	(n=xx)	(n=xx)	(n=xx)	(n=xx)	(n=xx)	(n=xx)
**Best overall response[1]**						
Complete response	xx (xx%)	xx (xx%)	xx (xx%)	xx (xx%)	xx (xx%)	xx (xx%)
Partial response	xx (xx%)	xx (xx%)	xx (xx%)	xx (xx%)	xx (xx%)	xx (xx%)
Stable disease	xx (xx%)	xx (xx%)	xx (xx%)	xx (xx%)	xx (xx%)	xx (xx%)
Progression	xx (xx%)	xx (xx%)	xx (xx%)	xx (xx%)	xx (xx%)	xx (xx%)

[1]Investigator response

Figure 6 Forest plot

A forest plot will be used to display treatment effect (based on hazard ratios and confidence intervals from univariable Cox proportional hazards model) across the following variables:

- Age (<75 years, or 75 years and older)
- Sex
- Asbestos exposure
- Histology (epithelioid or non-epithelioid)
- Line of therapy
- Smoking status
- Site of mesothelioma

- Extra-thoracic metastases
- ECOG
- PD-L1 status (high positive, low positive, negative)

4.9 Safety reporting

4.9.1 Toxicity

Table 16 Overall toxicity by CTCAE Grade

Characteristic	Nivolumab (n=xxx)	Placebo (n=xxx)	Total (n=xxx)
**Adverse events – n(%)[1,2]**			
CTCAE 4.0 Grade 1	xx (xx.x%)	xx (xx.x%)	xx (xx.x%)
CTCAE 4.0 Grade 2	xx (xx.x%)	xx (xx.x%)	xx (xx.x%)
CTCAE 4.0 Grade 3	xx (xx.x%)	xx (xx.x%)	xx (xx.x%)
CTCAE 4.0 Grade 4	xx (xx.x%)	xx (xx.x%)	xx (xx.x%)
CTCAE 4.0 Grade 5	xx (xx.x%)	xx (xx.x%)	xx (xx.x%)
Reported AE with Missing Grade – n(%)	xx (xx.x%)	xx (xx.x%)	xx (xx.x%)
No AE	xx (xx.x%)	xx (xx.x%)	xx (xx.x%)
Severe (CTCAE v4.0 Grade 3 or above) adverse events – n(%)[1,2]	xx (xx.x%)	xx (xx.x%)	xx (xx.x%)

[1] Denominator is the number of patients randomised.
[2] The worst grade is used when more than one grade is available for a patient.

Table 17 Overall Toxicity

Characteristic	Nivolumab (n=xxx)	Placebo (n=xxx)	Total (n=xxx)
**Number of patients experiencing at least one AE – n(%)[1]**	**xx (xx.x%)**	**xx (xx.x%)**	**xx (xx.x%)**
**Summary of AEs – n(%)[1, 2]**			
**Blood and lymphatic system disorders**	**xx (xx.x%)**	**xx (xx.x%)**	**xx (xx.x%)**
**… [*]**	xx (xx.x%)	xx (xx.x%)	xx (xx.x%)
**Cardiac disorders**	**xx (xx.x%)**	**xx (xx.x%)**	**xx (xx.x%)**
**…**	xx (xx.x%)	xx (xx.x%)	xx (xx.x%)
**Ear and labyrinth disorders**	**xx (xx.x%)**	**xx (xx.x%)**	**xx (xx.x%)**
**…**	xx (xx.x%)	xx (xx.x%)	xx (xx.x%)
**Endocrine disorders**	**xx (xx.x%)**	**xx (xx.x%)**	**xx (xx.x%)**
**…**	xx (xx.x%)	xx (xx.x%)	xx (xx.x%)
**Eye disorders**	**xx (xx.x%)**	**xx (xx.x%)**	**xx (xx.x%)**
**…**	xx (xx.x%)	xx (xx.x%)	xx (xx.x%)
**Gastrointestinal disorders**	**xx (xx.x%)**	**xx (xx.x%)**	**xx (xx.x%)**
**…**	xx (xx.x%)	xx (xx.x%)	xx (xx.x%)

**General disorders and administration site conditions**	**xx (xx.x%)**	**xx (xx.x%)**	**xx (xx.x%)**
…	xx (xx.x%)	xx (xx.x%)	xx (xx.x%)
**Hepatobiliary disorders**	**xx (xx.x%)**	**xx (xx.x%)**	**xx (xx.x%)**
…	xx (xx.x%)	xx (xx.x%)	xx (xx.x%)
**Immune system disorders**	**xx (xx.x%)**	**xx (xx.x%)**	**xx (xx.x%)**
…	xx (xx.x%)	xx (xx.x%)	xx (xx.x%)
**Infections and infestations**	**xx (xx.x%)**	**xx (xx.x%)**	**xx (xx.x%)**
…	xx (xx.x%)	xx (xx.x%)	xx (xx.x%)
**Injury, poisoning and procedural complications**	**xx (xx.x%)**	**xx (xx.x%)**	**xx (xx.x%)**
…	xx (xx.x%)	xx (xx.x%)	xx (xx.x%)
**Investigations**	**xx (xx.x%)**	**xx (xx.x%)**	**xx (xx.x%)**
…	xx (xx.x%)	xx (xx.x%)	xx (xx.x%)
**Metabolism and nutrition disorders**	**xx (xx.x%)**	**xx (xx.x%)**	**xx (xx.x%)**
…	xx (xx.x%)	xx (xx.x%)	xx (xx.x%)
**Musculoskeletal and connective tissue disorders**	**xx (xx.x%)**	**xx (xx.x%)**	**xx (xx.x%)**
…	xx (xx.x%)	xx (xx.x%)	xx (xx.x%)
**Neoplasms benign, malignant and unspecified (incl cysts and polyps)**	**xx (xx.x%)**	**xx (xx.x%)**	**xx (xx.x%)**
…	xx (xx.x%)	xx (xx.x%)	xx (xx.x%)
**Nervous system disorders**	**xx (xx.x%)**	**xx (xx.x%)**	**xx (xx.x%)**
…	xx (xx.x%)	xx (xx.x%)	xx (xx.x%)
**Psychiatric disorders**	**xx (xx.x%)**	**xx (xx.x%)**	**xx (xx.x%)**
…	xx (xx.x%)	xx (xx.x%)	xx (xx.x%)
**Renal and urinary disorders**	**xx (xx.x%)**	**xx (xx.x%)**	**xx (xx.x%)**
…	xx (xx.x%)	xx (xx.x%)	xx (xx.x%)
**Reproductive system and breast disorders**	**xx (xx.x%)**	**xx (xx.x%)**	**xx (xx.x%)**
…	xx (xx.x%)	xx (xx.x%)	xx (xx.x%)
**Respiratory, thoracic and mediastinal disorders**	**xx (xx.x%)**	**xx (xx.x%)**	**xx (xx.x%)**
…	xx (xx.x%)	xx (xx.x%)	xx (xx.x%)
**Skin and subcutaneous tissue disorders**	**xx (xx.x%)**	**xx (xx.x%)**	**xx (xx.x%)**
…	xx (xx.x%)	xx (xx.x%)	xx (xx.x%)

**Surgical and medical procedures**<br>…	**xx (xx.x%)**<br>xx (xx.x%)	**xx (xx.x%)**<br>xx (xx.x%)	**xx (xx.x%)**<br>xx (xx.x%)
**Vascular disorders**<br>…	**xx (xx.x%)**<br>xx (xx.x%)	**xx (xx.x%)**<br>xx (xx.x%)	**xx (xx.x%)**<br>xx (xx.x%)

[1] Denominator is the number of patients randomised.
[2] The worst grade is used when more than one grade is available for a patient.
[*] This data will be preferred terms within the system organ class.

Table 18 Overall Toxicity (Grade 3 or above)

Characteristic	Nivolumab (n=xxx)	Placebo (n=xxx)	Total (n=xxx)
**Number of patients that experienced at least one grade 3 or above AE – n(%)[1]**	**xx (xx.x%)**	**xx (xx.x%)**	**xx (xx.x%)**
**Summary of AEs – n(%)[1, 2]**			
**Blood and lymphatic system disorders**<br>…	**xx (xx.x%)**<br>xx (xx.x%)	**xx (xx.x%)**<br>xx (xx.x%)	**xx (xx.x%)**<br>xx (xx.x%)
**Cardiac disorders**<br>…	**xx (xx.x%)**<br>xx (xx.x%)	**xx (xx.x%)**<br>xx (xx.x%)	**xx (xx.x%)**<br>xx (xx.x%)
**Ear and labyrinth disorders**<br>…	**xx (xx.x%)**<br>xx (xx.x%)	**xx (xx.x%)**<br>xx (xx.x%)	**xx (xx.x%)**<br>xx (xx.x%)
**Endocrine disorders**<br>…	**xx (xx.x%)**<br>xx (xx.x%)	**xx (xx.x%)**<br>xx (xx.x%)	**xx (xx.x%)**<br>xx (xx.x%)
**Eye disorders**<br>…	**xx (xx.x%)**<br>xx (xx.x%)	**xx (xx.x%)**<br>xx (xx.x%)	**xx (xx.x%)**<br>xx (xx.x%)
**Gastrointestinal disorders**<br>…	**xx (xx.x%)**<br>xx (xx.x%)	**xx (xx.x%)**<br>xx (xx.x%)	**xx (xx.x%)**<br>xx (xx.x%)
**General disorders and administration site conditions**<br>…	**xx (xx.x%)**<br>xx (xx.x%)	**xx (xx.x%)**<br>xx (xx.x%)	**xx (xx.x%)**<br>xx (xx.x%)
**Hepatobiliary disorders**<br>…	**xx (xx.x%)**<br>xx (xx.x%)	**xx (xx.x%)**<br>xx (xx.x%)	**xx (xx.x%)**<br>xx (xx.x%)
**Immune system disorders**<br>…	**xx (xx.x%)**<br>xx (xx.x%)	**xx (xx.x%)**<br>xx (xx.x%)	**xx (xx.x%)**<br>xx (xx.x%)
**Infections and infestations**<br>…	**xx (xx.x%)**<br>xx (xx.x%)	**xx (xx.x%)**<br>xx (xx.x%)	**xx (xx.x%)**<br>xx (xx.x%)
**Injury, poisoning and procedural complications**	**xx (xx.x%)**	**xx (xx.x%)**	**xx (xx.x%)**

…	xx (xx.x%)	xx (xx.x%)	xx (xx.x%)
**Investigations**	**xx (xx.x%)**	**xx (xx.x%)**	**xx (xx.x%)**
…	xx (xx.x%)	xx (xx.x%)	xx (xx.x%)
**Metabolism and nutrition disorders**	**xx (xx.x%)**	**xx (xx.x%)**	**xx (xx.x%)**
…	xx (xx.x%)	xx (xx.x%)	xx (xx.x%)
**Musculoskeletal and connective tissue disorders**	**xx (xx.x%)**	**xx (xx.x%)**	**xx (xx.x%)**
…	xx (xx.x%)	xx (xx.x%)	xx (xx.x%)
**Neoplasms benign, malignant and unspecified (incl cysts and polyps)**	**xx (xx.x%)**	**xx (xx.x%)**	**xx (xx.x%)**
…	xx (xx.x%)	xx (xx.x%)	xx (xx.x%)
**Nervous system disorders**	**xx (xx.x%)**	**xx (xx.x%)**	**xx (xx.x%)**
…	xx (xx.x%)	xx (xx.x%)	xx (xx.x%)
**Psychiatric disorders**	**xx (xx.x%)**	**xx (xx.x%)**	**xx (xx.x%)**
…	xx (xx.x%)	xx (xx.x%)	xx (xx.x%)
**Renal and urinary disorders**	**xx (xx.x%)**	**xx (xx.x%)**	**xx (xx.x%)**
…	xx (xx.x%)	xx (xx.x%)	xx (xx.x%)
**Reproductive system and breast disorders**	**xx (xx.x%)**	**xx (xx.x%)**	**xx (xx.x%)**
…	xx (xx.x%)	xx (xx.x%)	xx (xx.x%)
**Respiratory, thoracic and mediastinal disorders**	**xx (xx.x%)**	**xx (xx.x%)**	**xx (xx.x%)**
…	xx (xx.x%)	xx (xx.x%)	xx (xx.x%)
**Skin and subcutaneous tissue disorders**	**xx (xx.x%)**	**xx (xx.x%)**	**xx (xx.x%)**
…	xx (xx.x%)	xx (xx.x%)	xx (xx.x%)
**Surgical and medical procedures**	**xx (xx.x%)**	**xx (xx.x%)**	**xx (xx.x%)**
…	xx (xx.x%)	xx (xx.x%)	xx (xx.x%)
**Vascular disorders**	**xx (xx.x%)**	**xx (xx.x%)**	**xx (xx.x%)**
…	xx (xx.x%)	xx (xx.x%)	xx (xx.x%)

[1] Denominator is the number of patients randomised.
[2] The worst grade is used when more than one grade is available for a patient.

any grade 5 AEs will be listed in the footnote

4.9.2 Reported Serious Adverse Events (SAEs)/Serious Adverse Reactions (SARs) and Suspected Unexpected Serious Adverse Reactions (SUSARs)

There have been xxx SAEs/SARs/SUSARs reported. A summary of these are shown below together with a full list of each event reported sorted by Principal Investigator (PI) assessment, patient and date.

Table 19 Summary of the SAEs reported

Characteristic	Nivolumab (n=xxx)	Placebo (n=xxx)	Total (n=xxx)
Number of patients experiencing at least one SAE/SAR/SUSAR – n(%)[1]	xx (xx.x%)	xx (xx.x%)	xx (xx.x%)
Number of SAE/SAR/SUSAR per patient (for patients with at least one SAE/SAR/SUSAR) – median (range)	x (x.x to x.x)	x (x.x to x.x)	x (x.x to x.x)
**PI assessment – n(%)[2]**			
SUSAR (Suspected Unexpected Serious Adverse Reaction)	xx (xx.x%)	xx (xx.x%)	xx (xx.x%)
SAR (Serious Adverse Reaction)	xx (xx.x%)	xx (xx.x%)	xx (xx.x%)
SAE (Serious Adverse Event)	xx (xx.x%)	xx (xx.x%)	xx (xx.x%)
Pending SUSAR	xx (xx.x%)	xx (xx.x%)	xx (xx.x%)
Total	xx (xx.x%)	xx (xx.x%)	xx (xx.x%)
**CTCAE v4.0 grade – n(%)[2]**			
1 – Mild	xx (xx.x%)	xx (xx.x%)	xx (xx.x%)
2 – Moderate	xx (xx.x%)	xx (xx.x%)	xx (xx.x%)
3 – Severe	xx (xx.x%)	xx (xx.x%)	xx (xx.x%)
4 – Life threatening	xx (xx.x%)	xx (xx.x%)	xx (xx.x%)
5 – Death related to AE	xx (xx.x%)	xx (xx.x%)	xx (xx.x%)
Missing	xx (xx.x%)	xx (xx.x%)	xx (xx.x%)
Total	xx (xx.x%)	xx (xx.x%)	xx (xx.x%)
**Why was the event serious – n(%)[2]**			
1 – Resulted in death	xx (xx.x%)	xx (xx.x%)	xx (xx.x%)
2 – Life threatening	xx (xx.x%)	xx (xx.x%)	xx (xx.x%)
3 – Required hospitalisation or prolongation of existing hospitalisation	xx (xx.x%)	xx (xx.x%)	xx (xx.x%)
6 – Other important medical event	xx (xx.x%)	xx (xx.x%)	xx (xx.x%)
Missing	xx (xx.x%)	xx (xx.x%)	xx (xx.x%)
Total	xx (xx.x%)	xx (xx.x%)	xx (xx.x%)

[1] Denominator is the number of patients randomised.

[2] Denominator is the number of SAEs/SARs/SUSARs with non-missing information.

[3] Denominator is the number of SAEs/SARs/SUSAR.

Table 20 Summary of the main symptom(s) reported on the SAE form

Characteristic	Nivolumab (n=xxx)	Placebo (n=xxx)	Total (n=xxx)
**Number of patients experiencing at least one SAE/SAR/SUSAR – n(%)[1]**	**xx (xx.x%)**	**xx (xx.x%)**	**xx (xx.x%)**
**Summary of SAEs – n(%)[1, 2]**			
**Blood and lymphatic system disorders**	**xx (xx.x%)**	**xx (xx.x%)**	**xx (xx.x%)**
…	xx (xx.x%)	xx (xx.x%)	xx (xx.x%)
**Cardiac disorders**	**xx (xx.x%)**	**xx (xx.x%)**	**xx (xx.x%)**
…	xx (xx.x%)	xx (xx.x%)	xx (xx.x%)
**Endocrine disorders**	**xx (xx.x%)**	**xx (xx.x%)**	**xx (xx.x%)**
…	xx (xx.x%)	xx (xx.x%)	xx (xx.x%)
**Eye disorders**	**xx (xx.x%)**	**xx (xx.x%)**	**xx (xx.x%)**
…	xx (xx.x%)	xx (xx.x%)	xx (xx.x%)
**Gastrointestinal disorders**	**xx (xx.x%)**	**xx (xx.x%)**	**xx (xx.x%)**
…	xx (xx.x%)	xx (xx.x%)	xx (xx.x%)
**General disorders and administration site conditions**	**xx (xx.x%)**	**xx (xx.x%)**	**xx (xx.x%)**
…	xx (xx.x%)	xx (xx.x%)	xx (xx.x%)
**Hepatobiliary disorders**	**xx (xx.x%)**	**xx (xx.x%)**	**xx (xx.x%)**
…	xx (xx.x%)	xx (xx.x%)	xx (xx.x%)
**Infections and infestations**	**xx (xx.x%)**	**xx (xx.x%)**	**xx (xx.x%)**
…	xx (xx.x%)	xx (xx.x%)	xx (xx.x%)
**Injury, poisoning and procedural complications**	**xx (xx.x%)**	**xx (xx.x%)**	**xx (xx.x%)**
…	xx (xx.x%)	xx (xx.x%)	xx (xx.x%)
**Investigations**	**xx (xx.x%)**	**xx (xx.x%)**	**xx (xx.x%)**
…	xx (xx.x%)	xx (xx.x%)	xx (xx.x%)
**Metabolism and nutrition disorders**	**xx (xx.x%)**	**xx (xx.x%)**	**xx (xx.x%)**
…	xx (xx.x%)	xx (xx.x%)	xx (xx.x%)
**Musculoskeletal and connective tissue disorders**	**xx (xx.x%)**	**xx (xx.x%)**	**xx (xx.x%)**
…	xx (xx.x%)	xx (xx.x%)	xx (xx.x%)
**Neoplasms benign, malignant and unspecified (incl cysts and polyps)**	**xx (xx.x%)**	**xx (xx.x%)**	**xx (xx.x%)**
…	xx (xx.x%)	xx (xx.x%)	xx (xx.x%)

**Nervous system disorders**	**xx (xx.x%)**	**xx (xx.x%)**	**xx (xx.x%)**
…	xx (xx.x%)	xx (xx.x%)	xx (xx.x%)
**Psychiatric disorders**	**xx (xx.x%)**	**xx (xx.x%)**	**xx (xx.x%)**
…	xx (xx.x%)	xx (xx.x%)	xx (xx.x%)
**Renal and urinary disorders**	**xx (xx.x%)**	**xx (xx.x%)**	**xx (xx.x%)**
…	xx (xx.x%)	xx (xx.x%)	xx (xx.x%)
**Reproductive system and breast disorders**	**xx (xx.x%)**	**xx (xx.x%)**	**xx (xx.x%)**
…	xx (xx.x%)	xx (xx.x%)	xx (xx.x%)
**Respiratory, thoracic and mediastinal disorders**	**xx (xx.x%)**	**xx (xx.x%)**	**xx (xx.x%)**
…	xx (xx.x%)	xx (xx.x%)	xx (xx.x%)
**Surgical and medical procedures**	**xx (xx.x%)**	**xx (xx.x%)**	**xx (xx.x%)**
…	xx (xx.x%)	xx (xx.x%)	xx (xx.x%)
**Vascular Disorders**	**xx (xx.x%)**	**xx (xx.x%)**	**xx (xx.x%)**
…	xx (xx.x%)	xx (xx.x%)	xx (xx.x%)

[1] Denominator is the number of patients randomised.
[2] The worst grade is used when more than one grade is available for a patient

Table 21 Summary of the main symptom(s) reported on the SAE form (Grade 3 or above)

Characteristic	Nivolumab (n=xxx)	Placebo (n=xxx)	Total (n=xxx)
**Number of patients that experienced at least one grade 3 or above SAE – n(%)[1]**	**xx (xx.x%)**	**xx (xx.x%)**	**xx (xx.x%)**
**Summary of SAEs – n(%)[1, 2]**			
**Blood and lymphatic system disorders**	**xx (xx.x%)**	**xx (xx.x%)**	**xx (xx.x%)**
…	xx (xx.x%)	xx (xx.x%)	xx (xx.x%)
**Cardiac disorders**	**xx (xx.x%)**	**xx (xx.x%)**	**xx (xx.x%)**
…	xx (xx.x%)	xx (xx.x%)	xx (xx.x%)
**Endocrine disorders**	**xx (xx.x%)**	**xx (xx.x%)**	**xx (xx.x%)**
…	xx (xx.x%)	xx (xx.x%)	xx (xx.x%)
**Eye disorders**	**xx (xx.x%)**	**xx (xx.x%)**	**xx (xx.x%)**
…	xx (xx.x%)	xx (xx.x%)	xx (xx.x%)
**Gastrointestinal disorders**	**xx (xx.x%)**	**xx (xx.x%)**	**xx (xx.x%)**
…	xx (xx.x%)	xx (xx.x%)	xx (xx.x%)
**General disorders and administration site conditions**	**xx (xx.x%)**	**xx (xx.x%)**	**xx (xx.x%)**
…	xx (xx.x%)	xx (xx.x%)	xx (xx.x%)
**Hepatobiliary disorders**	**xx (xx.x%)**	**xx (xx.x%)**	**xx (xx.x%)**
…	xx (xx.x%)	xx (xx.x%)	xx (xx.x%)
**Infections and infestations**	**xx (xx.x%)**	**xx (xx.x%)**	**xx (xx.x%)**
…	xx (xx.x%)	xx (xx.x%)	xx (xx.x%)
**Injury, poisoning and procedural complications**	**xx (xx.x%)**	**xx (xx.x%)**	**xx (xx.x%)**
…	xx (xx.x%)	xx (xx.x%)	xx (xx.x%)
**Investigations**	**xx (xx.x%)**	**xx (xx.x%)**	**xx (xx.x%)**
…	xx (xx.x%)	xx (xx.x%)	xx (xx.x%)
**Metabolism and nutrition disorders**	**xx (xx.x%)**	**xx (xx.x%)**	**xx (xx.x%)**
…	xx (xx.x%)	xx (xx.x%)	xx (xx.x%)
**Musculoskeletal and connective tissue disorders**	**xx (xx.x%)**	**xx (xx.x%)**	**xx (xx.x%)**
…	xx (xx.x%)	xx (xx.x%)	xx (xx.x%)
**Neoplasms benign, malignant and unspecified (incl cysts and polyps)**	**xx (xx.x%)**	**xx (xx.x%)**	**xx (xx.x%)**
…	xx (xx.x%)	xx (xx.x%)	xx (xx.x%)

**Nervous system disorders**	**xx (xx.x%)**	**xx (xx.x%)**	**xx (xx.x%)**
…	xx (xx.x%)	xx (xx.x%)	xx (xx.x%)
**Psychiatric disorders**	**xx (xx.x%)**	**xx (xx.x%)**	**xx (xx.x%)**
…	xx (xx.x%)	xx (xx.x%)	xx (xx.x%)
**Renal and urinary disorders**	**xx (xx.x%)**	**xx (xx.x%)**	**xx (xx.x%)**
…	xx (xx.x%)	xx (xx.x%)	xx (xx.x%)
**Reproductive system and breast disorders**	**xx (xx.x%)**	**xx (xx.x%)**	**xx (xx.x%)**
…	xx (xx.x%)	xx (xx.x%)	xx (xx.x%)
**Respiratory, thoracic and mediastinal disorders**	**xx (xx.x%)**	**xx (xx.x%)**	**xx (xx.x%)**
…	xx (xx.x%)	xx (xx.x%)	xx (xx.x%)
**Surgical and medical procedures**	**xx (xx.x%)**	**xx (xx.x%)**	**xx (xx.x%)**
…	xx (xx.x%)	xx (xx.x%)	xx (xx.x%)
**Vascular Disorders**	**xx (xx.x%)**	**xx (xx.x%)**	**xx (xx.x%)**
…	xx (xx.x%)	xx (xx.x%)	xx (xx.x%)

[1] Denominator is the number of patients randomised.
[2] The worst grade is used when more than one grade is available for a patient.

References

[Provide a list of references used in the SAP, including reference to the Data Management Plan and Statistical Master File]

5 SAP revision history

Version number	Revision history	Author	Date
0.1	First draft – based on v7 of the protocol	Sam Wilding	07-SEPT-2020
0.2	Updates following review by Kayleigh Hill	Kayleigh Hill	08-OCT-2020
0.3	Updated following review by Gareth Griffiths and Dean Fennell	Kayleigh Hill	25-OCT-2020
1.0 [final]	Updated following review by Sean Ewings and Dean Fennell	Sean Ewings	26-OCT-2020

[It should be clearly noted in the revision history, which is the final version of the SAP and which version of the protocol the final SAP is based on]

[The version history table should clearly state which version is the final SAP (i.e. final version of the SAP which is a comprehensive and detailed description of the methods and presentation of the data analyses) and which version of the protocol this was based on]

[The revision history table should provide a list of major/key changes to each version of the SAP]

Collaborators

CONFIRM trial Investigators

Gillian Price[1], Nicola Steele[2], Paul Shaw[3], Judith Cave[4], Jay Naik[5], Amy Ford[6], Tom Geldhart[7], Gairin Dancey[8,16], Dionysis Papadatos[9], Andy Polychronis[10,11], Petra Jankowska[12], Angela Scott[13], Jill Gardiner[14], Mathilda Cominos[15], Lynn Campbell[17], Carol MacGregor[18], Lois Mullholand[19], Meenali Chitnis[20], Gary Dougherty[21]

Affiliations

[1]Aberdeen Royal Infirmary; [2]Beatson West of Scotland Cancer Centre[2]; [3]Velindre Cancer Centre; [4]University of Southampton NHS Trust; [5]York/Harrogate NHS Trust; [6]University Hospitals of Morcambe Bay NHS Trust; [7]The Royal Bournemouth and Christchurch NHS Foundation Trust Hospital; [8]Southend University Hospital NHS Trust, [9]University College London Hospitals NHS Foundation Trust, [10]Lister Hospital, [11]Mount Vernon Hospital, [12]Musgrove Park Hospital; [13]Ninewells Hospital, Dundee; [14]Northumbria Healthcare NHS Foundation Trust; [15]East Kent Hospitals University NHS Foundation Trust, [16]Basildon University Hospital, [17]Belfast Health and Social Care Trust, [18]Raigmore Hospital, [19]Ulster Hospital; [20]Oxford University Hospitals NHS Foundation Trust; [21]Addenbrookes Hospital Cambridge

PI name	Site name	Total number of patients recruited in CONFIRM
Dean Fennell	Leicester	85
Raffaele Califano	Wythenshawe	54
Christian Ottensmeier	Southampton	37
Jason Lester	Velindre	20
Sam Chan	York/Harrogate	18
Nicola Steele	Beatson	17
Petra Jankowska	Musgrove park	14
Peter Szlosarek	St Barts	11
Dionysis Papadatos	UCLH	10
Gairin Dancey	Southend	9
Tom Geldart	Bournemouth	7
Mathilda Cominos	East Kent	6
Gillian Price	Aberdeen	5
Amy Ford	Morecambe	5
Andy Polychronis	Lister	5
Jill Gardiner	Northumbria	5
Gary Dougherty	Addenbrookes	5
Angela Scott	Ninewells	4
Lynn Campbell	Belfast	4
Andy Polychronis	Mount Vernon	3
Meenali Chitnis	Oxford	3
Lois Mulholland	Ulster	2
Gairin Dancey	Basildon	1
Carol MacGregor	Raigmore	1

Figure S1. Kaplan Meier curves showing progression free survival stratified by histology A. Epithelioid, B. Non-Epithelioid

A

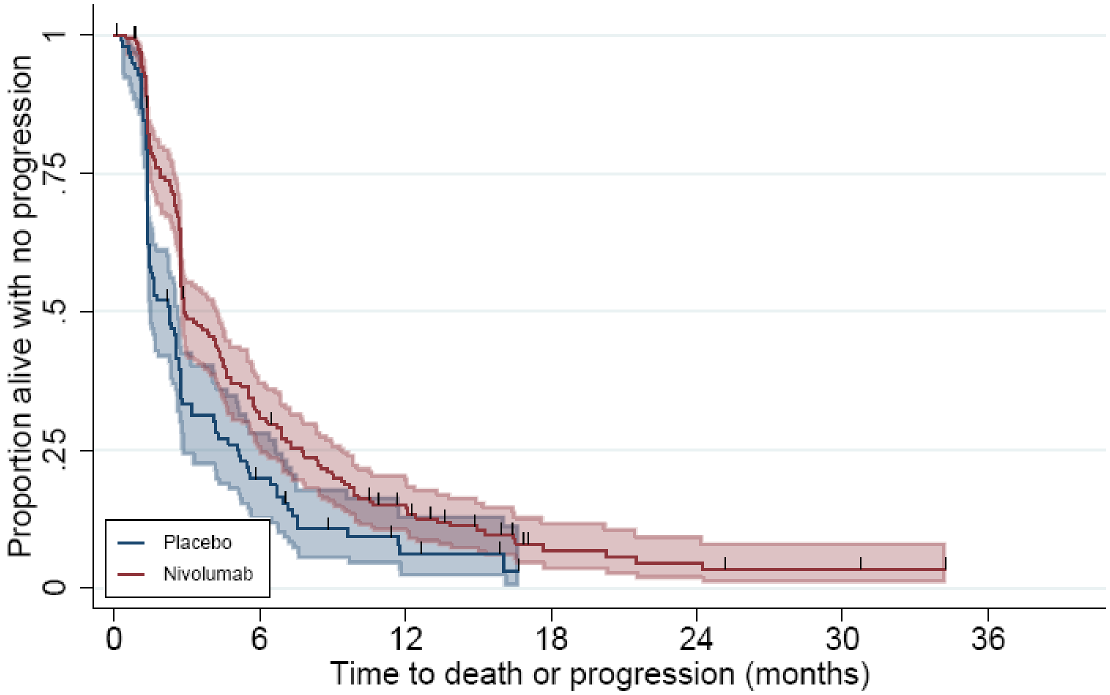

**Placebo**							
At-risk (cens)	98(0)	18(2)	4(5)	0(8)	0(8)	0(8)	0(8)
Died/Progressed	0	78	89	90	90	90	90
**Nivolumab**							
At-risk (cens)	195(0)	58(5)	25(9)	6(17)	4(17)	2(18)	0(20)
Died/Progressed	0	132	161	172	174	175	175

B

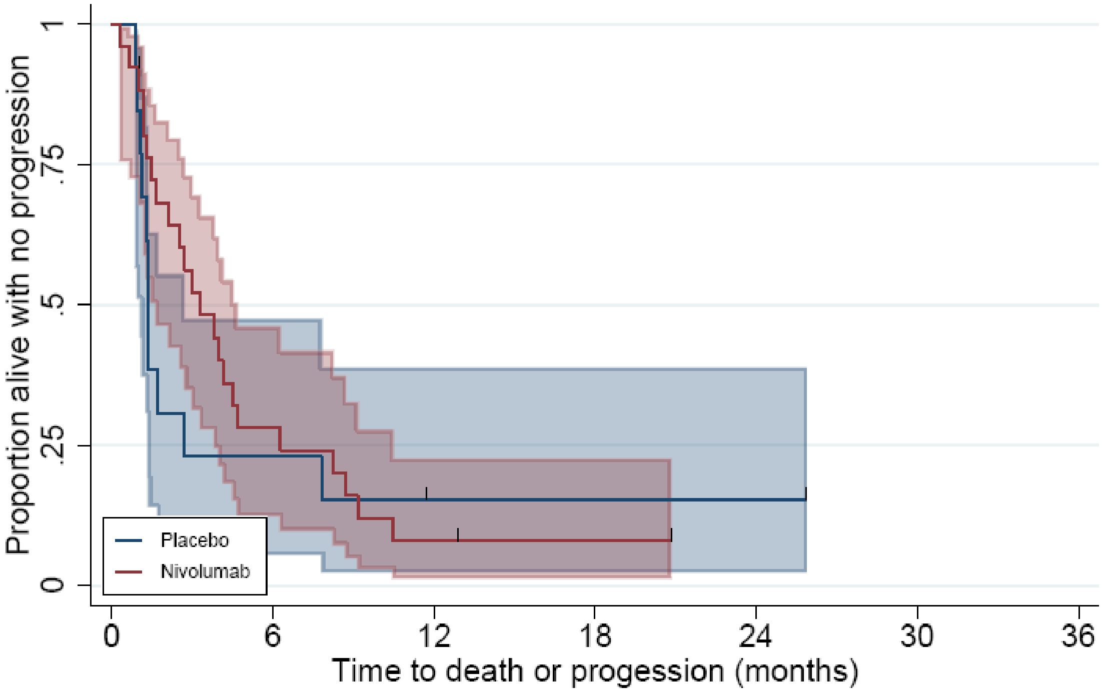

**Placebo**							
At-risk (cens)	13(0)	3(0)	1(1)	1(1)	1(1)	0(2)	0(2)
Died/Progressed	0	10	11	11	11	11	11
**Nivolumab**							
At-risk (cens)	26(0)	7(1)	2(1)	1(2)	0(3)	0(3)	0(3)
Died/Progressed	0	18	23	23	23	23	23

Figure S2. Kaplan Meier curves showing progression free survival stratified by PDL1 A. Positive and B. Negative

A

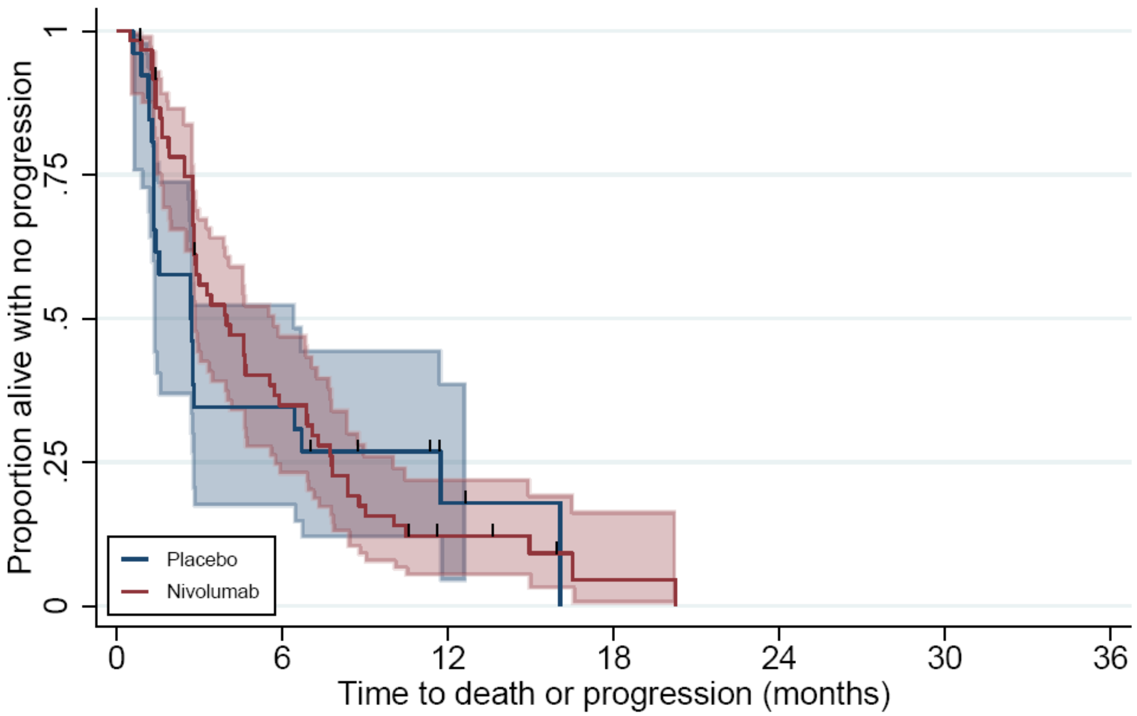

	0	6	12	18	24	30	36
**Placebo**							
At-risk (cens)	26(0)	9(0)	2(4)	0(5)	0(5)	0(5)	0(5)
Died/Progressed	0	17	20	21	21	21	21
**Nivolumab**							
At-risk (cens)	61(0)	20(3)	5(5)	1(7)	0(7)	0(7)	0(7)
Died/Progressed	0	38	51	53	54	54	54

B

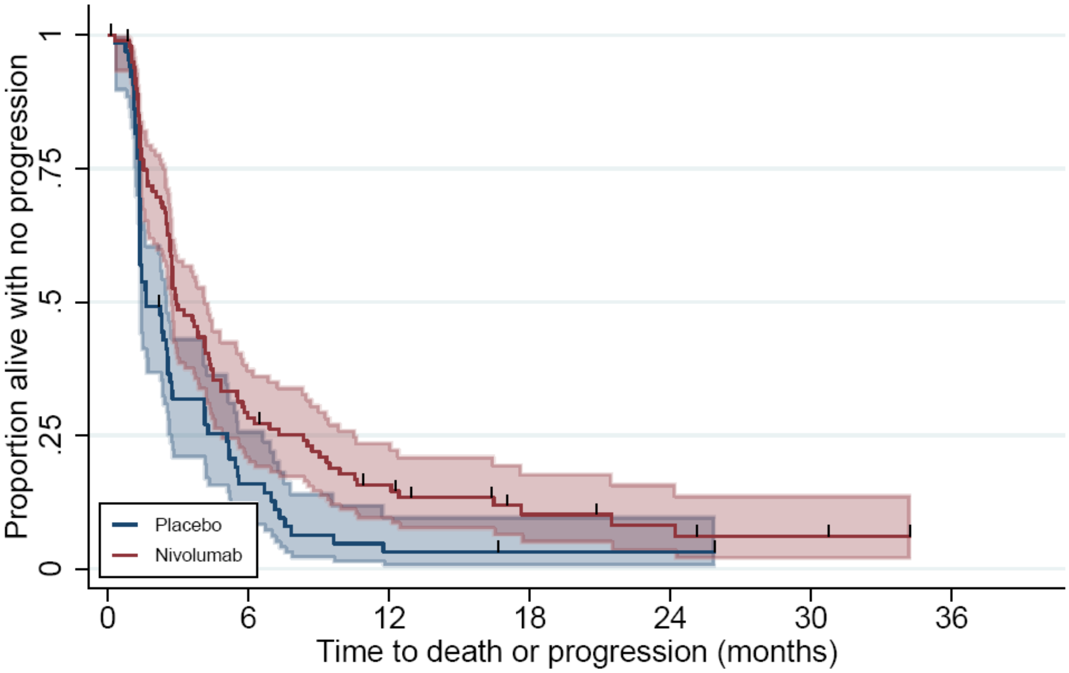

	0	6	12	18	24	30	36
**Placebo**							
At-risk (cens)	65(0)	10(1)	2(1)	1(2)	1(2)	0(3)	0(3)
Died/Progressed	0	54	62	62	62	62	62
**Nivolumab**							
At-risk (cens)	101(0)	28(2)	14(4)	6(8)	4(9)	2(10)	0(12)
Died/Progressed	0	71	83	87	88	89	89

Figure S3. Kaplan Meier curves showing overall survival stratified by histology A. Epithelioid, B. Non-Epithelioid

A

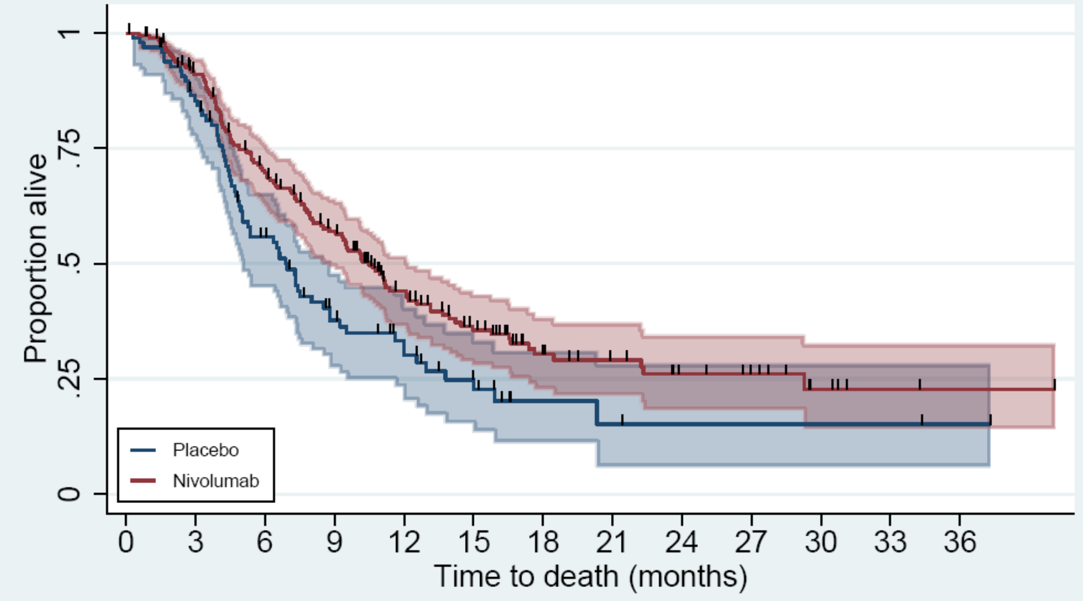

	0	3	6	9	12	15	18	21	24	27	30	33	36
**Placebo**													
At-risk	98	80	48	28	19	12	4	3	2	2	2	2	1
Censored	0	4	9	14	18	22	28	28	29	29	29	29	30
Died	0	14	41	56	61	64	66	67	67	67	67	67	67
**Nivolumab**													
At-risk	195	168	125	96	61	42	25	20	14	11	5	2	1
Censored	0	10	14	21	36	44	56	60	64	67	72	75	76
Died	0	17	56	78	98	109	114	115	117	117	118	118	118

B

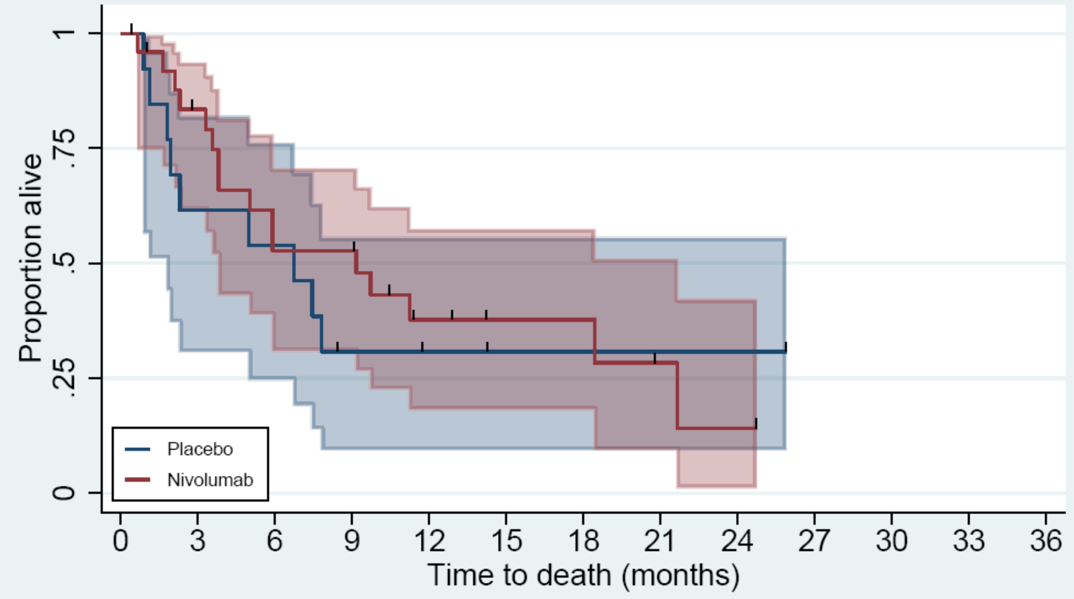

	0	3	6	9	12	15	18	21	24	27	30	33	36
**Placebo**													
At-risk	13	8	7	3	2	1	1	1	1	0	0	0	0
Censored	0	0	0	1	2	3	3	3	3	4	4	4	4
Died	0	5	6	9	9	9	9	9	9	9	9	9	9
**Nivolumab**													
At-risk	26	19	12	12	6	4	4	2	1	0	0	0	0
Censored	0	3	3	3	6	8	8	9	9	10	10	10	10
Died	0	4	11	11	14	14	14	15	16	16	16	16	16

Figure S4. Kaplan Meier curves showing overall survival stratified by PDL1 A. Positive and B. Negative

A

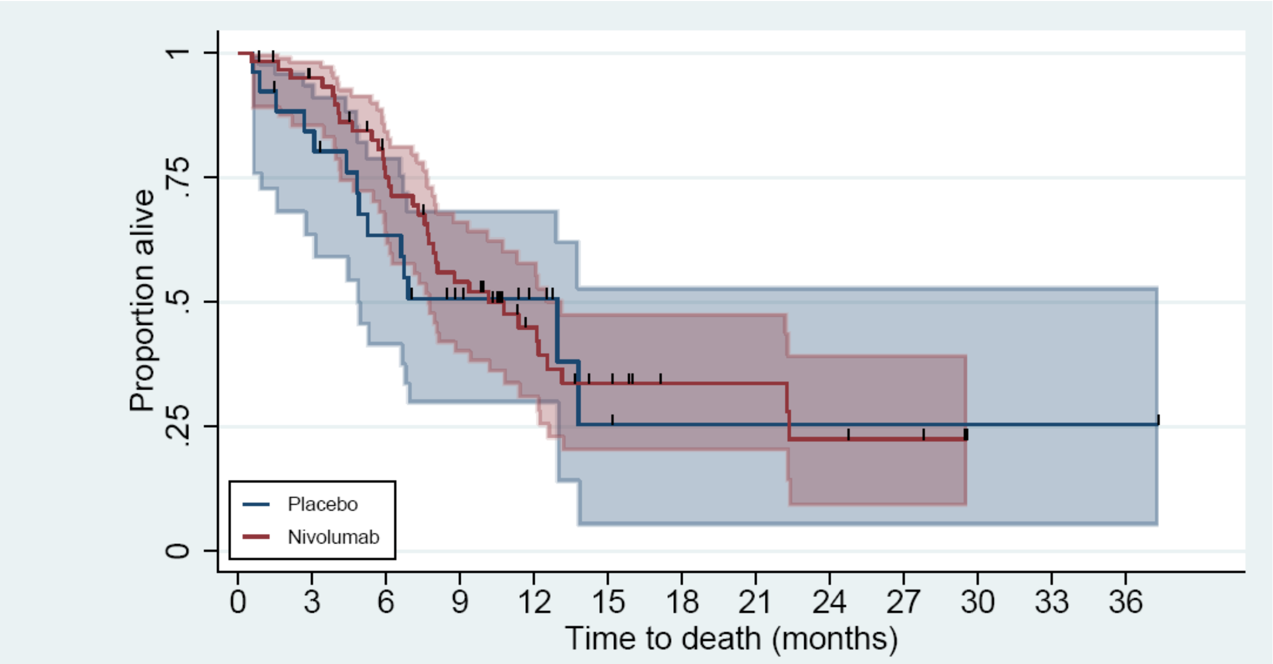

**Placebo**													
At-risk	26	21	15	9	6	2	1	1	1	1	1	1	1
Censored	0	1	2	5	8	10	11	11	11	11	11	11	11
Died	0	4	9	12	12	14	14	14	14	14	14	14	14
**Nivolumab**													
At-risk	61	54	40	28	16	10	6	6	4	3	0	0	0
Censored	0	4	7	8	16	18	22	22	22	23	26	26	26
Died	0	3	14	25	29	33	33	33	35	35	35	35	35

B

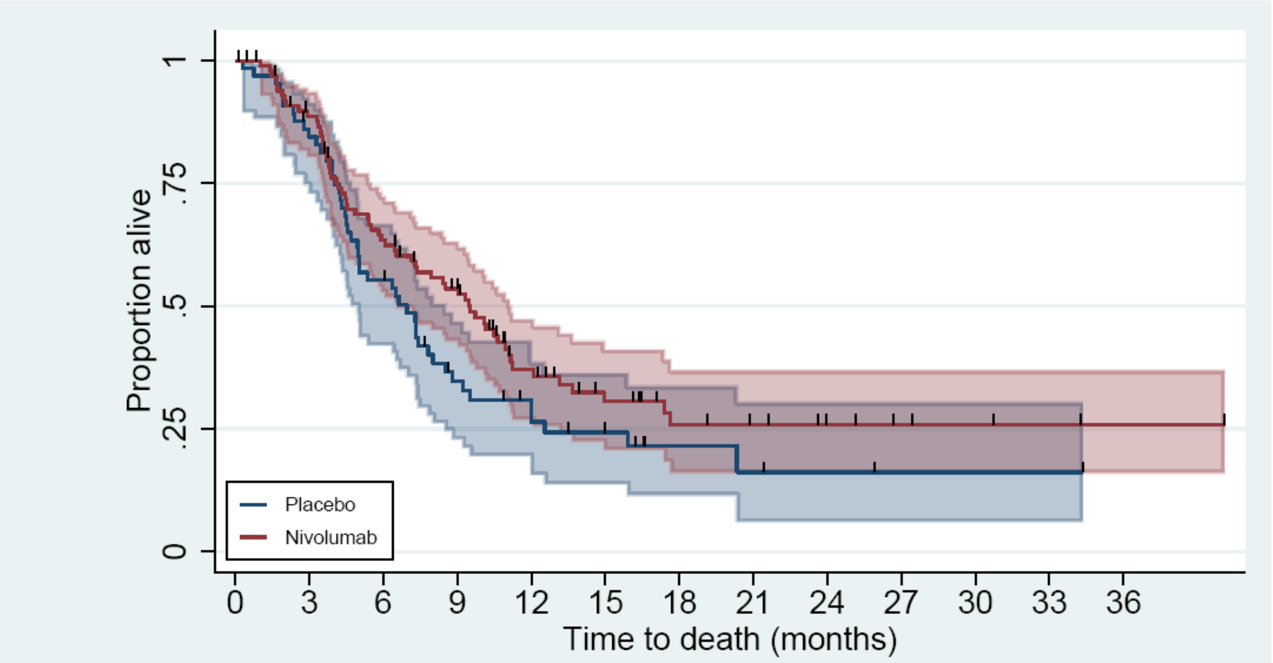

**Placebo**													
At-risk	65	53	33	18	12	9	4	3	2	1	1	1	0
Censored	0	2	4	7	9	11	15	15	16	17	17	17	18
Died	0	10	28	40	44	45	46	47	47	47	47	47	47
**Nivolumab**													
At-risk	101	85	60	47	26	17	11	9	6	4	3	2	1
Censored	0	5	6	10	18	23	27	29	32	34	35	36	37
Died	0	11	35	44	57	61	63	63	63	63	63	63	63

Cox regression results for progression free survival with PD-L1 as two (<1% or ≥1%) and three categories (<1%, 1-49% or ≥50%), including interaction with treatment.

Progression-free survival	Hazard ratio	95% CI	p-value
Treatment	0.59	0.42 to 0.82	0.002
PD-L1 positive	0.63	0.38 to 1.03	0.066
PD-L1-treatment interaction	1.54	0.84 to 2.82	0.159
Treatment	0.74	0.53 to 1.03	0.073
PD-L1 low positive (1-49%)	0.73	0.42 to 1.25	0.245
PD-L1 high positive (≥50%)	1.32	0.48 to 3.68	0.586
PD-L1 low positive-treatment interaction	1.42	0.74 to 2.73	0.292
PD-L1 high positive-treatment interaction	0.58	0.17 to 1.94	0.379

All analyses controlled for epithelioid status. N=253 included in analysis; n=166 negative, n=87 positive (of which n=72 low positive).

Cox regression results for Overall Survival with PD-L1 as two (<1% or ≥1%) and three categories (<1%, 1-49% or ≥50%), including interaction with treatment.

**Overall survival**	**Hazard ratio**	**95% CI**	**p-value**
Treatment	0.74	0.51 to 1.08	0.119
PD-L1 positive	0.73	0.40 to 1.32	0.296
PD-L1-treatment interaction	1.17	0.57 to 2.43	0.669
Treatment	0.74	0.51 to 1.08	0.118
PD-L1 low positive (1-49%)	0.65	0.34 to 1.26	0.203
PD-L1 high positive (≥50%)	1.23	0.38 to 4.00	0.730
PD-L1 low positive-treatment interaction	1.44	0.65 to 3.17	0.365
PD-L1 high positive-treatment interaction	0.44	0.10 to 1.87	0.807

All analyses controlled for epithelioid status. N=253 included in analysis; n=166 negative, n=87 positive (of which n=72 low positive).

Summary of all Serious Adverse Events

	Nivolumab (n=221)	Placebo (n=111)	Total (n=332)
**Number of patients experiencing AE**	**90 (40.7%)**	**49 (44.1%)**	**139 (41.9%)**
**Blood and lymphatic system disorders**	**3 (1.4%)**	**1 (0.9%)**	**4 (1.2%)**
Anaemia	2 (0.9%)	1 (0.9%)	3 (0.9%)
Neutropenia	1 (0.5%)	0 (0.0%)	1 (0.3%)
**Cardiac disorders**	**8 (3.6%)**	**0 (0.0%)**	**8 (2.4%)**
Acute Coronary Syndrome	1 (0.5%)	0 (0.0%)	1 (0.3%)
Atrioventricular block	1 (0.5%)	0 (0.0%)	1 (0.3%)
Pericardial effusion	4 (1.8%)	0 (0.0%)	4 (1.2%)
Sinus tachycardia	1 (0.5%)	0 (0.0%)	1 (0.3%)
Supraventricular tachycardia	1 (0.5%)	0 (0.0%)	1 (0.3%)
**Endocrine disorders**	**1 (0.5%)**	**0 (0.0%)**	**1 (0.3%)**
Hypothyroidism	1 (0.5%)	0 (0.0%)	1 (0.3%)
**Eye disorders**	**2 (0.9%)**	**0 (0.0%)**	**2 (0.6%)**
Diplopia	1 (0.5%)	0 (0.0%)	1 (0.3%)
Opthalmoplegia	1 (0.5%)	0 (0.0%)	1 (0.3%)
**Gastrointestinal disorders**	**12 (5.4%)**	**10 (9.0%)**	**22 (6.6%)**
Abdominal pain	4 (1.8%)	0 (0.0%)	4 (1.2%)
Ascites	0 (0.0%)	1 (0.9%)	1 (0.3%)
Colitis	3 (1.4%)	0 (0.0%)	3 (0.9%)
Colitis microscopic	1 (0.5%)	0 (0.0%)	1 (0.3%)
Constipation	0 (0.0%)	2 (1.8%)	2 (0.6%)
Diarrhoea	5 (2.3%)	5 (4.5%)	10 (3.0%)
Ileus	0 (0.0%)	1 (0.9%)	1 (0.3%)
Intestinal obstruction	0 (0.0%)	1 (0.9%)	1 (0.3%)
Nausea	0 (0.0%)	1 (0.9%)	1 (0.3%)
Stomatitis	1 (0.5%)	0 (0.0%)	1 (0.3%)
**General disorders and administration site conditions**	**6 (2.7%)**	**10 (9.0%)**	**16 (4.8%)**
Chest pain	0 (0.0%)	1 (0.9%)	1 (0.3%)
Disease Progression	0 (0.0%)	3 (2.7%)	3 (0.9%)
Fatigue	0 (0.0%)	1 (0.9%)	1 (0.3%)
General physical health deterioration	2 (0.9%)	0 (0.0%)	2 (0.6%)

Malaise	0 (0.0%)	1 (0.9%)	1 (0.3%)
Oedema peripheral	0 (0.0%)	1 (0.9%)	1 (0.3%)
Pain	3 (1.4%)	1 (0.9%)	4 (1.2%)
Pyrexia	1 (0.5%)	2 (1.8%)	3 (0.9%)
**Hepatobiliary disorders**	**2 (0.9%)**	**0 (0.0%)**	**2 (0.6%)**
Autoimmune hepatitis	2 (0.9%)	0 (0.0%)	2 (0.6%)
**Infections and infestations**	**27 (12.2%)**	**17 (15.3%)**	**44 (13.3%)**
Bronchitis	1 (0.5%)	0 (0.0%)	1 (0.3%)
COVID-19	1 (0.5%)	0 (0.0%)	1 (0.3%)
Herpes zoster	1 (0.5%)	0 (0.0%)	1 (0.3%)
Infection	3 (1.4%)	3 (2.7%)	6 (1.8%)
Lower respiratory tract infection	8 (3.6%)	8 (7.2%)	16 (4.8%)
Neutropenic Sepsis	1 (0.5%)	0 (0.0%)	1 (0.3%)
Pilonidal Cyst	1 (0.5%)	0 (0.0%)	1 (0.3%)
Pneumonia	14 (6.3%)	6 (5.4%)	20 (6.0%)
Respiratory Tract Infection	0 (0.0%)	1 (0.9%)	1 (0.3%)
Sepsis	0 (0.0%)	1 (0.9%)	1 (0.3%)
**Injury, Poisoning and procedural complications**	**6 (2.7%)**	**2 (1.8%)**	**8 (2.4%)**
Fall	1 (0.5%)	0 (0.0%)	1 (0.3%)
Femoral neck fracture	1 (0.5%)	0 (0.0%)	1 (0.3%)
Hip Fracture	1 (0.5%)	0 (0.0%)	1 (0.3%)
Infusion related reaction	4 (1.8%)	1 (0.9%)	5 (1.5%)
Toxicity to various agents	0 (0.0%)	1 (0.9%)	1 (0.3%)
**Investigations**	**6 (2.7%)**	**1 (0.9%)**	**7 (2.1%)**
Alanine aminotransferase increased	4 (1.8%)	0 (0.0%)	4 (1.2%)
Aspartate aminotransferase increased	3 (1.4%)	0 (0.0%)	3 (0.9%)
Blood alkaline phosphatase increased	1 (0.5%)	0 (0.0%)	1 (0.3%)
Blood bilirubin increased	1 (0.5%)	0 (0.0%)	1 (0.3%)
Blood creatinine increased	0 (0.0%)	1 (0.9%)	1 (0.3%)

Gamma-glutamyltransferase increased	1 (0.5%)	0 (0.0%)	1 (0.3%)
Oxygen Saturation Decreased	1 (0.5%)	0 (0.0%)	1 (0.3%)
Transaminases	1 (0.5%)	0 (0.0%)	1 (0.3%)
**Metabolism and nutrition disorders**	**0 (0.0%)**	**5 (4.5%)**	**5 (1.5%)**
Dehydration	0 (0.0%)	2 (1.8%)	2 (0.6%)
Diabetic ketoacidosis	0 (0.0%)	1 (0.9%)	1 (0.3%)
Hyperkalaemia	0 (0.0%)	1 (0.9%)	1 (0.3%)
Hyponatraemia	0 (0.0%)	1 (0.9%)	1 (0.3%)
**Musculoskeletal and connective tissue disorders**	**4 (1.8%)**	**1 (0.9%)**	**5 (1.5%)**
Arthritis	1 (0.5%)	0 (0.0%)	1 (0.3%)
Back pain	2 (0.9%)	0 (0.0%)	2 (0.6%)
Musculoskeletal chest pain	0 (0.0%)	1 (0.9%)	1 (0.3%)
Myositis	1 (0.5%)	0 (0.0%)	1 (0.3%)
**Neoplasms benign, malignant and unspecified (incl cysts and polyps)**	**5 (2.3%)**	**1 (0.9%)**	**6 (1.8%)**
Bladder neoplasm	1 (0.5%)	0 (0.0%)	1 (0.3%)
Mesothelioma	1 (0.5%)	0 (0.0%)	1 (0.3%)
Mesothelioma malignant	1 (0.5%)	0 (0.0%)	1 (0.3%)
Myelodysplastic Syndrome	1 (0.5%)	0 (0.0%)	1 (0.3%)
Squamous cell carcinoma of lung	0 (0.0%)	1 (0.9%)	1 (0.3%)
mesothelioma	1 (0.5%)	0 (0.0%)	1 (0.3%)
**Nervous system disorders**	**7 (3.2%)**	**3 (2.7%)**	**10 (3.0%)**
Ataxia	1 (0.5%)	0 (0.0%)	1 (0.3%)
Cerebral Hypoperfusion	1 (0.5%)	0 (0.0%)	1 (0.3%)
Cerebral haemorrhage	1 (0.5%)	0 (0.0%)	1 (0.3%)
Cerebrovascular accident	1 (0.5%)	1 (0.9%)	2 (0.6%)
Dizziness	1 (0.5%)	0 (0.0%)	1 (0.3%)
Facial Paralysis	2 (0.9%)	0 (0.0%)	2 (0.6%)
Spinal cord	0 (0.0%)	2 (1.8%)	2 (0.6%)

compression			
Syncope	1 (0.5%)	0 (0.0%)	1 (0.3%)
**Psychiatric disorders**	**3 (1.4%)**	**1 (0.9%)**	**4 (1.2%)**
Anxiety	1 (0.5%)	0 (0.0%)	1 (0.3%)
Confusional State	2 (0.9%)	1 (0.9%)	3 (0.9%)
**Renal and urinary disorders**	**0 (0.0%)**	**1 (0.9%)**	**1 (0.3%)**
Acute Kidney Injury	0 (0.0%)	1 (0.9%)	1 (0.3%)
**Reproductive system and breast disorders**	**0 (0.0%)**	**1 (0.9%)**	**1 (0.3%)**
Prostatitis	0 (0.0%)	1 (0.9%)	1 (0.3%)
**Respiratory, thoracic and mediastinal disorders**	**27 (12.2%)**	**14 (12.6%)**	**41 (12.3%)**
Dyspnoea	18 (8.1%)	10 (9.0%)	28 (8.4%)
Painful respiration	0 (0.0%)	1 (0.9%)	1 (0.3%)
Pleural effusion	3 (1.4%)	0 (0.0%)	3 (0.9%)
Pneumonia Aspiration	1 (0.5%)	0 (0.0%)	1 (0.3%)
Pneumonitis	2 (0.9%)	1 (0.9%)	3 (0.9%)
Pulmonary embolism	3 (1.4%)	1 (0.9%)	4 (1.2%)
Pulmonary oedema	0 (0.0%)	1 (0.9%)	1 (0.3%)
**Surgical and medical procedures**	**2 (0.9%)**	**0 (0.0%)**	**2 (0.6%)**
Abdominal cavity drainage	1 (0.5%)	0 (0.0%)	1 (0.3%)
Pain management	1 (0.5%)	0 (0.0%)	1 (0.3%)
**Vascular disorders**	**2 (0.9%)**	**0 (0.0%)**	**2 (0.6%)**
Deep vein thrombosis	1 (0.5%)	0 (0.0%)	1 (0.3%)
Hypotension	1 (0.5%)	0 (0.0%)	1 (0.3%)

Summary of Treatment Related Adverse Events Grade 3 or above

	Nivolumab (n=221)	Placebo (n=111)	Total (n=332)
**Number of patients experiencing AE**	**42 (19.0%)**	**7 (6.3%)**	**49 (14.8%)**
**Blood and lymphatic system disorders**	**2 (0.9%)**	**0 (0.0%)**	**2 (0.6%)**
Anaemia	1 (0.5%)	0 (0.0%)	1 (0.3%)
Thrombocytopenia	1 (0.5%)	0 (0.0%)	1 (0.3%)
**Cardiac disorders**	**1 (0.5%)**	**1 (0.9%)**	**2 (0.6%)**
Cardiac dysfunction	1 (0.5%)	0 (0.0%)	1 (0.3%)
Tachycardia	0 (0.0%)	1 (0.9%)	1 (0.3%)
**Endocrine disorders**	**1 (0.5%)**	**0 (0.0%)**	**1 (0.3%)**
Hypothyroidism	1 (0.5%)	0 (0.0%)	1 (0.3%)
**Eye disorders**	**0 (0.0%)**	**1 (0.9%)**	**1 (0.3%)**
Macular oedema	0 (0.0%)	1 (0.9%)	1 (0.3%)
**Gastrointestinal disorders**	**12 (5.4%)**	**2 (1.8%)**	**14 (4.2%)**
Abdominal pain	1 (0.5%)	0 (0.0%)	1 (0.3%)
Ascites	1 (0.5%)	0 (0.0%)	1 (0.3%)
Colitis	3 (1.4%)	0 (0.0%)	3 (0.9%)
Colitis microscopic	1 (0.5%)	0 (0.0%)	1 (0.3%)
Diarrhoea	6 (2.7%)	2 (1.8%)	8 (2.4%)
Stomatitis	2 (0.9%)	0 (0.0%)	2 (0.6%)
Vomiting	1 (0.5%)	0 (0.0%)	1 (0.3%)
**General disorders and administration site conditions**	**2 (0.9%)**	**2 (1.8%)**	**4 (1.2%)**
Chest pain	1 (0.5%)	0 (0.0%)	1 (0.3%)
Fatigue	1 (0.5%)	1 (0.9%)	2 (0.6%)
Generalised oedema	1 (0.5%)	0 (0.0%)	1 (0.3%)
Malaise	0 (0.0%)	1 (0.9%)	1 (0.3%)
**Hepatobiliary disorders**	**3 (1.4%)**	**0 (0.0%)**	**3 (0.9%)**
Autoimmune hepatitis	2 (0.9%)	0 (0.0%)	2 (0.6%)
Hepatotoxicity	1 (0.5%)	0 (0.0%)	1 (0.3%)
**Injury, poisoning and procedural complications**	**6 (2.7%)**	**0 (0.0%)**	**6 (1.8%)**
Infusion related	6 (2.7%)	0 (0.0%)	6 (1.8%)

reaction			
**Investigations**	**6 (2.7%)**	**1 (0.9%)**	**7 (2.1%)**
Alanine aminotransferase increased	4 (1.8%)	0 (0.0%)	4 (1.2%)
Aspartate aminotransferase increased	4 (1.8%)	0 (0.0%)	4 (1.2%)
Blood alkaline phosphatase increased	4 (1.8%)	0 (0.0%)	4 (1.2%)
Blood bilirubin increased	1 (0.5%)	0 (0.0%)	1 (0.3%)
Blood creatinine increased	0 (0.0%)	1 (0.9%)	1 (0.3%)
Gamma-glutamyltransferase increased	2 (0.9%)	0 (0.0%)	2 (0.6%)
Lipase increased	1 (0.5%)	0 (0.0%)	1 (0.3%)
**Metabolism and nutrition disorders**	**0 (0.0%)**	**1 (0.9%)**	**1 (0.3%)**
Hyponatraemia	0 (0.0%)	1 (0.9%)	1 (0.3%)
**Musculoskeletal and connective tissue disorders**	**4 (1.8%)**	**0 (0.0%)**	**4 (1.2%)**
Arthralgia	1 (0.5%)	0 (0.0%)	1 (0.3%)
Arthritis	1 (0.5%)	0 (0.0%)	1 (0.3%)
Back pain	1 (0.5%)	0 (0.0%)	1 (0.3%)
Myositis	1 (0.5%)	0 (0.0%)	1 (0.3%)
**Nervous system disorders**	**5 (2.3%)**	**0 (0.0%)**	**5 (1.5%)**
Ataxia	1 (0.5%)	0 (0.0%)	1 (0.3%)
Carpal tunnel syndrome	1 (0.5%)	0 (0.0%)	1 (0.3%)
Facial paralysis	1 (0.5%)	0 (0.0%)	1 (0.3%)
Headache	1 (0.5%)	0 (0.0%)	1 (0.3%)
Tremor	1 (0.5%)	0 (0.0%)	1 (0.3%)
**Psychiatric disorders**	**1 (0.5%)**	**0 (0.0%)**	**1 (0.3%)**
Anxiety	1 (0.5%)	0 (0.0%)	1 (0.3%)
**Reproductive system and breast disorders**	**0 (0.0%)**	**1 (0.9%)**	**1 (0.3%)**
Prostatism	0 (0.0%)	1 (0.9%)	1 (0.3%)

**Respiratory, thoracic and mediastinal disorders**	**6 (2.7%)**	**1 (0.9%)**	**7 (2.1%)**
Chronic obstructive pulmonary disease	1 (0.5%)	0 (0.0%)	1 (0.3%)
Dyspnoea	2 (0.9%)	1 (0.9%)	3 (0.9%)
Dyspnoea exertional	1 (0.5%)	0 (0.0%)	1 (0.3%)
Immune-mediated pneumonitis	1 (0.5%)	0 (0.0%)	1 (0.3%)
Pneumonitis	1 (0.5%)	0 (0.0%)	1 (0.3%)
Pulmonary embolism	1 (0.5%)	0 (0.0%)	1 (0.3%)
**Skin and subcutaneous tissue disorders**	**1 (0.5%)**	**0 (0.0%)**	**1 (0.3%)**
Erythema	1 (0.5%)	0 (0.0%)	1 (0.3%)
**Unclassified[1]**	**3 (1.4%)**	**0 (0.0%)**	**3 (0.9%)**

[1]These Adverse Events are pending classification by a final clinical review.

Reasons for discontinuation of treatment due to Unacceptable Toxicity

	Nivolumab (n=221)	Placebo (n=111)	Total (n=332)
**Ended treatment due to Unacceptable Toxicity - n (%)**	30/217 (14)	3/110 (3)	33/327 (11)
**Reason**			
Adverse drug reaction	1 (3)	0 (0)	1 (3)
Alanine aminotransferase increased	1 (3)	0 (0)	1 (3)
Arthropathy	1 (3)	0 (0)	1 (3)
Aspartate aminotransferase increased	1 (3)	0 (0)	1 (3)
Ataxia	1 (3)	0 (0)	1 (3)
Blood alkaline phosphatase increased	1 (3)	0 (0)	1 (3)
Colitis	2 (7)	0 (0)	2 (6)
Diarrhoea	3 (10)	0 (0)	3 (9)
Fatigue	1 (3)	0 (0)	1 (3)
Gamma-glutamyltransferase increased	1 (3)	0 (0)	1 (3)
Headache	1 (3)	0 (0)	1 (3)
Hepatotoxicity	1 (3)	0 (0)	1 (3)
Hyponatraemia	0 (0)	1 (33)	1 (3)
Immune-mediated pneumonitis	1 (3)	0 (0)	1 (3)
Infusion related reaction	4 (13)	0 (0)	4 (12)
Myalgia	1 (3)	0 (0)	1 (3)
Myositis	1 (3)	0 (0)	1 (3)
Pancreatitis	1 (3)	0 (0)	1 (3)
Pneumonia	1 (3)	1 (33)	2 (6)
Pneumonitis	1 (3)	0 (0)	1 (3)
Rash	1 (3)	0 (0)	1 (3)
Stomatitis	1 (3)	0 (0)	1 (3)
Urinary tract infection	1 (3)	0 (0)	1 (3)
Missing	2 (7)	1 (33)	2 (6)

Summary of SAEs related to death

	Nivolumab (n=221)	Placebo (n=111)	Total (n=332)
**SAE related to death**	10 (5)	7 (6)	17 (5)
SAE term – n(%)			
**Cerebrovascular accident**	0 (0)	1 (0)	1 (0)
**Disease Progression**	0 (0)	2 (2)	2 (1)
**Dyspnoea**	1 (1)	2 (2)	3 (1)
**General physical health deterioration**	1 (1)	0 (0)	1 (0)
**Infection**	0 (0)	1 (1)	1 (0)
**Mesothelioma**	1 (1)	0 (0)	1 (0)
**Mesothelioma malignant**	1 (1)	0 (0)	1 (0)
**Oxygen Saturation Decreased**	1 (1)	0 (0)	1 (0)
**Pericardial effusion**	1 (1)	0 (0)	1 (0)
**Pneumonia**	2 (1)	0 (0)	2 (1)
**Pulmonary embolism**	2 (1)	1 (1)	3 (1)

Number of delays	Nivolumab	Placebo	Total
1	66	26	92
2	18	5	23
3	8	3	11
4	2	0	2
5	2	0	2
Total	96	34	130

Number of people with a dose delay

Duration of delays:

- Nivolumab – median 14 days (range 1 to 263)
- Placebo – median 7.5 days (range 1 to 43)

Treatment	Nivolumab	Placebo	Total
Avastin/Platinum/Pemetrexed	2	1	3
Other platinum doublet	8	2	10
Pemetrexed/Carboplatin	108	55	163
Pemetrexed/Cisplatin	103	53	156

First line of treatment

CONFIRM

Checkp**O**i**N**t blockade **F**or **I**nhibition of **R**elapsed **M**esothelioma (CONFIRM): A Phase III Double-Blind, Placebo Controlled Trial to Evaluate the Efficacy of Nivolumab in Relapsed Mesothelioma

BMS protocol number: CA209-841

Version 7 11 Jun 2020

SPONSOR: University of Southampton
COORDINATING CENTRE: Southampton Clinical Trials Unit

EudraCT	2016-003111-35
ISRCTN reference:	79814141
ClinicalTrials.gov reference:	NCT03063450
Ethics reference number:	16/WM/0472
Sponsor reference number:	22864
Funder reference number:	C16728/A21400
ICD10	C45.9

Protocol authorised by:

**Name:**	Professor Dean Fennell	**Role:**	Chief Investigator (CI)
**Signature:**		**Date:**	
**Name:**	Professor Gareth Griffiths	**Role:**	Director of SCTU
**Signature:**		**Date:**	
**Name:**		**Role:**	Sponsor Rep
**Signature:**		**Date:**	

MAIN TRIAL CONTACT

Chief Investigator and Medical Expert: Professor Dean Fennell

Leicester Cancer Research Centre
Mesothelioma Research Programme
Leicester Cancer Research Centre,
University of Leicester &
University Hospitals of Leicester NHS Trust
4th Floor, Robert Kilpatrick Building,
Leicester, LE2 7LX, UK

Tel: +44 (0)116 252 3170
Email: df132@le.ac.uk

TRIAL COORDINATION CENTRE

For general trial and clinical queries e.g. participant queries, trial supplies, data collection, please contact in the first instance:

CONFIRM Trial Manager
Tel: 023 8120 4307
Email: confirmtrial@soton.ac.uk

Address: Southampton Clinical Trials Unit
Southampton General Hospital
Tremona Road
Southampton
SO16 6YD

Tel: 023 8120 5154
Fax: 0844 774 0621
Email: ctu@soton.ac.uk
Web: www.southampton.ac.uk/ctu

SPONSOR

The University of Southampton is the research sponsor for this trial. For further information regarding sponsorship conditions, please contact Research & Innovation Services at:

Address: Research & Innovation Services University of Southampton

Tel: 023 8059 3095
Fax: 023 8059 3585

CO-INVESTIGATORS

Co-Investigators can be contacted via the Trial Coordination Centre.

Professor Gareth Griffiths, University Of Southampton (Co-Chief Investigator)
Dr Jason Lester, Velindre NHS Trust
Dr Gerard Hanna, Queen's University Belfast
Dr Nicola Steele, Beatson West Of Scotland Cancer Centre
Dr Peter Szlosarek, Queen Mary, University Of London
Professor Sarah Danson, University Of Sheffield
Professor Joanne Lord, University Of Southampton
Professor Christian Ottensmeier, University Of Southampton

This trial is primarily funded by Cancer Research UK with additional support from Bristol-Myers Squibb.

Protocol Information

This protocol describes the CONFIRM trial and provides information about procedures for entering participants. The protocol should not be used as a guide for the treatment of other non-trial participants; every care was taken in its drafting, but corrections or amendments may be necessary. These will be circulated to investigators in the trial, but sites entering participants for the first time are advised to contact Southampton Clinical Trials Unit to confirm they have the most recent version.

Compliance

This trial will adhere to the principles of Good Clinical Practice (GCP). It will be conducted in compliance with the protocol, in accordance with current Data Protection Regulations and all other regulatory requirements, as appropriate.

TABLE OF CONTENTS

LIST OF ABBREVIATIONS 7
TRIAL SYNOPSIS 9
SCHEDULE OF OBSERVATIONS AND PROCEDURES 12
1 INTRODUCTION 14
1.1. BACKGROUND AND RATIONALE 14
1.1.1 Malignant mesothelioma is increasing in the UK 14
1.1.2 Immune checkpoint blockade as molecular target 14
1.1.3 Early signals of efficacy for PD-1 inhibition in mesothelioma 14
1.1.4 PD-L1 blockade in mesothelioma 14
1.2. NIVOLUMAB 14
1.3. PREDICTING PD-1/PD-L1 CHECKPOINT INHIBITION 15
1.4. CTLA-4 IMMUNE CHECKPOINT AND MESOTHELIOMA 15
1.5. PD-L1 AS A PREDICTIVE BIOMARKER 15
1.6. ESTIMATING THE ASSOCIATION BETWEEN MUTATIONAL BURDEN AND NIVOLUMAB EFFICACY 15
1.6.1 Mutational burden predicts the efficacy of PD-1 blockade 15
2 STUDY OBJECTIVES 16
3 STUDY DESIGN 16
3.1. STUDY ENDPOINTS 16
3.1.1 Primary endpoint 16
3.1.2 Secondary endpoints 17
3.1.3 Translational endpoint 17
3.2. DOSE RATIONALE OF NIVOLUMAB 17
3.3. TIMING OF PRIMARY ENDPOINT ANALYSIS 18
4 SELECTION AND ENROLMENT OF PARTICIPANTS 18
4.1. CONSENT 18
4.2. INCLUSION CRITERIA 18
4.3. EXCLUSION CRITERIA 19
4.3.1 Target Disease Exceptions 19
4.3.2 Medical History and Concurrent Diseases 19
4.3.3 Physical and Laboratory Test Findings 20
4.3.4 Allergies and Adverse Drug Reactions 20
4.4. SCREEN FAILURES 20
4.5. REGISTRATION / RANDOMISATION PROCEDURES 20
4.6. CONTRACEPTION 20
5 TRIAL OBSERVATIONS AND PROCEDURES 22
5.1. TRIAL PROCEDURES - SUMMARY 22

5.2. TRIAL PROCEDURES 23
5.2.1 Screening 23
5.2.2 Cycle 1, day 1 24
5.2.3 Day 1 of cycle from cycle 2 (each cycle lasts 14 days) 27
5.3. FOLLOW UP 27
5.3.1 28 (±7) Days Post-Treatment Discontinuation Follow Up 27
5.3.2 Additional Follow Up 28
5.3.3 2 Months Post-Treatment Discontinuation Follow Up (WOCBP ONLY) 28
5.3.4 3 Months Post-Treatment Discontinuation Follow Up 28
5.3.5 4 Months Post-Treatment Discontinuation Follow Up (WOCBP ONLY) 29
5.3.6 5 Months Post-Treatment Discontinuation Follow Up (WOCBP ONLY) 29
5.3.7 6 Months Post-Treatment Discontinuation Follow Up 29
5.3.8 9 Months Post-Treatment Discontinuation Follow Up 29
5.3.9 12 Months Post-Treatment Discontinuation Follow Up 30
5.3.10 Follow Up after 12 Months Post-Treatment Discontinuation 30
5.4. DEVIATIONS AND SERIOUS BREACHES 30
5.5. TRIAL DISCONTINUATION 30
5.5.1 Reasons for trial withdrawal 30
5.6. TREATMENT OF INFUSION RELATED REACTIONS 31
5.7. PROHIBITED AND RESTRICTED THERAPIES DURING THE TRIAL 32
5.7.1 Other Restrictions and Precautions 33
5.7.2 Permitted Therapy 33
5.8. BLINDING AND PROCEDURES FOR UNBLINDING 33
5.8.1 Emergency Unblinding 34
5.8.2 Non-Emergency Unblinding 34
6 TREATMENTS 34
6.1. TREATMENT SCHEDULE 34
6.2. IMP SUPPLY 34
6.2.1 Nivolumab 34
6.2.2 Placebo 35
6.3. ADMINISTRATION 35
6.4. ACCOUNTABILITY 36
6.5. CONCOMITANT MEDICATIONS 36
6.6. DOSE DELAYS AND MODIFICATIONS FOR TOXICITY 36
7 SAFETY 36
7.1. DEFINITIONS 36
7.2. REPORTING WINDOWS 38
7.3. SERIOUSNESS 39
7.4. CAUSALITY 39
7.5. EXPECTEDNESS 39
7.6. REPORTING PROCEDURES 40
7.6.1 Reporting Details 40
7.6.2 Follow up and post-trial SAEs 40
7.6.3 Non-serious AEs 41

7.6.4 Pre-existing Conditions 41
7.6.5 Serious Adverse Events and Reactions 41
7.6.6 Pregnancy 41
7.7. SCTU RESPONSIBILITIES FOR SAFETY REPORTING TO REC 42
7.8. SCTU RESPONSIBILITIES FOR SAFETY REPORTING TO MHRA 42
7.9. SCTU RESPONSIBILITIES FOR SAFETY REPORTING TO BMS 42
8 STATISTICS AND DATA ANALYSES 42
8.1. METHOD OF RANDOMISATION 42
8.2. SAMPLE SIZE 42
8.3. STATISTICAL ANALYSIS PLAN (SAP) 44
8.4. HEALTH ECONOMIC ANAYSIS PLAN 44
8.4.1 Within trial economic analysis 44
8.4.2 Economic modelling 45
9 TRANSLATIONAL ANALYSIS 45
9.1. DIAGNOSTIC BIOPSY OR SURGICAL SAMPLES 45
9.2. PERIPHERAL BLOOD SAMPLES 46
9.3. CONFIRM TRANSLATIONAL RESEARCH 46
9.4. FUTURE ETHICALLY APPROVED TRANSLATIONAL RESEARCH 46
10 REGULATORY 46
10.1. CLINICAL TRIAL AUTHORISATION 46
11 ETHICAL CONSIDERATIONS 46
11.1. ETHICAL APPROVAL 46
11.2. INFORMED CONSENT PROCESS 47
11.3. CONFIDENTIALITY 47
12 SPONSOR 47
12.1. INDEMNITY 47
12.2. FUNDING 47
12.2.1 Site payments 47
12.2.2 Participant payments 47
12.3. AUDITS AND INSPECTIONS 47
13 TRIAL OVERSIGHT GROUPS 48
13.1. TRIAL MANAGEMENT GROUP (TMG) 48
13.2. TRIAL STEERING COMMITTEE (TSC) 48
13.3. INDEPENDENT DATA MONITORING COMMITTEE (IDMC) 48
14 DATA MANAGEMENT 48
14.1. DATA SHARING REQUESTS FOR RESULTS THAT ARE AVAILABLE IN THE PUBLIC DOMAIN 49
15 MONITORING 49
15.1. CENTRAL MONITORING 49

15.2. CLINICAL SITE MONITORING 49
15.2.1 Source Data Verification 50
15.3. SOURCE DATA 50
16 RECORD RETENTION AND ARCHIVING 50
17 PUBLICATION POLICY 50
18 REFERENCES 51
19 SUMMARY OF SIGNIFICANT CHANGES TO THE PROTOCOL 53
APPENDIX 1 ECOG PERFORMANCE STATUS 54
APPENDIX 2 RECIST CRITERIA 55
APPENDIX 3 CTCAE VERSION 4.03 56
APPENDIX 4 SAFETY MANAGEMENT ALGORITHMS 57

LIST OF ABBREVIATIONS

AACR	American Association of Cancer Research
AE	Adverse Event
ALT	Alanine aminotransferase
AR	Adverse Reaction
AST	Aspartate aminotransferase
BMS	Bristol-Myers Squibb
BP	Blood pressure
BUN	Blood urea nitrogen
CI	Chief Investigator
CNS	Central nervous system
CRF	Case Report Form
CTA	Clinical Trial Authorisation
CTCAE	Common Terminology Criteria for Adverse Events
DILI	Drug Induced Liver Injury
DMP	Data Management Plan
DSUR	Development Safety Update Reports
ECOG	Eastern Cooperative Oncology Group
EQ-5D-5L	European Quality of Life-5 Dimensions-5 Levels
FFPE	Formalin-fixed, paraffin-embedded
GCP	Good Clinical Practice
HR	Hazard Ratio
IASLC	International Association for the Study of Lung Cancer
IB	Investigator’s Brochure
IDMC	Independent data monitoring committee
IMP	Investigational Medicinal Product
ISF	Investigator Site File
LDH	Lactate dehydrogenase
MHRA	Medicines and Healthcare products Regulatory Agency
NCI	National Cancer Institute
NSCLC	Non-small cell lung cancer
ORR	Objective response rate
OS	Overall survival
PD-1	Programmed cell death 1
PD-L1	Programmed cell death Ligand 1
PR	Partial response
PFS	Progression-free survival
Q2W	Every two weeks
QALY	Quality adjusted life year
REC	Research Ethics Committee
RECIST	Response Evaluation Criteria In Solid Tumors
RR	Respiratory rate
SAE	Serious Adverse Event
SAP	Statistical Analysis Plan
SAR	Serious Adverse Reaction
SCNA	Somatic copy number alteration
SCTU	Southampton Clinical Trials Unit
SUSAR	Suspected Unexpected Serious Adverse Reaction
TMF	Trial Master File

TMG	Trial Management Group
TSC	Trial Steering Committee
TSH	Thyroid-stimulating hormone
T3	Triiodothyronine
T4	Thyroxine
UAR	Unexpected Adverse Reaction
ULN	Upper limits of normal
WOCBP	Women of childbearing potential

Keywords
Mesothelioma; anti-PD-1; Nivolumab; immunotherapy; RECIST; quality of life; survival; immune checkpoint inhibition; PD-L1

TRIAL SYNOPSIS

**Short title:**	CONFIRM
**Full title:**	Checkpoint blockade for inhibition of relapsed mesothelioma: A phase III trial to evaluate the efficacy of nivolumab in relapsed mesothelioma

**Phase:**	III
**Population:**	Mesothelioma patients (any subtype, pleural or peritoneal) who have undergone prior treatment with at least one line of therapy.
**Primary Objective:**	To compare overall survival (OS) of nivolumab with placebo in patients with relapsed mesothelioma.<br><br>and<br><br>To compare progression-free survival (PFS) of nivolumab with placebo (co-primary endpoint)
**Secondary Objectives:**	To compare objective response rate (ORR), as determined by investigator, of nivolumab to placebo<br>To determine the safety profile of nivolumab in relapsed mesothelioma patients<br>To determine whether nivolumab results in acceptable quality of life and cost per quality adjusted life year (QALY)
**Translational Objectives:**	To define the association of PD-L1 status and OS in patients with relapsed mesothelioma<br>To correlate:<br>a) mutation burden with OS<br>b) immunosuppressive landscape (immune checkpoint expression and infiltration of immune cell) and nivolumab efficacy
**Rationale:**	Effective therapy for relapsed mesothelioma is an unmet need. Despite a significant number of clinical studies in the second line setting, no randomised study to date has been positive. The James Lind Alliance Priority Setting Partnership funded by the NIHR has identified immunotherapy as the number one UK research priority. To date there have been no placebo controlled randomised trials for mesothelioma using PD-L1 or PD-1 checkpoint inhibition.<br>Early promising signals of activity relating to both PD-L1 and PD-1 targeted treatment in mesothelioma implicate a dependency of mesothelioma on this immune checkpoint, and support the development of a randomised phase III trial to evaluate the efficacy of nivolumab. CONFIRM will be the first ever placebo controlled, randomised phase III trial of a PD-1 immune checkpoint inhibitor.<br>PD-1 checkpoint inhibition has revolutionised the treatment of melanoma and is now standard of care in non-small cell lung cancer, squamous cell cancer head and neck and classical Hodgkin's lymphoma. It is being assessed rigorously in numerous other cancers making its evaluation in mesothelioma timely in CONFIRM.
**Trial Design:**	A double blind randomised phase III trial comparing nivolumab (anti-PD-1 antibody) monotherapy versus placebo for one year. The treatment allocation ratio will be 2:1 (treatment: placebo).

**Sample size:**	336 (224 active treatment; 112 placebo)
**Investigational Medicinal Product:**	Nivolumab or matched placebo
**Dosage Regimen / Duration of Treatment:**	Nivolumab 240mg flat dose (or placebo) Q2W over 30 minutes IV until disease progression, to a maximum of 12 months

**URL for Database:**	https://www.imedidata.com
**URL for randomisation:**	https://prod.tenalea.net/ciru/DM/DELogin.aspx
**Primary Trial Endpoints:**	• OS (time from randomisation to death)<br>• PFS (time from randomisation to progression/death)
**Secondary Trial Endpoints:**	• ORR<br>• QoL (EQ-5D-5L)<br>• Toxicity (CTCAE v4.03)<br>• Treatment compliance<br>• Cost-effectiveness (health resource use questionnaire)
**Translational Trial Endpoints:**	• Subgroup analysis of response to nivolumab according to expression of PD-L1 (<1%, 1-49%, ≥50%)<br>• Correlation of mutational burden and immune gene regulation profile with OS
**Total Number of Sites:**	Approximately 25 secondary care

TRIAL SCHEMA

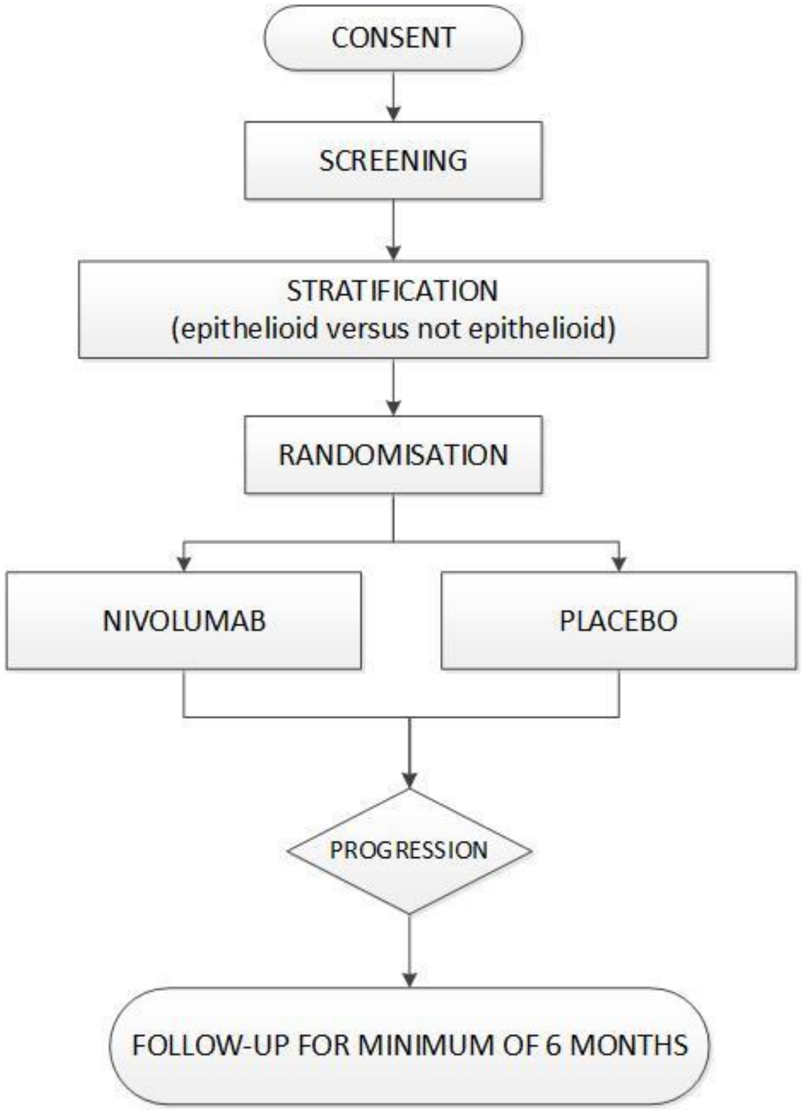

All patients will be registered with NHS Digital to monitor overall survival.

SCHEDULE OF OBSERVATIONS AND PROCEDURES

Visit:	Screening: ≤ 28 days prior to day 1 cycle 1	Baseline: Day 1 cycle 1 Assessments ≤ 3 working days prior to infusion	From cycle 2 until disease progression: day 1 of cycle	28 ±7 days post treatment discontinuation	Post Treatment Discontinuation
Informed Consent	X				
Eligibility Evaluation	X				
Medical History	X				
Physical Exam *(height and weight)*, Vital Signs *(temperature, blood pressure, heart rate, respiratory rate, oxygen saturation)*, ECOG performance status	X	X[a]	X[a]	X[a]	
Concomitant Medication Record	X[q]	X	X	X	
Pregnancy Test in WOCBP	X	X	X[b]	X	X[b]
CT[c] with Modified RECIST (or RECIST 1.1)[d]	X		X[e]	X[t]	
Serum Chemistry *(BUN or serum urea, serum creatinine, Na, K, Ca, glucose, LDH, phosphate and amylase or lipase)*	X	X[f]	X[g]	X	
Full Blood Count *(WBC, lymphocyte count, ANC, haemoglobin, haematocrit, and platelet count)*	X	X[f]	X[g]	X	
Liver Function Tests *(AST or ALT, ALP, total bilirubin, albumin)*	X	X[f]	X[g]	X	
Thyroid Function Tests *(TSH and Free T3 or Free T4)*	X		X[g,h]	X	
C-Reactive Protein		X[f]			
**RANDOMISATION**		X[i]			
Adverse Events	X[r]	X	X	X	X[j]
Completion of NHS Digital Flagging Form		X			
QoL (EQ-5D-5L) and health resource use		X	X[k]	X	X[l]
Record details of next treatment				X	X
**TREATMENTS (nivolumab/placebo)**		X	X[m]		
Archival/Fresh (FFPE) translational tissue (mandatory)	X[s]			X[n]	
Translational blood[o]		X		X	
Survival Status					X[p]

NB: The Participant/legal representative is free to withdraw consent at any time without providing a reason. When withdrawn, the participant will continue to receive standard clinical care. Follow up data will continue to be collected (unless the participant/legal representative has specifically stated that they do not want this to happen).

a = Targeted physical exam as clinically required

b = During treatment on odd numbered cycles only (i.e. every 4 weeks (NB this should be prior to treatment, so if treatment is delayed, test to be performed within 1 day prior to odd number cycle dosing). Result within 1 day prior to dose. Post nivolumab/placebo discontinuation every month for 5 months

c = Chest and abdomen for all participants; pelvis for patients with peritoneal mesothelioma only; and all other known sites of disease

d = Modified RECIST should be used for pleural mesothelioma. For non-pleural mesothelioma, or where measurements for mRECIST cannot be obtained, RECIST 1.1 should be used

e = At week 6 (±3 days from the date of randomisation) and week 12 (±3 days from the date of randomisation) (regardless of the number of cycles of treatment the patient has received and whether trial treatment has stopped early for reasons other than progression)

f= If screening is within three working days of first treatment, assessments do not need to be repeated

g = Samples can be taken ≤3 working days prior to dosing

h = TFTs every 3 cycles: cycles 4, 7, 10…

i = Within 48 hours prior to treatment

j = All AEs to be reported for 100 days post treatment discontinuation and for ongoing drug-related AEs until resolved, return to baseline or deemed irreversible

k = At cycle 4 and cycle 7

l = EQ-5D-5L only at months 6 & 12 post treatment discontinuation

m = Every 14 ±2 days

n = Optional

o = Processed to extract serum and plasma, see Lab Manual for further details

p = Assess every 12 weeks

q = Baseline concomitant medication review within 14 days prior to first dose

r = AEs to be reported from consent

s = Retrieval of archival sample or re-biopsy if sample not available. Only to be performed following consent (mandatory sample)

t = Post-treatment CT scan, as per local policy

1 INTRODUCTION

1.1. BACKGROUND AND RATIONALE

1.1.1 *Malignant mesothelioma is increasing in the UK*

Mesothelioma is an incurable, apoptosis-resistant cancer caused in most cases by previous exposure to asbestos and is increasing in incidence in the UK and beyond [1, 2]. The majority of patients with mesothelioma present with advanced disease and prognosis is poor. Mesothelioma therefore represents a growing health burden, but it remains under-researched and treatment options are limited. Chemotherapy is currently the standard of care in the first-line setting in which two positive randomised phase III trials have been reported, showing improved survival with the addition of pemetrexed or raltitrexed to cisplatin respectively [3, 4]. The recent French MAPS trial has shown that the addition of bevacizumab to pemetrexed-cisplatin and bevacizumab maintenance, improves survival from 16.1 months within the control arm to 18.8 months with the addition of bevacizumab [5].

1.1.2 *Immune checkpoint blockade as molecular target*

The landscape of cancer therapy has been recently transformed by the emergence of immunotherapy involving the targeting of immune checkpoints [6-8]. Programmed cell death 1 (PD-1) is a 55kDa transmembrane inhibitory immunoreceptor expressed by activated T cells that negatively regulates immune responses required for peripheral self-tolerance. PD-1 interacts with its ligand PD-L1, a member of the B7 gene family, which is expressed on mesothelioma cells [9, 10]. The expression of PD-L1 (≥5% positively stained cells) has been reported in 40% of mesothelioma overall, with a higher rate in sarcomatoid mesotheliomas and is a poor prognostic factor. The PD-1/PD-L1 axis mediates an inhibitory signal to T cells leading to induction of apoptosis via PD-1 activation. Accordingly, PD-1 or PD-L1 blockade de- represses T cell activation, unleashing a clinical immune response with tumour regression [11].

1.1.3 *Early signals of efficacy for PD-1 inhibition in mesothelioma*

Targeting the PD-1/PD-L1 in mesothelioma has demonstrated promising efficacy. Of 25 patients receiving pembrolizumab in a single arm phase I/II study (Keynote 28 [KN028]), the objective response rate was 20% in patients with PD-L1 positive malignant pleural mesothelioma (≥1% PD-L1 positive tumour cells by immunohistochemistry). Additionally, 52% of patients had stable disease, resulting in a disease control rate of 72% [12].

1.1.4 *PD-L1 blockade in mesothelioma*

PD-L1 blockade has also demonstrated promising efficacy in patients with mesothelioma [13]. In a phase 1b study (NCT01772004), 53 patients were treated with avelumab (MSB0010718C, Merck Serono), with histologically or cytologically confirmed unresectable mesothelioma (pleural or peritoneal) that progressed after prior platinum-pemetrexed–containing regimen or platinum-based regimen followed by pemetrexed. Avelumab was administered at a dose of 10mg/kg as a 1-h infusion Q2W until confirmed progression, unacceptable toxicity, or any criteria for withdrawal occurred. Patients had received a median of 1.5 prior treatments (range, 07.4). Histology was epithelial (81.1%), mixed (11.3%), or sarcomatoid (3.8%). Objective responses were observed in five (9.4%) patients; all were partial responses and durable. Stable disease (SD) was observed in nine additional patients (45%). The overall disease control rate (Partial Response [PR] plus SD) was 56.6% (30 patients). Median progression free survival (PFS) by RECIST was 17.1 weeks (95% CI: 6.1, 30.1), and the PFS rate at 24 weeks was 38.4% (95% CI: 23.3, 53.4).

1.2. NIVOLUMAB

Nivolumab is a human IgG4 anti-PD-1 monoclonal antibody which blocks PD-1 receptor on activated T-cells, which has been approved by the Food and Drug Administration for treatment of patients with

unresectable or metastatic melanoma unresponsive to other drugs, and relapsed non-small cell lung cancer (NSCLC), recurrent renal cancer (RCC) and Classic Hodgkin lymphoma (cHL). In a phase IIA clinical trial of nivolumab (3mg/kg q2w) conducted at NKI, Amsterdam, the disease control rate at 12 weeks was 50% (n=34) [14].

1.3. PREDICTING PD-1/PD-L1 CHECKPOINT INHIBITION

PD-L1 has been implicated as a putative biomarker of efficacy for PD-L1 and PD-1 inhibitors from correlative analysis of several clinical studies [8, 15]; however, the association between PD-L1 expression and efficacy in mesothelioma remains controversial. In mesothelioma, study Keynote 28 failed to demonstrate a clear association between the level of PD-L1 expression and efficacy. PD-L1 expression was also not found to be correlated with efficacy in the positive study of nivolumab in squamous NSCLC (checkmate 017) [16]. Mutation burden is associated with increased neo-antigen load and sensitivity to PD-1 blockade [17]. We, and others, have shown that a subset of mesotheliomas exhibit significant genomic instability and high mutational burden, with shorter survival [18]; however, neither the correlation with expression of PD-L1 nor response to PD-L1/PD-1 inhibition has been explored.

1.4. CTLA-4 IMMUNE CHECKPOINT AND MESOTHELIOMA

Cytotoxic T-lymphocyte-associated protein 4 (CTLA-4) represents a second immune checkpoint on T cells, which interacts with CD80/CD86 on antigen presenting cells. A recent phase II trial demonstrated activity of the CTLA-4 inhibitor tremelimumab in mesothelioma [19]; results from a phase III clinical trial, DETERMINE (NCT01843374) were negative (Kindler et al, ASCO 2016). Accordingly, there remains no agreed standard of care in the relapsed setting.

1.5.PD-L1 AS A PREDICTIVE BIOMARKER

We will conduct PD-L1 evaluation as a potential predictive correlate of overall survival. Genome-wide copy number analysis will be conducted using molecular inversion probe array. Using a custom bioinformatics workflow developed in Leicester, predictive copy number variations which associate with minimum hazard ratio will be identifiable and their prognostic interactions interrogated. RNA will be extracted to support transcriptome analysis to profile the immunophenotype. 100% tissue block collection will be achieved through mandatory inclusion of fresh or archival tissue (obtained or retrieved after consent) in the inclusion criteria for this study.

1.6.ESTIMATING THE ASSOCIATION BETWEEN MUTATIONAL BURDEN AND NIVOLUMAB EFFICACY

1.6.1 *Mutational burden predicts the efficacy of PD-1 blockade*

A recent report in *Science* by Rizvi et al, [17] showed the interaction between mutation burden and PD-1 inhibitor efficacy. Similarly, evidence from patients harbouring colorectal tumours with DNA mismatch repair has been shown to predict the efficacy of PD-1 blockade [31-33]. Collectively, these studies confirm that genomic instability coupled to higher mutation load, is associated with neo antigen expression and upregulated immune escape mechanisms.

2 STUDY OBJECTIVES

	Objective	Endpoint used to evaluate
**Primary:**	To determine whether nivolumab increases overall survival in relapsed mesothelioma patients<br><br>and<br><br>To determine whether nivolumab increases progression-free survival (PFS) in relapsed mesothelioma patients	Overall survival (time from randomisation to death)<br><br><br><br>Progression-free survival (time from randomisation to progression/death)
**Secondary:**	To determine whether nivolumab:<br>a) increases response rate (RR)<br>b) has good safety/tolerability<br>c) results in acceptable patient quality of life and cost per QALY	 a) modified RECIST or RECIST 1.1<br>b) CTCAE v4.03<br>c) EQ-5D-5L and health resource usage
**Translational:**	Subgroup analysis of response to nivolumab according to expression of PD-L1 (<1%, 1-49%, ≥50%)<br><br>To determine if mutational burden and immune gene regulation profile correlate with overall survival	Expression of PD-L1 will be determined by immunohistochemistry and sensitivity to nivolumab assessed by group<br>Mutational burden will be determined using array based somatic copy number alteration (SCNA) analysis; immune gene regulation profile will be determined by transcriptomic analysis.

3 STUDY DESIGN

A double blind, placebo controlled randomised phase III trial comparing nivolumab (anti PD-1 antibody) monotherapy 240mg Q2W versus placebo until disease progression, for a maximum of 12 months. The treatment allocation ratio will be 2:1 in favour of nivolumab.

3.1. STUDY ENDPOINTS

3.1.1 *Primary endpoint*

Overall survival defined as time from randomisation to death from any cause.

Progression-free survival defined as time from randomisation to progression (according to modified RECIST or RECIST 1.1), or death from any cause (whichever event comes first). Modified RECIST or RECIST 1.1 will be assessed at week 6 (±3 days) and week 12 (±3 days) (regardless of the number of cycles of treatment the patient has received and whether trial treatment has stopped early for reasons other than progression).

Additionally, patients will be flagged with the NHS Digital to reduce loss at follow up. Regular CT scans will not be mandated. Follow-up imaging will be as per local hospital policy and as clinically indicated.

3.1.2 *Secondary endpoints*

Toxicity will be assessed using CTCAE v4.03 at baseline, after each treatment cycle, and for 100 days post treatment discontinuation and for ongoing drug-related AEs until resolved, return to baseline or deemed irreversible.

Quality of life and health economic data will be collected using EQ-5D-5L plus data on health resource usage collected at baseline, after cycles 3 and 6 and at 28 days post treatment discontinuation. EQ-5D-5L will also be collected at 6, and 12 months post treatment discontinuation.

3.1.3 *Translational endpoint*

Subgroup analysis of response to nivolumab according to expression of PD-L1: PD-L1 expression (<1%, 1-49%, ≥50%), will be determined by immunohistochemistry and sensitivity to nivolumab assessed by group.

Correlation between mutational burden and immune gene regulation profile and overall survival: this will be determined using array based SCNA analysis and transcriptomic analysis.

3.2. DOSE RATIONALE OF NIVOLUMAB

The nivolumab dose of 240 mg every 2 weeks (Q2W) was selected based on clinical data and modelling and simulation approaches using population pharmacokinetics (PPK) and exposure-response analyses of data from studies in multiple tumour types (melanoma, NSCLC, and renal cell carcinoma [RCC]) where body weight normalised dosing (mg/kg) has been used. PPK analyses have shown that the pharmacokinetics (PK) of nivolumab is linear with proportional exposure over a dose range of 0.1 to 10 mg/kg, and no differences in PK across ethnicities and tumour types were observed. Nivolumab clearance and volume of distribution were found to increase as the body weight increases, but less than the proportional increase with increasing weight, indicating that mg/kg dosing represents an over-adjustment for the effect of body weight on nivolumab PK.

The PPK model previously developed using data from NSCLC subjects has recently been updated, using data from 1544 subjects from 7 studies investigating nivolumab in the treatment of melanoma, NSCLC, and RCC. In this dataset, the median (minimum - maximum) weight was 77 kg (35 kg - 160 kg) and thus, an approximately equivalent dose of 3 mg/kg for an 80 kg subject, nivolumab 240 mg Q2W was selected for future studies. To predict relevant summary exposures of nivolumab 240 mg Q2W, the PPK model was used to simulate nivolumab 3 mg/kg Q2W and 240 mg Q2W. In the simulations, the simulated patient populations consisted of 1000 subjects per treatment arm randomly sampled from the aforementioned pooled database of cancer subjects. Because no differences in PK were noted across ethnicities and tumour types, these simulated melanoma and NSCLC data will be applicable to subjects with other tumour types. The simulated measure of exposure of interest, time-averaged concentrations (Cavgss) for 240 mg Q2W are predicted to be similar for all subjects in reference to 80 kg subjects receiving 3 mg/kg Q2W.

Nivolumab is safe and well tolerated up to 10 mg/kg Q2W dose level. Adverse events have been broadly consistent across tumour types following monotherapy and have not demonstrated clear dose-response or exposure-response relationships. Additionally, the simulated median and 95th prediction interval of nivolumab summary exposures across body weight range (35 - 160 kg) are predicted to be maintained below the corresponding observed highest exposure experienced in nivolumab i.e., 95th percentile following nivolumab 10 mg/kg Q2W from clinical study CA 209-003. Thus, while subjects in the lower body weight ranges would have greater exposures than 80 kg subjects, the exposures are predicted to be within the range of observed exposures at doses (up to 10 mg/kg Q2W) used in the nivolumab clinical program, and are not considered to put subjects at increased risk. For subjects with greater body weights, the simulated ranges of exposures are also not expected to affect efficacy,

because the exposures predicted following administration of a 240 mg Q2W are on the flat part of the exposure-response curves for previously investigated tumours, melanoma and NSCLC. Given the similarity of nivolumab PK across tumour types and the similar exposures predicted following administration of 240 mg flat dose compared to 3 mg/kg, it is expected that the safety and efficacy profile of 240 mg nivolumab will be similar to that of 3 mg/kg nivolumab. Thus, nivolumab 240 mg every 2 weeks over 30 minutes will be used in this study.

3.3. TIMING OF PRIMARY ENDPOINT ANALYSIS

The primary endpoint analysis will occur when 291 death events have been reported, or once the TSC advise that follow-up should end (for example, in the case that it is anticipated the required number of events will not be reached in the timescales of the study). All patients will be consented to allow linkage with NHS Digital data to obtain overall survival and progression-free survival data.

4 SELECTION AND ENROLMENT OF PARTICIPANTS

4.1. CONSENT

Consent to enter the trial must be sought from each participant only after a full explanation has been given, an information leaflet offered and time allowed for consideration. Signed participant consent should be obtained prior to undertaking any trial-related procedures. The right of the participant to refuse to participate without giving reasons must be respected. After the participant has entered the trial the clinician remains free to give alternative treatment to that specified in the protocol at any stage if he/she feels it is in the participant's best interest, but the reasons for doing so should be recorded. In these cases, the participants remain within the trial for the purposes of follow-up and data analysis. All participants are free to withdraw at any time from the protocol treatment without giving reasons and without prejudicing further treatment.

Consent forms will be sent to SCTU following randomisation. The consent form will be scanned and sent **from an nhs.net email address** to uhs.sctu@nhs.net marked FAO CONFIRM trial team. The original signed consent form will be filed in the investigator site file.

4.2. INCLUSION CRITERIA

- Patients must have signed and dated a REC-approved written informed consent form in accordance with regulatory and institutional guidelines. This must be obtained before the performance of any protocol-related procedures that are not part of normal patient care.
- Patients must be willing and able to comply with scheduled visits, treatment schedule, laboratory tests, and other requirements of the study.
- Histological confirmation of mesothelioma (any subtype, pleural or peritoneal).
- Patients must have received at least one prior line of treatment.

  Prior lines of antineoplastic therapy, including chemotherapy, surgical resection of lesions, radiation therapy, must be completed at least 14 days prior to receiving study treatment.
- Eastern Cooperative Oncology Group Performance Status (ECOG PS) 0-1 (see appendix 1).
- Radiologically assessable disease by modified RECIST (pleural mesothelioma) or RECIST 1.1 (non-pleural mesothelioma or where measurements for mRECIST cannot be obtained).
- Evidence of disease progression by CT scan (performed within 28 days prior to first dose of nivolumab/placebo).
- Prior palliative radiotherapy must have been completed at least 14 days prior to study drug administration.
- Consent to provide mandatory baseline tissue and blood samples for research.
- Age ≥18 years.

- Screening laboratory values must meet the following criteria within 48 hours prior to commencement of treatment:
  i) White blood cells ≥ 2 x 10^9/L
  ii) Neutrophils ≥ 1.5 x 10^9/L
  iii) Platelets ≥ 100 x 10^9/L
  iv) Haemoglobin ≥ 90 g/L
  v) Serum creatinine of ≤ 1.5 X ULN or creatinine clearance (CrCl) > 50 mL/minute (using Cockcroft/Gault formula)
    – Female CrCl= [(140 - age in years) x weight in kg x 0.85) ÷ (72 x serum creatinine in µmol/L)]
    – Male CrCl= [(140 - age in years) x weight in kg x 1.00) ÷ (72 x serum creatinine in µmol/L)]
  vi) AST ≤ 3 x ULN OR ALT ≤ 3 x ULN (if both are assessed, both need to be ≤ 3 x ULN)
  vii) Total bilirubin ≤ 1.5 x ULN (except patients with Gilbert Syndrome, who must have total bilirubin < 51.3 µmol/L)
- Reproductive status (refer to section 4.6)
  a) Women of childbearing potential (WOCBP, as defined in section 4.6) must have a negative serum or urine pregnancy test (minimum sensitivity 25 IU/L or equivalent units of Human Chorionic Gonadotropin) at enrolment and within 24 hours prior to the start of study treatment. An extension up to 3 days prior to start of study treatment may be permissible in situations where results cannot be obtained within a 24-hour window.
  b) Women must not be breastfeeding.
  c) WOCBP must agree to use a highly effective method of contraception (as outlined in section 4.6) for the duration of treatment and 5 months after the last dose of nivolumab/placebo.
  d) Men who are sexually active with WOCBP must use the contraceptive methods as outlined in section 4.6 for the duration of treatment and for 7 months after the last dose of nivolumab/placebo.
- Expected survival of at least 12 weeks.

4.3. EXCLUSION CRITERIA

4.3.1 *Target Disease Exceptions*

a) Patients with untreated, symptomatic central nervous system (CNS) metastases are excluded. Participants are eligible if CNS metastases are adequately treated and participants are neurologically returned to baseline (except for residual signs or symptoms related to the CNS treatment) for at least 2 weeks prior to treatment assignment. In addition, participants must be either off corticosteroids, or on a stable or decreasing dose of ≤10 mg daily prednisone (or equivalent) for at least 2 weeks prior to treatment.

b) Patients with carcinomatous meningitis are excluded.

4.3.2 *Medical History and Concurrent Diseases*

a) Patients with active, known or suspected autoimmune disease. Patients with Type I diabetes mellitus, residual hypothyroidism due to an autoimmune condition requiring hormone replacement, psoriasis not requiring systemic treatment, or conditions not expected to recur in the absence of an external trigger are permitted to enrol.
b) Patients with a condition requiring systemic treatment with either corticosteroids (> 10 mg daily prednisone equivalent) or other immunosuppressive medications within 14 days of the first dose of study drug administration. Inhaled or topical steroids and adrenal replacement steroid doses > 10 mg daily prednisone or equivalent are permitted in the absence of active autoimmune disease.

c) Other active malignancy requiring concurrent intervention.
d) Patients with previous malignancies (except non-melanoma skin cancers, and the following in situ cancers: bladder, gastric, colon, endometrial, cervical/dysplasia, melanoma or breast) are excluded unless a complete remission was achieved at least 2 years prior to study entry AND no additional therapy is required during the study period.
e) Any serious or uncontrolled medical disorder or active infection that, in the opinion of the investigator, may increase the risk associated with study participation, study drug administration, or would impair the ability of the patient to receive protocol therapy.
f) All toxicities attributed to prior anti-cancer therapy other than alopecia and fatigue not resolved to Grade 1 (NCI CTCAE version 4.03) or baseline before administration of study drug.
g) Patients who have not recovered from the effects of major surgery or significant traumatic injury at least 14 days before the first dose of study treatment.
h) Known alcohol or drug abuse.
i) Patients who have received prior therapy with anti-PD-1, anti-PD-L1, anti-PD-L2, anti- CD137, or anti-CTLA-4 antibody (including ipilimumab or any other antibody or drug specifically targeting T-cell co-stimulation or checkpoint pathways) or who have previously taken part in either a randomised Bristol Myers Squibb (BMS) clinical trial for nivolumab or ipilimumab including study CA209-743 (CheckMate 172) or in the CCTG trial of pembrolizumab (IND.227).

4.3.3 *Physical and Laboratory Test Findings*

a) Known history of testing positive for human immunodeficiency virus (HIV) or known acquired immunodeficiency syndrome (AIDS).
b) Any positive test for hepatitis B virus or hepatitis C virus indicating acute or chronic infection.

4.3.4 *Allergies and Adverse Drug Reactions*

a) History of severe hypersensitivity reactions to other monoclonal antibodies

4.4. SCREEN FAILURES

Patients who are approached and decline or fail screening will have their initials, year of birth and reasons for not entering the study recorded on a screening form.

4.5. REGISTRATION / RANDOMISATION PROCEDURES

Patients must be registered on the trial specific database. Eligibility must be confirmed and ideally the Eligibility form on Rave should be completed prior to randomisation. It is recommended to not complete the Eligibility form on Rave until eligibility is confirmed. Randomisation via interactive web response system (IWRS) will occur after all screening procedures have been carried out and the eCRF has been completed. **Randomisation must take place within 48 hours prior to treatment commencement.** Patients will be stratified according to epithelioid vs. non-epithelioid. Unblinded pharmacy staff will obtain treatment assignment from the IWRS using patient information provided by the investigator on a trial randomisation form.

4.6. CONTRACEPTION

Definitions of women of childbearing potential (WOCBP) and fertile men:

A woman of childbearing potential (WOCBP) is a sexually mature woman (i.e. any female who has experienced menstrual bleeding) who has not:

- Undergone a hysterectomy or bilateral oophorectomy/salpingectomy

- Been postmenopausal for 12 consecutive months (i.e. who has had menses at any time in the preceding 12 consecutive months without an alternative medical cause)
- Had premature ovarian failure confirmed by a specialist gynaecologist

A man is considered fertile after puberty unless permanently sterile by bilateral orchiectomy.

Female patients

To be considered eligible for the trial, all female patients who are WOCBP must consent to use one of the following methods of highly effective contraception from the first administration of study treatment, throughout the trial and for 5 months after last dose of study treatment:

- Combined (estrogen and progestogen containing) hormonal contraception associated with inhibition of ovulation[1]:
  - Oral
  - Intravaginal
  - Transdermal
- Progestogen-only hormonal contraception associated with inhibition of ovulation[1]:
  - Oral
  - Injectable
  - Implantable[2]
- Intrauterine device (IUD)[2]
- Intrauterine hormone-releasing system (IUS)[2]
- Bilateral tubal occlusion[2]
- Vasectomised partner[2,3]
- Sexual abstinence[4]

[1]Hormonal contraception may be susceptible to interaction with the IMP, which may reduce the efficacy of the contraception method.

[2]Contraception methods that in the context of this guidance are considered to have low user dependency.

[3]Vasectomised partner is a highly effective birth control method provided that partner is the sole sexual partner of the WOCBP trial participant and that the vasectomised partner has received medical assessment of the surgical success.

[4]In the context of this guidance sexual abstinence is considered a highly effective method only if defined as refraining from heterosexual intercourse during the entire period of risk associated with the study treatments. The reliability of sexual abstinence needs to be evaluated in relation to the duration of the clinical trial and the preferred and usual lifestyle of the participant.

Male patients

To be considered eligible for the trial, male patients (including those with partners of child-bearing potential) must consent to use the following methods of contraception from the first administration of study treatment, throughout the trial and for 7 months after last dose of nivolumab/placebo:

- Condom
- Female partner to use one of the highly effective methods of contraception detailed above

Male patients must also refrain from donating sperm during this period.

5 TRIAL OBSERVATIONS AND PROCEDURES

5.1. TRIAL PROCEDURES - SUMMARY

Patients will undergo screening evaluations to determine eligibility prior to first nivolumab/placebo dose. Safety, including adverse event monitoring and physical examination, should be monitored continually, and safety assessments are recommended every 2 weeks while receiving nivolumab/placebo treatment.

The following assessments should be monitored starting on Cycle 1 Day 1 and will continue as per the schedule in the observations and procedures at the specified frequency (see Schedule of Events, page 12):

Safety Assessments

a) Adverse events continuously throughout the study.
b) Physical examination and physical measurements including weight and ECOG PS.
c) Full blood count (FBCs) with differential, including white blood cell (WBC), lymphocyte count, absolute neutrophil count (ANC), haemoglobin, haematocrit, and platelet count. Samples can be taken up to 3 working days prior to dosing. Results to be obtained prior to dosing on infusion days.
d) Serum chemistry tests (blood urea nitrogen [BUN] or serum urea level, serum creatinine, sodium, potassium, calcium, glucose, lactate dehydrogenase [LDH], phosphate, and amylase or lipase). Samples can be taken up to 3 working days prior to dosing. Results to be obtained and reviewed prior to dosing on infusion days.
e) Liver function tests including aspartate aminotransferase [AST] or alanine aminotransferase [ALT], total bilirubin, alkaline phosphatase, and albumin. Samples can be taken up to 3 working days prior to dosing. Results to be obtained and reviewed prior to dosing on infusion days.
f) Thyroid function testing including TSH and free T3/4. Samples can be taken up to 3 working days prior to dosing. Results to be obtained and reviewed prior to dosing on infusion days.

Imaging Assessment for the Study

Any incidental findings of potential clinical relevance that are not directly associated with the objectives of the protocol should be evaluated and handled by the Study Investigator as per standard medical/clinical judgment.

Vital Signs and Physical Examinations

Vital signs including temperature, blood pressure (BP), heart rate , respiratory rate (RR), oxygen saturation by pulse oximetry at rest (also monitor amount of supplemental oxygen if applicable) within 3 days of dosing. Obtain prior to dosing and at any time a patient has any new or worsening respiratory symptoms. If a patient shows changes in oxygen saturation or supplemental oxygen requirement, or other pulmonary-related signs (e.g. hypoxia, fever) or symptoms (e.g. dyspnoea, cough) consistent with possible pulmonary adverse events, the patient should be immediately evaluated to rule out pulmonary toxicity, according to the suspected pulmonary toxicity management algorithm in appendix 4, also contained within the Investigator's Brochure.

Pregnancy Testing

Pregnancy testing must be completed for WOCBP at screening, within 24 hours prior to start of study drug (an extension up to 72 hours prior to start of study drug may be permissible in situations where results cannot be obtained within a 24 hour window), during treatment on odd numbered cycles (i.e. every 4 weeks (NB this should be prior to treatment, so if treatment is delayed, test to be performed within 24 hours prior to odd number cycle dosing)), and every month for 5 months following the end of treatment. Pregnancy testing can be completed more frequently if required by local standards.

Efficacy Assessments

Mandatory tumour assessments are to be done at week 6 (±3 days from the date of randomisation) and week 12 (±3 days from the date of randomisation) (regardless of the number of cycles of treatment the patient has received and whether trial treatment has stopped early for reasons other than progression), and additional tumour assessments should be performed as required by local standards of care or at the investigator's discretion as discussed in the schedule of observations and procedures until:

a) Disease progression per modified RECIST or RECIST 1.1 criteria (See Appendix 2). This should be consistent with RECIST criteria used for disease evaluation at CONFIRM baseline.
b) Patient lost to follow-up
c) Withdrawal of study consent by patient
d) 4.5 years after the first dose of study treatment

Study evaluations will take place in accordance with the schedule of observations and procedures and should be performed according to modified RECIST or RECIST 1.1 criteria, as appropriate to disease subtype.

CT with IV contrast is the preferred imaging modality for assessing radiographic tumour response. If a patient has a known allergy to contrast material, please use local prophylaxis standards to obtain the assessment with contrast if possible. In cases where contrast is strictly contraindicated, a non-contrast scan will suffice. Screening assessments, including chest, abdomen for all patients; pelvis for patients with peritoneal mesothelioma only; and all known or suspected sites of disease, should be performed within 28 days of first dose of study treatment. In addition to chest and abdomen, all known or suspected sites of disease (including CNS) should be assessed at subsequent assessments using the same imaging method and technique.

If more than one method is used at screening, then the most accurate method according to modified RECIST (for pleural mesothelioma) or RECIST 1.1 (for non-pleural mesothelioma or where measurements for mRECIST cannot be obtained) should be used when recording data and should again be used for all subsequent assessments. Bone scan, PET scan, or ultrasound is not adequate for assessment of RECIST response. In selected circumstances where such modalities are the sole modalities used to assess certain non-target organs, those non-target organs may be evaluated less frequently. For example, bone scans may need to be repeated as per local practice only when complete response is identified in the target disease or when progression in bone is suspected. Previously treated CNS metastases are not considered measurable lesions for purposes of RECIST determined response.

Tumour measurements should be made by the same investigator or radiologist for each assessment whenever possible. Changes in tumour measurements and tumour responses to guide ongoing study treatment decisions should be assessed by the investigator using modified RECIST or RECIST 1.1, ensuring that assessment criteria are applied consistently throughout the trial.

Survival will be followed after progression either by direct contact (clinical visits) or patient flagging via NHS Digital (formerly Health and Social Care Information Centre) or equivalent until death, withdrawal of study consent, or conclusion of the study.

5.2. TRIAL PROCEDURES

5.2.1 Screening

All patients will undergo the following assessments at screening within 28 days prior to first trial treatment (unless otherwise specified):

- Medical history (asbestos exposure, smoking history, comorbidities, particularly chronic pulmonary and chronic cardiovascular diseases following international established categories e.g. NYHA for cardiac insufficiency)
- Physical exam (including height and weight)
- Vital signs and oxygen saturation (temperature, BP, heart rate, RR, O_2 saturation by pulse oximetry at rest (also monitor amount of supplement oxygen if applicable).
- ECOG Performance Status
- CT scan including chest, abdomen for all patients; pelvis for patients with peritoneal mesothelioma only; and all other known sites of disease. Patients must have evaluable disease by CT per modified RECIST (pleural mesothelioma) or RECIST 1.1 (non-pleural mesothelioma, or where measurements for mRECIST cannot be obtained).
- Serum biochemistry: blood urea nitrogen (BUN) or serum urea level (SUL), serum creatinine, sodium, potassium, calcium, glucose, lactate dehydrogenase (LDH), phosphate, and amylase or lipase.
- FBC with differential including white blood cell (WBC), lymphocyte count, absolute neutrophil count (ANC), haemoglobin, haematocrit, and platelet count.
- Liver function tests including aspartate aminotransferase [AST] or alanine aminotransferase [ALT], total bilirubin, alkaline phosphatase, and albumin.
- Thyroid function testing including TSH and free T3 or free T4.
- Concomitant medication (within 14 days of first dose)
- Pregnancy test in WOCBP (serum or urine) (within 24 hours of first dose)
- Biopsy if archival tissue is not available
- Adverse events

5.2.2 Cycle 1, day 1

Cycle 1 Day 1, pre-treatment visit should take place within three working days prior to first treatment. If the screening visit is performed within three working days of first treatment, common assessments (marked with an astrix) do not need to be repeated unless clinically indicated.

- Targeted physical exam as clinically required
- Concomitant medication*
- Vital signs and oxygen saturation (temperature, BP, heart rate, RR, O_2 saturation by pulse oximetry at rest (also monitor amount of supplement oxygen if applicable)*
- ECOG Performance Status*
- Serum biochemistry: blood urea nitrogen (BUN) or serum urea level (SUL), serum creatinine, sodium, potassium, calcium, glucose, lactate dehydrogenase (LDH), phosphate, and amylase or lipase. Samples can be taken up to 3 working days prior to dosing*.
- FBC with differential including white blood cell (WBC), lymphocyte count, absolute neutrophil count (ANC), haemoglobin, haematocrit, and platelet count. Samples can be taken up to 3 working days prior to dosing. Results to be obtained prior to dosing on infusion days*.
- Liver function tests including aspartate aminotransferase [AST] or alanine aminotransferase [ALT], total bilirubin, alkaline phosphatase, and albumin. Samples can be taken up to 3 working days prior to dosing. Results to be obtained prior to dosing on infusion days*.
- C-Reactive Protein (CRP)
- Adverse events*
- Pregnancy test in WOCBP
- Translational bloods (1 x 10 ml EDTA tube and 1 x 10 ml vacutainer serum tube processed according to the CONFIRM Lab Manual and stored at -80°C until the end of the study)
- European Quality of Life-5 Dimensions-5 Levels (EQ-5D-5L) and resource use questionnaire

- Randomisation (see figure 1) (within 48 hours prior to first dose)
- Completion of NHS Digital Patient Flagging Form

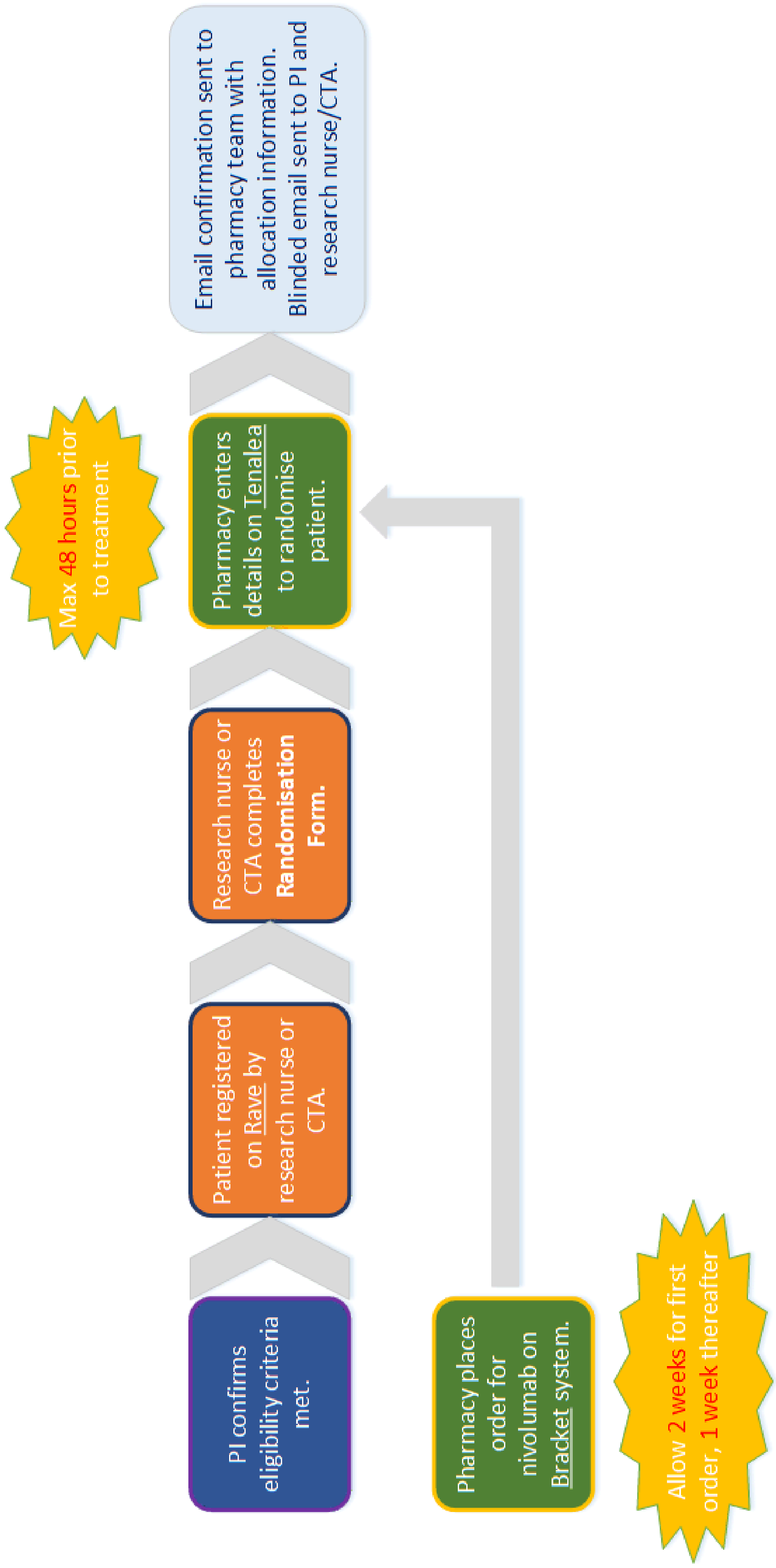

Figure 1: Randomisation procedure

5.2.3 Day 1 of cycle from cycle 2 (each cycle lasts 14 days)

During treatment with nivolumab/placebo patients should be seen within 3 working days prior to D1 of each cycle. At each visit:

- Targeted physical exam (including weight)
- Vital signs and oxygen saturation (temperature, BP, heart rate, RR, O2 saturation by pulse oximetry at rest (also monitor amount of supplement oxygen if applicable)
- Pregnancy test for WOCBP (serum or urine as per local practice within 1 day prior to dose – on odd numbered cycles (i.e. every four weeks ((NB this should be prior to treatment, so if treatment is delayed, test to be performed within 1 day prior to odd number cycle dosing))
- ECOG Performance Status
- CT Scan (at week 6 (±3 days) and week 12 (±3 days), regardless of the number of cycles. To be calculated from the date of randomisation.)
- Disease Evaluation with modified RECIST or RECIST 1.1, consistent with baseline assessment (as per local practice, mandated at weeks 6 and 12, regardless of the number of cycles)
- Serum biochemistry: blood urea nitrogen (BUN) or serum urea level (SUL), serum creatinine, sodium, potassium, calcium, glucose, lactate dehydrogenase (LDH), phosphate, and amylase or lipase. Samples can be taken up to 3 working days prior to dosing. Results to be obtained and reviewed prior to dosing on infusion days.
- FBC with differential including white blood cell (WBC), lymphocyte count, absolute neutrophil count (ANC), haemoglobin, haematocrit, and platelet count. Samples can be taken up to 3 working days prior to dosing. Results to be obtained and reviewed prior to dosing on infusion days.
- Liver function tests including aspartate aminotransferase [AST] or alanine aminotransferase [ALT], total bilirubin, alkaline phosphatase, and albumin. Samples can be taken up to 3 working days prior to dosing. Results to be obtained and reviewed prior to dosing on infusion days.
- Thyroid function testing including TSH and free T3 or free T4 (every 3 cycles: cycle 4, 7…, samples can be taken up to 3 working days prior to dosing). Results to be obtained and reviewed prior to dosing on infusion days.
- Concomitant medication
- Toxicity assessment and adverse events
- EQ-5D-5L and resource use questionnaire (at cycles 4 and 7 only)

5.3. FOLLOW UP

Post-treatment follow-up is of critical importance and is essential to preserving patient safety and the integrity of the study. Patients will be followed up for a minimum of six months post progression or treatment discontinuation.

5.3.1 28 (±7) Days Post-Treatment Discontinuation Follow Up

At 28 (±7) days post treatment discontinuation the following procedures will be carried out:

- Physical exam
- Vital signs and oxygen saturation (temperature, BP, heart rate, RR, O_2 saturation by pulse oximetry at rest, also monitor amount of supplement oxygen if applicable)
- Serum biochemistry: blood urea nitrogen (BUN) or serum urea level (SUL), serum creatinine, sodium, potassium, calcium, glucose, lactate dehydrogenase (LDH), phosphate, and amylase or lipase.
- FBC with differential including white blood cell (WBC), lymphocyte count, absolute neutrophil count (ANC), haemoglobin, haematocrit, and platelet count
- Liver function tests including aspartate aminotransferase [AST] or alanine aminotransferase [ALT], total bilirubin, alkaline phosphatase, and albumin
- Thyroid function testing including TSH and free T3 or free T4
- Concomitant medication

- Translational bloods (mandatory – 1 x 10 ml EDTA tube and 1 x 10 ml vacutainer serum tube processed according to the CONFIRM Lab Manual and stored at -80°C until the end of the study)
- FFPE tissue biopsy (optional)
- CT Scan (as per local practice)
- Disease evaluation with modified RECIST or RECIST 1.1 (consistent with baseline assessment)
- EQ-5D-5L and resource use questionnaire
- Record details of next treatment
- Adverse events
- Pregnancy testing in WOCBP (serum or urine)

5.3.2 Additional Follow Up

Patients should be followed up for a minimum of 6 months following treatment discontinuation at the following time intervals. These visits are calculated from the last dose of IMP/placebo and not from the date of previous follow up.

Follow up data may be collected from a combination of physical visits, patient notes and where appropriate via telephone. The only exceptions to this are the first 5 months follow up for WOCBP where a pregnancy test must be performed.

5.3.3 *2 Months Post-Treatment Discontinuation Follow Up (WOCBP ONLY)*

This visit is **only applicable for WOCBP** and should occur 60 (±7) days after post-treatment discontinuation.

The following data should be obtained:

- Adverse events.
- CT Scans – Where a patient has undergone a routine CT scan, record this in the Ad Hoc CT scan folder on the eCRF.
- Additional therapy – Where a patient has received new cancer treatment outside of the trial, record this on the Post Study Therapy form on the eCRF.
- Pregnancy Testing (serum or urine). Please document the results and record this on the Pregnancy Test (logform) on the eCRF. A pregnancy notification form should be submitted if required.

5.3.4 *3 Months Post-Treatment Discontinuation Follow Up*

This visit is applicable for all trial participants and should occur 90 (±14) days post-treatment discontinuation.

The following data should be obtained:

- Adverse events.
- CT Scans – Where a patient has undergone a routine CT scan, record this in the Ad Hoc CT scan folder on the eCRF if the patient has already had their week 12 scan or record this in the Week 12 CT scan folder if the CT scan coincides with the Week 12 (±3 days from the date of randomisation) time-point.
- Additional therapy – Where a patient has received new cancer treatment outside of the trial, record this on the Post Study Therapy form on the eCRF.
- Survival status – Please document the survival status of the patient (alive, alive with disease progression or deceased) and update the Survival Data eCRF. If applicable the disease progression form should be updated if the patient did not have disease progression previously during the trial.
- Pregnancy Testing (serum or urine) for WOCBP only. Please document the results and record this on the Pregnancy Test (logform) on the eCRF. A pregnancy notification form should be submitted if required.

5.3.5 *4 Months Post-Treatment Discontinuation Follow Up (WOCBP ONLY)*

This visit is **only applicable to WOCBP** and should occur 120 (±7) days post-treatment discontinuation

The following data should be obtained:

- CT Scans – Where a patient has undergone a routine CT scan, record this in the Ad Hoc CT scan folder on the eCRF.
- Additional therapy – Where a patient has received new cancer treatment outside of the trial, record this on the Post Study Therapy form on the eCRF.
- Pregnancy Testing (serum or urine). Please document the results and record this on the Pregnancy Test (logform) on the eCRF. A pregnancy notification form should be submitted if required.

5.3.6 *5 Months Post-Treatment Discontinuation Follow Up (WOCBP ONLY)*

This visit is **only applicable to WOCBP** and should occur 150 (±7) days post-treatment discontinuation.

The following data should be obtained:

- CT Scans – Where a patient has undergone a routine CT scan, record this in the Ad Hoc CT scan folder on the eCRF.
- Additional therapy – Where a patient has received new cancer treatment outside of the trial, record this on the Post Study Therapy form on the eCRF.
- Pregnancy Testing (serum or urine). Please document the results and record this on the Pregnancy Test (logform) on the eCRF. A pregnancy notification form should be submitted if required.

5.3.7 *6 Months Post-Treatment Discontinuation Follow Up*

This visit is applicable for all trial participants and should occur 180 (±14) days post treatment discontinuation.

The following data should be obtained:

- CT Scans – Where a patient has undergone a routine CT scan, record this in the Ad Hoc CT scan folder on the eCRF.
- Additional therapy – Where a patient has received new cancer treatment outside of the trial, record this on the Post Study Therapy form on the eCRF.
- EQ-5D-5L questionnaire
- Survival status – Please document the survival status of the patient (alive, alive with disease progression or deceased) and update the Survival Data eCRF. If applicable the disease progression form should be updated if the patient did not have disease progression previously during the trial.

5.3.8 *9 Months Post-Treatment Discontinuation Follow Up*

This visit is applicable for all trial participants and should occur 270 (±14) days post treatment discontinuation.

The following data should be obtained:

- CT Scans – Where a patient has undergone a routine CT scan, record this in the Ad Hoc CT scan folder on the eCRF.
- Additional therapy – Where a patient has received new cancer treatment outside of the trial, record this on the Post Study Therapy form on the eCRF.

- Survival status – Please document the survival status of the patient (alive, alive with disease progression or deceased) and update the Survival Data eCRF. If applicable the disease progression form should be updated if the patient did not have disease progression previously during the trial.

5.3.9 *12 Months Post-Treatment Discontinuation Follow Up*

This visit is applicable for all trial participants and should occur 360 (±14) days post treatment discontinuation.

The following data should be obtained:

- CT Scans – Where a patient has undergone a routine CT scan, record this in the Ad Hoc CT scan folder on the eCRF.
- Additional therapy – Where a patient has received new cancer treatment outside of the trial, record this on the Post Study Therapy form on the eCRF.
- EQ-5D-5L questionnaire
- Survival status – Please document the survival status of the patient (alive, alive with disease progression or deceased) and update the Survival Data eCRF. If applicable the disease progression form should be updated if the patient did not have disease progression previously during the trial.

5.3.10 *Follow Up after 12 Months Post-Treatment Discontinuation*

Patients should be followed up every 3 months to obtain the following data:

- Survival status – Please document the survival status of the patient (alive, alive with disease progression or deceased) and update the Survival Data eCRF. If applicable the disease progression form should be updated if the patient did not have disease progression previously during the trial
- Additional therapy – Where a patient has received new cancer treatment outside of the trial, record this on the Post Study Therapy form on the eCRF.

The most recent patient status and date last seen may also be requested before the end of trial is declared to ensure that data for PFS and OS analyses are as complete as possible.

5.4. DEVIATIONS AND SERIOUS BREACHES

Any study protocol deviations/violations and breaches of Good Clinical Practice occurring at sites should be reported to the SCTU and the local R&D Office immediately. SCTU will then advise of and/or undertake any corrective and preventative actions as required.

All serious protocol deviations/violations and serious breaches of Good Clinical Practice and/or the study protocol will immediately be reported to Sponsor, the regulatory authorities and other organisations, as required in the Medicines for Human Use (Clinical Trials) Regulations 2004, as amended.

5.5. TRIAL DISCONTINUATION

5.5.1 *Reasons for trial withdrawal*

A participant may withdraw, or be withdrawn, from trial treatment for the following reasons:

- Intolerance to treatment: Any clinical adverse event (AE), laboratory abnormality or intercurrent illness, which, in the opinion of the investigator, indicates that continued participation in the study is not in the best interest of the patient.
- Clinician's decision
- Termination of the study by Sponsor

- Loss of ability to freely provide consent through imprisonment or involuntarily incarceration for treatment of either a psychiatric or physical (e.g., infectious disease) illness
- Non-compliance with protocol treatment
- Pregnancy*

*In the case of pregnancy, the investigator must immediately notify the Sponsor of this event. In most cases, the study drug will be permanently discontinued in an appropriate manner. If the investigator determines a possible favourable benefit/risk ratio that warrants continuation of study drug, a discussion between the investigator, Sponsor and BMS must occur, if local regulations allow.

Patients may withdraw or be withdrawn from one of four levels:

- Patient withdrawal (**Level 1**) from trial treatment – participants stop trial treatment but remain in follow-up and continue to provide translational samples.
- Patient withdrawal (**Level 2**) from the translational study – participants continue trial treatment and follow-up but do not provide translational samples
- Patient withdrawal (**Level 3**) from the translational study and trial treatment - participants stop trial treatment and do not continue providing translational samples but remain in follow-up.
- Patients completely withdraw (**Level 4**) from the trial – participants stop the trial treatment, any follow-up activity and withdraw or no longer provide any translational sample collection.

If a participant wishes to withdraw from trial treatment, participating sites should explain the importance of remaining on trial follow up for the purposes of data capture only. Where possible, patients who have withdrawn from trial treatment should remain in follow-up as per the trial schedule. If patients additionally withdraw consent for this, they should revert to standard clinical care as deemed by the responsible clinician. It would remain useful for the trial team to continue to collect standard follow-up data and unless the patient explicitly states otherwise, follow-up data will continue to be collected. Patients who withdraw from treatment will continue to have assessments until disease progression. A withdrawal eCRF should be completed for each patient who withdraws from the study, recording the date and reason for withdrawal, if known.

Data and samples collected prior to participant withdrawal at any of the levels indicated above will be collected and used for trial analysis by SCTU. Participants who initially consented to be registered for patient flagging will remain on the system so that important research information on date and cause of death can be requested from NHS Digital.

In consenting to the study, participants have consented to the study intervention, follow-up and data collection. Participants may be discontinued from the study procedures at any time.

5.6. TREATMENT OF INFUSION RELATED REACTIONS

Since nivolumab contains only human immunoglobulin protein sequences, it is unlikely to be immunogenic and induce infusion or hypersensitivity reactions. Version 18 of the nivolumab IB states the following frequencies for monotherapy in the Reference Safety Information; IRR 0.21%, hypersensitivity 0.047%, anaphylactic reaction 0.024%. However, if such a reaction were to occur, it might manifest with fever, chills, rigors, headache, rash, pruritus, arthralgias, hypo- or hypertension, bronchospasm, or other symptoms. All Grade 3 or 4 infusion reactions should be reported as an SAE if criteria are met. Infusion reactions should be graded according to National Cancer Institute CTCAE (version 4.03) guidelines (see appendix 3). Treatment recommendations are provided below and may be modified based on local treatment standards and guidelines as appropriate:

For Grade 1 symptoms: (Mild reaction; infusion interruption not indicated; intervention not indicated)

Remain at bedside and monitor patient until recovery from symptoms. The following prophylactic premedications are recommended for future infusions: piriton 10 mg (or equivalent) and/or paracetamol 500 to 1000 mg at least 30 minutes before additional administrations.

All future infusions in these patients who have reacted, can be administered as per clinical choice which may be to increase the infusion time by doubling the rate to one hour with careful monitoring, during the infusion, for any reaction.

For Grade 2 symptoms: (Moderate reaction; requires therapy or infusion interruption but responds promptly to symptomatic treatment [e.g. antihistamines, non-steroidal anti-inflammatory drugs, opioids, corticosteroids, bronchodilators, IV fluids]; prophylactic medications indicated for 24 hours).

Stop the infusion, begin an IV infusion of normal saline, and treat the patient with piriton 10 mg IV (or equivalent) and/or paracetamol 500 to 1000 mg; remain at bedside and monitor patient until resolution of symptoms.

Corticosteroid or bronchodilator therapy may also be administered as appropriate. If the infusion is interrupted, then restart the infusion at 50% of the original infusion rate when symptoms resolve; if no further complications ensue after 30 minutes, the rate may be increased to 100% of the original infusion rate. Monitor patient closely. If symptoms recur then no further IMP will be administered at that visit. Administer piriton 10 mg IV, and remain at bedside and monitor the patient until resolution of symptoms. The amount of study drug infused must be recorded on the electronic case report form (eCRF).

The following prophylactic premedications are recommended for future infusions: piriton 10 mg (or equivalent) and/or paracetamol 500 to 1000 mg should be administered at least 30 minutes before additional nivolumab administrations. If necessary, corticosteroids (recommended dose: up to 25 mg of IV hydrocortisone or equivalent) may be used.

All future infusions in these patients who have reacted, can be administered as per clinical choice which may be to increase the infusion time by doubling the rate to one hour with careful monitoring, during the infusion, for any reaction.

For Grade 3 or Grade 4 symptoms: (Severe reaction, Grade 3: prolonged [i.e., not rapidly responsive to symptomatic medication and/or brief interruption of infusion]; recurrence of symptoms following initial improvement; hospitalisation indicated for other clinical sequelae [e.g., renal impairment, pulmonary infiltrates]. Grade 4: (life-threatening; pressor or ventilator support indicated).

Immediately discontinue infusion. Begin an IV infusion of normal saline, and treat the patient as follows. Recommend bronchodilators, adrenaline 0.2 to 1 mg of a 1:1,000 solution for subcutaneous administration or 0.1 to 0.25 mg of a 1:10,000 solution injected slowly for IV administration, and/or piriton10 mg IV with methylprednisolone 100 mg IV (or equivalent), as needed. Patient should be monitored until the investigator is comfortable that the symptoms will not recur. **IMP will be permanently discontinued.** Investigators should follow their institutional guidelines for the treatment of anaphylaxis. Remain at bedside and monitor patient until recovery from symptoms.

In the case of late-occurring hypersensitivity symptoms (e.g., appearance of a localised or generalised pruritus within 1 week after treatment), symptomatic treatment may be given (e.g., oral antihistamine, or corticosteroids).

5.7. PROHIBITED AND RESTRICTED THERAPIES DURING THE TRIAL

The following medications are prohibited during the study (unless utilised to treat a drug related adverse event):

- Immunosuppressive agents
- Immunosuppressive doses of systemic corticosteroids (except as stated in Section 5.7.2)

- Any concurrent anti-neoplastic therapy (i.e., chemotherapy, hormonal therapy, immunotherapy, extensive, non-palliative radiation therapy, or standard or investigational agents for treatment of mesothelioma)
- Live vaccination

Caution must be used with ototoxic or nephrotoxic concomitant drugs.
Caution should be used regarding the use of herbal medications as there may be unknown interactions with nivolumab. Discontinuation of the use of herbal medications prior to study enrolment is encouraged.

5.7.1 *Other Restrictions and Precautions*

It is the local imaging facility's responsibility to determine, based on patient attributes (e.g. allergy history, diabetic history and renal status), the appropriate imaging modality and contrast regimen for each patient. Imaging contraindications and contrast risks should be considered in this assessment. Patients with renal insufficiency should be assessed to determine whether they should receive contrast and, if so, what type and dose is appropriate.

5.7.2 *Permitted Therapy*

Corticosteroids

A brief (**less than 3 consecutive weeks**) course of non-immunosuppressive doses (see below) corticosteroids for prophylaxis (e.g., contrast dye allergy) or for treatment of non-autoimmune conditions (e.g. delayed-type hypersensitivity reaction caused by a contact allergen) is permitted.

Patients are therefore permitted the use of oral, topical, ocular, intra-articular, intranasal, and inhalational corticosteroids (with minimal systemic absorption) at doses no greater than 10mg prednisone (*equivalent to 1.5 mg dexamethasone, 40 mg Hydrocortisone, or 8 mg Methylprednisolone*). Adrenal replacement steroid doses > 10 mg daily prednisone are permitted.

Immunosuppressive doses of steroids are permitted to treat toxicities (as per recommended in the Safety Management Algorithms in Appendix 4), however nivolumab/placebo should not be given at the same time. Patients should be weaned to non-immunosuppressive doses prior to re-commencing nivolumab/placebo.

Other Medications

Regular concomitant use of bisphosphonates and RANK-L inhibitors for prevention or reduction of skeletal-related events in patients with bone metastases is allowed if initiated prior to first dose of study therapy.

5.8. BLINDING AND PROCEDURES FOR UNBLINDING

Patient allocation will be provided to unblinded pharmacy staff via an IWRS. The randomisation code will be retained by the system.

Deaths and serious adverse events (SAE) will be reviewed in a blinded manner. If a cause of death or SAE is unexpected (i.e. not listed in the RSI approved section of the nivolumab IB) and considered drug related the trial statistician and Quality and Regulatory team (SCTU) will be able to unblind the patient's treatment assignment.

5.8.1 *Emergency Unblinding*

Sites must be able to provide a 24hr emergency unblinding service for their patients. In the case of adverse events in which patient care would vary dependent on treatment allocation, emergency unblinding may be performed by unblinded pharmacy staff or in cases where it is the site's local policy for on-call doctors to unblind, this will be permitted. Sites must have tested procedure for out-of-hours emergency unblinding. In these situations, discussion with the Chief Investigator is not required. If patient allocation is disclosed, sites should report to SCTU the date, time, reason for unblinding, name of person requesting the code break, and name of person breaking the code using the trial specific Emergency Unblinding Notification Form. Once unblinded, patients must discontinue CONFIRM trial treatment. Unblinding reports should be filed in the patient's medical records at site and in the trial master file (without disclosing the treatment allocation). The Trial Manager or Senior Trial Manager will ensure that the Statistician and Senior Statistician are fully informed of all cases of unblinding.

All further details are outlined in the study specific procedure for unblinding.

5.8.2 *Non-Emergency Unblinding*

Patients may need to be unblinded if they are being considered for further treatment following treatment within the CONFIRM trial (i.e. post progression or treatment discontinuation) and there are concerns about interaction with nivolumab. In these circumstances, unblinding should be discussed with the Chief Investigator first. The Request for Non-Emergency Unblinding Form should be completed and emailed to confirmtrial@soton.ac.uk who will then request that the Statistician or Senior Statistician unblinds and contacts the treating clinician with the treatment allocation. Unblinding reports should be filed in the patient's medical records at site and in the trial master file (without disclosing the treatment allocation). Once patients are unblinded, they must discontinue CONFIRM trial treatment.

If a patient is accidently unblinded their future participation in the trial should be discussed with SCTU and the CI on a case-by-case basis.

All further details are outlined in the study specific procedure for unblinding.

6 TREATMENTS

6.1. TREATMENT SCHEDULE

Patients with relapsed mesothelioma will be randomised to receive either nivolumab therapy or placebo. Patients will be treated until progression or for a maximum of one year.

6.2. IMP SUPPLY

6.2.1 *Nivolumab*

Nivolumab is considered an investigational medicinal product for the purpose of this protocol. Nivolumab will be provided free of charge by BMS for patients recruited to the trial and will be study specific investigational stock.

BMS will pack, label, Qualified Person release and distribute the IMP for this study. Nivolumab will be supplied in single use vials and prepared at site according to CONFIRM Pharmacy Manual. Nivolumab must be kept secure at site and stored between 2°C to 8°C, protected from light and not frozen or shaken.

Re-ordering will be via the Bracket system. Only pharmacy staff will be unblinded to patient treatment allocation.

Preparation

The administration of nivolumab infusion must be completed within 24 hours of preparation. If not used immediately, the infusion solution may be stored under refrigerated conditions (2°C to 8°C) for up to 24 hours and a maximum of the total 24 hours can be at room temperature (25°C) and room light. The maximum 8 hours under room temperature and room light conditions includes the product administration period. Further details on preparation should be taken from the Investigator's Brochure approved for use in this trial.

Temperature excursions
All temperature excursions outside the storage conditions specified for nivolumab in the IB/Pharmacy Manual/labels must be reported to SCTU as outlined in the Pharmacy Manual.
Upon identifying an excursion:

- All affected trial stock must be quarantined IMMEDIATELY
- The CONFIRM Temperature Excursion Form must be completed and e-mailed to confirmtrial@soton.ac.uk

Please note that SCTU must be informed immediately if a patient has been administered drug affected by a temperature excursion.

6.2.2 *Placebo*

Patients who are randomised to receive placebo will receive sterile 0.9% sodium chloride. This will be supplied by participating sites.

6.3. ADMINISTRATION

Patient will receive treatment with nivolumab at a dose of 240mg (or placebo) as a 30-minute IV infusion, on Day 1 (±2) of every 14 day treatment cycle, until progression, unacceptable toxicity, withdrawal of consent, or the maximum treatment duration of 12 months is reached, whichever occurs first. There will be no dose escalations or reductions of IMP allowed. Patients may be dosed no less than 12 days from the previous dose.

The nivolumab or placebo must be infused intravenously over a period of 30 minutes. The infusion must be administered through a sterile, non-pyrogenic, low protein binding in-line filter with a pore size of 0.2-1.2 µm. It should not be administered as an intravenous push or bolus injection. The total dose can be administrated either:

- Without dilution, after transfer to an infusion container using an appropriate sterile syringe; or
- After diluting according to the following instructions:
    - The final infusion concentration should range between 1 and 10 mg/mL.
    - The total volume of infusion must not exceed 160 mL. For patients weighing less than 40 kg, the total volume of infusion must not exceed 4 mL per kilogram of patient weight.

Nivolumab concentrate may be diluted with either sodium chloride 9 mg/mL (0.9%) solution for in or 50 mg/mL (5%) glucose solution for injection.

After administration flush the line with sodium chloride 9mg/ml (0.9%) solution for injection or 50mg/ml (5%) glucose solution for injection.

Any unused medicinal product waste material should be disposed of as per local practice.

Patients should be carefully monitored for infusion reactions during nivolumab or placebo administration. If an acute infusion reaction is noted, patients should be managed according to Section 5.6. Doses of IMP may be interrupted, delayed, or discontinued depending on how well the patient tolerates the treatment. See Section 6.6 for more details regarding dose delays, retreatment, and discontinuations.

6.4. ACCOUNTABILITY

Accountability and dispensing logs will be required for nivolumab/placebo supplied in the trial. These logs will be supplied to sites by SCTU.

6.5. CONCOMITANT MEDICATIONS

Information on any treatment received by the participant, along with dose, frequency and therapeutic indication, from 14 days prior to starting trial treatment up to 30 days after the last dose of nivolumab or placebo will be recorded in the electronic case report form (eCRF).

6.6. DOSE DELAYS AND MODIFICATIONS FOR TOXICITY

Tumour assessments for all patients should continue as per protocol **even if dosing is delayed**.

Dose Delay Criteria

IMP administration should be delayed for the following:

- Any Grade 2 non-skin, drug-related adverse event, except for fatigue and laboratory abnormalities
- Any Grade 3 skin drug-related AE
- Any AE, laboratory abnormality or inter-current illness, which in the judgment of the investigator, warrants delaying the dose of study medication.
- Any Grade 3 drug-related laboratory abnormality with the following exceptions for lymphopenia, AST, ALT, or total bilirubin or asymptomatic amylase or lipase:
    - Grade 3 lymphopenia does not require a dose delay
    - If a patient has a baseline AST, ALT, or total bilirubin that is within normal limits, delay dosing for drug-related Grade 2 toxicity
    - If a patient has baseline AST, ALT, or total bilirubin within the Grade 1 toxicity range, delay dosing for drug-related Grade 3 toxicity
    - Any Grade 3 drug-related amylase or lipase abnormality that is not associated with symptoms or clinical manifestations of pancreatitis does not require dose delay. The chief investigator should be consulted for such Grade 3 amylase or lipase abnormalities.

Patients who require delay of IMP should be re-evaluated weekly or more frequently if clinically indicated and resume dosing when re-treatment criteria are met (per Appendix 4).

Treatment can be delayed for up to four weeks from the last dose. Delays for longer than four weeks should be discussed with the Chief Investigator.

For details of specific adverse event management, refer to safety management algorithms in appendix 4.

7 SAFETY

7.1. DEFINITIONS

The Medicines for Human Use (Clinical Trials) Regulations 2004, as amended, provides the following definitions relating to adverse events in trials with an investigational medicinal product:

Adverse Event (AE): any untoward medical occurrence in a participant or clinical trial participant administered a medicinal product and which does not necessarily have a causal relationship with this treatment.

An AE can therefore be any unfavourable and unintended sign (including an abnormal laboratory finding), symptom, or disease temporally associated with the use of an investigational medicinal product (IMP), whether or not considered related to the IMP.

Adverse Reaction (AR): all untoward and unintended responses to an IMP related to any dose administered.
All AEs judged by either the reporting investigator or the sponsor as having reasonable causal relationship to a medicinal product qualify as adverse reactions. The expression reasonable causal relationship means to convey in general that there is evidence or argument to suggest a causal relationship.

Unexpected Adverse Reaction (UAR): an AR, the nature or severity of which is not consistent with the applicable product information (e.g. investigator's brochure (IB) for an unapproved investigational product or summary of product characteristics (SmPC) for an authorised product).
When the outcome of the adverse reaction is not consistent with the applicable product information this adverse reaction should be considered as unexpected. Side effects documented in the RSI which occur in a more severe form than anticipated are also considered to be unexpected.

Serious Adverse Event (SAE) or **Serious Adverse Reaction or Suspected Unexpected Serious Adverse Reaction (SUSAR):** any untoward medical occurrence or effect that at any dose:

- **Results in death**
- **Is life-threatening** – *refers to an event in which the participant was at risk of death at the time of the event; it does not refer to an event which hypothetically might have caused death if it were more severe*
- **Requires hospitalisation*, or prolongation of existing hospitalisation**
- **Results in persistent or significant disability or incapacity**
- **Is a congenital anomaly or birth defect**
- **Important medical events****.

*Hospitalisation is defined as an inpatient admission, regardless of length of stay, even if the hospitalisation is a precautionary measure for continued observation. Hospitalisations for a pre-existing condition, including elective procedures that have not worsened, do not constitute an SAE.

**Other important medical events are defined as a medical event(s) that may not be immediately life-threatening or result in death or hospitalisation but, based upon appropriate medical and scientific judgment, may jeopardise the subject or may require intervention (e.g., medical, surgical) to prevent one of the other serious outcomes listed in the definition above. Examples of such events include, but are not limited to, intensive treatment in an emergency room or at home for allergic bronchospasm; blood dyscrasias or convulsions that do not result in hospitalisation.

Suspected transmission of an infectious agent (e.g. pathogenic or non-pathogenic) via the study drug is an SAE.

Although pregnancy, overdose, cancer, and potential drug induced liver injury (DILI) are not always serious by regulatory definition, these events must be handled as SAEs, and reported in the same time frames.

Any component of a study endpoint that is considered related to study therapy should be reported as an SAE (e.g. death is an endpoint, if death occurred due to anaphylaxis, anaphylaxis must be reported and disease progression that is considered related to study treatment).

Note: *It is the responsibility of the PI or delegate to grade an event as 'not serious' (AE) or 'serious' (SAE).*

Suspected Unexpected Serious Adverse Reaction (SUSAR): any suspected adverse reaction related to an IMP that is both unexpected and serious.

Death
As outlined below, if a patient's death is considered related to study therapy it should be reported as an SAE.

As per section 7.3, deaths due to disease progression of mesothelioma are exempt from SAE reporting, unless progression is considered related to the IMP/placebo.

Please note if the cause of death is unknown or the cause of death is pending, this must be submitted as an SAE.

Deaths should be entered on Rave within 24 hours of becoming aware.

Laboratory Test Abnormalities
All laboratory test results captured as part of the study should be recorded following local procedures. Test results that constitute SAEs should be documented and reported as such.

The following laboratory abnormalities should be documented and reported appropriately:

- any laboratory test result that is clinically significant or meets the definition of an SAE
- any laboratory abnormality that required the subject to have study drug discontinued or interrupted
- any laboratory abnormality that required the subject to receive specific corrective therapy.

It is expected that wherever possible, the clinical rather than laboratory term would be used by the reporting investigator (e.g., anaemia versus low haemoglobin value).

Potential Drug Induced Liver Injury (DILI)
Potential drug induced liver injury is defined as:

1) AT (ALT or AST) elevation > 3 times upper limit of normal (ULN)
   AND
2) Total bilirubin > 2 times ULN, without initial findings of cholestasis (elevated serum alkaline phosphatase),
   AND
3) No other immediately apparent possible causes of AT elevation and hyperbilirubinemia, including, but not limited to, viral hepatitis, pre-existing chronic or acute liver disease, or the administration of other drug(s) known to be hepatotoxic.

Wherever possible, timely confirmation of initial liver-related laboratory abnormalities should occur prior to the reporting of a potential DILI event. All occurrences of potential DILIs, meeting the defined criteria, must be reported as SAEs.

Overdose
An overdose is defined as the accidental or intentional administration of any dose of a product that is considered both excessive and medically important. All occurrences of overdose must be reported as an SAE.

7.2. REPORTING WINDOWS
All AEs must be reported from the date of consent until 100 days post treatment discontinuation and for ongoing drug-related AEs until resolved, return to baseline or deemed irreversible.

See below for SAE reporting windows:

- **Between informed consent and first dose of nivolumab/placebo:** All events that are considered SAEs, that are deemed by the investigator to be related to trial procedures.
- **From first dose of nivolumab/placebo until 100 days after last administration of nivolumab/placebo:** All events considered to be SAEs
- **>100 days after last administration of nivolumab/placebo:** All events that are considered to be SAEs, that are deemed by the investigator to be related to nivolumab/placebo and/or trial procedures.

7.3. SERIOUSNESS

A complete assessment of the seriousness must always be assessed by a medically qualified doctor who is registered on the delegation of responsibility log; this is usually the investigator.

All adverse events that fulfil the definition of 'serious' in protocol section 7.1, must be reported to SCTU using the Serious Adverse Event Report Form. Specific exceptions to this (as listed below) should be recorded as AEs rather than SAEs.

All SAEs must be reported immediately by the PI or delegate at the participating centre to the SCTU.

Exceptions:

For the purposes of this trial, the following SAEs **do not** require reporting to SCTU using the Serious Adverse Event Report Form:

- Death due to disease progression of mesothelioma, unless progression is considered related to the IMP/placebo
- Hospitalisations for elective treatment of a pre-existing condition
- SAEs occurring prior to cycle 1, day 1 that are not considered to be related to trial procedures

7.4. CAUSALITY

A complete assessment of the causality must always be assessed by a medically qualified doctor who is registered on the delegation of responsibility log; this is usually the investigator.

If there is any doubt about the causality the local investigator should inform SCTU who will notify the Chief Investigator. BMS and/or other clinicians may be asked for advice in these cases.

In the case of discrepant views on causality between the Investigator and others, SCTU will classify the event as per the worst-case classification and if onward reporting is required, the MHRA will be informed of both parties' points of view.

Relationship	Description	Denoted
**Unrelated**	There is no evidence of any causal relationship between study drug administration and the AE	SAE
**Related**	There is a reasonable causal relationship between study drug administration and the AE	SAR/SUSAR

7.5. EXPECTEDNESS

Expectedness assessments are made against the approved reference safety information (RSI). The RSI for this trial is specified within the document versions listed in the table below:

Name of Product	IB/SmPC	Manufacturer	Version date	Version	Section	Table
Nivolumab	IB	Bristol-Myers Squibb	25 June 2019	18	Appendix 1	1-1

The nature or severity of the event should be considered when making the assessment of expectedness.

If these factors are not consistent with the current information available then the SAE should be recorded as 'unexpected'.

7.6. REPORTING PROCEDURES

All adverse events should be reported during the reporting windows outlined in section 7.2. Depending on the nature of the event, the reporting procedures below should be followed. Any questions concerning adverse event reporting should be directed to the SCTU in the first instance. A flowchart will be provided to aid in the reporting procedures.

7.6.1 *Reporting Details*

For all SAEs, SARs and SUSARs an SAE report form should be completed with as much detail as possible (including any relevant anonymised treatment forms and/or investigation reports) and faxed/emailed to SCTU immediately but at least within 24 hours of site becoming aware of the event.

Or

Contact SCTU by phone for advice and then fax or email a scanned copy of the SAE report form completed as above.

SAE REPORTING CONTACT DETAILS

Please email or fax a copy of the SAE form to

SCTU within 24 hours of becoming aware of the event

Fax: 0844 774 0621 or Email: ctu@soton.ac.uk

FAO: Quality and Regulatory Team

For further assistance: Tel: 023 8120 4138 (Mon to Fri 09:00 – 17:00)

The SAE report form asks for nature of event, date of onset, severity, outcome, causality and expectedness. The responsible investigator (or delegate) should assign the seriousness, causality and expectedness of the event with reference to the approved IMP IB/SmPC and provide version used for the assessment.

The event term should be the most appropriate medical term or concept (which the SCTU will code to MedDRA) and grades given in accordance with the NCI CTCAE v4.03

.

Additional information should be provided as soon as possible if all information was not included at the time of reporting, but no more than 7 days after initial report.

7.6.2 ***In addition to the definition above, any suspected transmission via a medicinal product of an infectious agent is also considered an SAE and may be subject to expedited reporting requirements in some countries. Any organism, virus or infectious particle (for example Prion Protein Transmitting Transmissible Spongiform Encephalopathy), pathogenic or non-pathogenic, is considered an infectious agent. Elevations in liver biochemistry that meet Hy's Law criteria are reported as SAEs, using the important medical event serious criterion if no other criteria are applicable. Follow up and post-trial SAEs***

The reporting requirement for SAEs affecting participants applies for all events occurring up to 100 days after the last administration of trial drugs.

All unresolved adverse events should be followed by the investigator until resolved, the participant is lost to follow-up, or the adverse event is otherwise explained. At the last scheduled visit, the investigator should instruct each participant to report any subsequent event(s) that the participant, or the participant's general practitioner, believes might reasonably be related to participation in this trial. The investigator should notify the trial sponsor of any death or adverse event occurring at any time after a participant has discontinued or terminated trial participation that may reasonably be related to this trial.

7.6.3 *Non-serious AEs*

All adverse events should be recorded in the relevant eCRF.

7.6.4 *Pre-existing Conditions*

Medically significant pre-existing conditions (prior to treatment (cycle 1 day 1)) should not be reported as an AE unless the conditions worsen during the trial. The condition, however, must be reported on the Medical History eCRF. Any adverse events that occur after the first dose of nivolumab/placebo should be recorded on the AE eCRF.

All events that are considered SAEs, as defined in section 7.1, that are deemed by the investigator to be related to trial procedures should be reported from date of consent.

7.6.5 *Serious Adverse Events and Reactions*

All SAEs, SARs and SUSARs should be reported within 24 hours of the local site becoming aware of the event. The SAE/SUSAR form asks for nature of event, date of onset, severity, corrective therapies given, outcome, causality (i.e. related or unrelated) and expectedness. The responsible investigator should assign the causality and expectedness of the event with reference to the approved RSI. The event term should be graded in accordance with the NCI CTCAE v4.03. Additional information should be provided as soon as possible if the event/reaction has not resolved at the time of reporting.

7.6.6 *Pregnancy*

If a participant or their partner becomes pregnant whilst taking part in the trial or during a stage where the foetus could have been exposed to an IMP (up to 5 months following IMP administration for female patients and up to 7 months for female partners of male participants), the investigator must ensure that the participant and/or participant's partner and the participant's and/or participant's partner's healthcare professional are aware that follow up information is required on the outcome of the pregnancy (provided consent is given).

The investigator must immediately notify SCTU of this event via the Pregnancy Surveillance Form within 24 hours of becoming aware.

Protocol-required procedures for study discontinuation and follow-up must be completed.

Follow-up is, of course, dependent on obtaining informed consent for this from the participant (or their partner in the case of male trial subjects). The site must request consent from the pregnancy participant or female partner of a male participant to report information regarding a pregnancy using:

- For female participants: The trial specific Pregnant Patient Information Sheet and Pregnant Patient Informed Consent Form
- For female partners of male participants: The trial specific Pregnant Partner Information Sheet and Pregnant Partner Informed Consent Form

Follow-up information regarding the course of the pregnancy, including perinatal and neonatal outcome and, where applicable, offspring information must be reported on the Pregnancy Surveillance Form.

If consent is not given, the notification that a pregnancy has occurred will be retained by SCTU, however follow up information will not be collected.

If the participant leaves the area, their new healthcare professional should also be informed.

7.7. SCTU RESPONSIBILITIES FOR SAFETY REPORTING TO REC

SCTU will notify the necessary REC of all UK SUSARs occurring during the trial according to the following timelines; fatal and life-threatening within 7 days of notification and non-life threatening within 15 days.

SCTU submit all safety information to the REC in annual progress reports.

7.8. SCTU RESPONSIBILITIES FOR SAFETY REPORTING TO MHRA

SCTU will notify the necessary competent authorities of all SUSARs occurring during the trial according to the following timelines: fatal and life threatening within 7 days of notification and non-life threatening within 15 days.

SCTU submit the Developmental Safety Update Reports (DSUR) to MHRA annually.

7.9. SCTU RESPONSIBILITIES FOR SAFETY REPORTING TO BMS

SCTU will notify BMS of all SAEs/SARs/SUSARs occurring during the trial within 24 hours of sponsor's awareness. Any updates will be sent to BMS as received.

SCTU will submit a copy of the DSUR to BMS annually.

SCTU will reconcile transmitted SAEs between the BMS safety database and the clinical database. Frequency of reconciliation will be determined prior to study commencement. BMS GPV&E will e-mail upon request from the Investigator, the BMS GPV&E safety database reconciliation report in Excel format. Requests for reconciliation should be sent to **aepbusinessprocess@bms.com**.

Reconciliation is at the case level. If SCTU identifies any cases which were not transmitted to GPV&E, they will immediately transmit the case to worldwide.safety@bms.com. Data elements on the BMS GPV&E safety database reconciliation report such as patient ID, SAE event term, SAE onset date, death and date of death are used to facilitate reconciliation.

The GPV&E safety database reconciliation report will function as a cumulative spreadsheet and will be used to track cases transmitted to BMS GPV&E.

8 STATISTICS AND DATA ANALYSES

8.1. METHOD OF RANDOMISATION

Patients will be randomised to either nivolumab or the control arm on a 2:1 allocation. Patients will be stratified according to epithelioid versus non-epithelioid.

8.2. SAMPLE SIZE

The original sample size calculation was based on overall survival (OS) only (see full details below). This calculation accounted for a number of interim analyses (efficacy and futility) by using an alpha of 0.04 for the sample size (and ultimately the interpretation). During the course of the trial, the independent Trial Steering Committee (TSC) approved the inclusion of progression-free survival (PFS) as a co-primary endpoint in order to mitigate against the risk of treatment cross-over, on the condition of maintaining the original sample size for OS. The analysis will follow the Fallback procedure [25], though no explicit correction or acknowledgement of the impact of the analysis (where alpha is split across the endpoints) was initially proposed.

Following a later review by the TSC (meeting date 13/01/2020), all future interim analyses (which were formal rule-based efficacy and futility analyses) were removed; this decision was taken for three

reasons: 1) a subset of the interim analyses were based on the PD-L1 positive subgroup, but analyses based on this could not take place until after recruitment was complete lessening the potential benefit of carrying out these analyses; 2) the percentage of the samples that were PD-L1 positive was lower than anticipated at the start of the trial (due to changes in how the analysis is carried out using an approved 22C3 antibody assay - meaning a greater number of patients would need to be recruited to obtain a sufficient number who were PD-L1 +ve to conduct the analysis); and 3) the futility analyses would also not take place until very near the end (or after) the recruitment phase had completed. Due to 1) and 2) above it was not possible to undertake the PD-L1 +ve analysis during the recruitment period of the trials and as there was also little benefit in being able to stop the study for futility after the recruitment period, the TSC were keen to allow the study to run to completion. (Note: one analysis for harm had been carried out prior to this meeting, which was not relevant for the original sample size calculation.)

It was agreed that removing the formal interim analyses and accounting for the Fallback procedure in the final analysis would have no net effect on the sample size calculation, meaning the original sample size calculations hold. Although the original sample size calculation accounted for interim analyses that have been subsequently removed, the Fallback procedure should be accounted for. The result is that an alpha of 0.04 is to be used for the analysis of OS (as originally proposed), and, according to the Fallback procedure, an alpha for PFS of 0.01 (if OS null hypothesis is rejected) or 0.05 (if OS null hypothesis is not rejected). This approach maintains an overall 5% type 1 error rate across the co-primary endpoints, and will serve the purpose of demonstrating a treatment effect on either OS or PFS that is sufficient to establish clinical benefit; i.e., if the analysis of OS shows a statistically significant effect (at 0.04) this will be sufficient to establish clinical benefit (irrespective of whether PFS is statistically significant or not), but if OS is non-significant, clinical benefit may still be established if PFS is statistically significant (at remaining unused alpha level of 0.01).

Power calculation for OS

Based on the VANTAGE trial [20] the median survival of patients on placebo is approximately 6 months.

Sample size assumptions (using artsurv in Stata): Based on a hazard ratio of 0.70 (equivalent to extending the median overall survival rate from 6 months to 8.5 months, or increasing the 6 months overall survival rate from 50% to 61.5% - considered a clinically significant difference by the research team); 80% power; recruitment period of 4 years, then 6 months follow-up period; 2-sided significance level of 4% (based on Fallback procedure); negligible drop out. Number of patients required: 336 (224 in the experimental arm and 112 in the control arm), a total of 291 events (deaths).

Power calculation for PFS

Based on the DETERMINE trial [24] the median PFS of patients on placebo is approximately 3 months.

The original sample size of 336 patients will also provide 80% power to detect a hazard ratio of 0.65 (equivalent to extending the median PFS from 3 months to 4.6 months or increasing the 3 month PFS rate from 50% to 63.7% - considered a clinically significant difference by the research team); with recruitment period of 4 years; 6 months follow-up period; 2-sided significance level of 1% (remaining unused alpha from Fallback procedure for 5% level overall); negligible drop out. This will require a total of 284 events (progression or deaths) (calculated using artsurv in Stata).

Power calculation for PD-L1 positive group

Previous versions of the protocol reported 80% power to detect a hazard ratio of 0.5 for OS (increasing survival at 6 months from 50 to 71%) based on alpha of 0.01 and 132 participants (40%) being PD-L1 positive. With the updated expectation of 25-30% (n=84 to 100) being PD-L1 positive, the study will have approximately 80-85% power to detect a hazard ratio of 0.5 with alpha of 0.04 (where the fallback method will be used to analyse and hence is split across OS and PFS). No corrections are made to the

sample size to account for running this subgroup analysis, as this analysis is deemed to be asking a different research question to the main study, i.e., the effectiveness of treatment in a different population.

8.3. STATISTICAL ANALYSIS PLAN (SAP)

A detailed statistical analysis plan will be developed prior to database lock, and all data and appropriate documentation will be stored for a minimum of 25 years after the completion of the trial.

Study populations:

- Intention-to-treat (ITT) population – consists of all patients who have consented and been randomised to a treatment arm.
- Safety population – consists of the ITT population who have received at least one dose of treatment.

The primary analysis population is the ITT population.

Time to event data (OS, PFS) will be analysed and presented using Kaplan-Meier curves for the ITT population. We will use a Cox proportional hazards model to calculate the HR, 95% CIs and p-value, adjusted for stratification factor (epithelioid vs. non-epithelioid), plus line of therapy and centre. The adjusted Cox regression models for OS and PFS will form the primary endpoint analyses models. Analysis will follow the Fallback procedure [25], where the alpha used to judge statistical significance for PFS will depend on the result for OS. OS will be judged at alpha of 0.04, and PFS will be 0.05 or 0.01 depending on the statistical significance or not, respectively, of OS. As noted in the sample size section, clinical benefit may be determined by results for either OS or PFS.

The primary analysis will be repeated for the PD-L1 positive subgroup(s) to assess the effectiveness in this group that are thought to be more likely to respond to treatment. This involves analysis of OS and PFS according to the Fallback procedure. Benefits for this group may be determined by results for either OS or PFS.

We will assess toxicity by comparing proportions of haematological and non-haematological toxicities during treatment with Pearson's χ^2 tests in the safety population. We will assess treatment compliance by comparing proportions of patients completing and not completing treatment with Pearson's χ^2 tests in in the ITT population.

All analyses will be carried out using STATA 13 or higher and/or SAS 9.4 or higher.

8.4. HEALTH ECONOMIC ANAYSIS PLAN

The economic analysis will include: i) a 'within-trial' cost-effectiveness analysis, to compare the costs and health outcomes (QALYs) accrued over the follow up period for patients in the intervention and control arms; and ii) development of a cost-effectiveness model to extrapolate cost and QALY estimates over a lifetime horizon. The analyses will follow the recommended methods and 'reference case' recommended by NICE, including: an NHS and Personal Social Services perspective for costing; estimation of QALYs using EQ-5D data and UK value sets, and discounting of costs and QALYs at 3.5% per year.

8.4.1 *Within trial economic analysis*

This will rely on individual-level data collected during the trial. Information on patients' use of health and social services that might be related to cancer care or treatment, including adverse effects, will be collected from routine records, and a patient questionnaire administered at follow up. Individual costs will then be calculated, using unit costs for resource items obtained from routine national sources,

including NHS Reference Cost or Tariff prices, the Drug Tariff, and the Personal Social Services Research Unit (PSSRU) Unit Costs of Health and Social Care. QALYs will be calculated from EQ-5D and mortality data, using the area under the curve method. Health-related quality of life (EQ-5D 'utility' scores) will be calculated at baseline and all follow-up points. For patients who die during the trial, utility is set to zero from the date of death. Regression analysis will be used to estimate mean cost and mean QALY differences between the study groups. Analysis will be pre-specified, and will take into consideration the need for multiple imputation for missing data, adjustment for baseline co-variates, inclusion of an interaction term for pre-specified subgroups (e.g. by biomarker), and the possibility of clustering by centre. Results will be presented as a ratio – the incremental cost per QALY gained with nivolumab compared with no treatment. Non-parametric bootstrapping will be used to obtain estimates of joint uncertainty over mean costs and QALYs, which will be represented by a scatterplot on the cost-effectiveness plane, and as a cost effectiveness acceptability curve (CEAC) – showing the probability that nivolumab is cost effective as a function of willingness to pay per QALY (the cost-effectiveness 'threshold').

8.4.2 *Economic modelling*

Modelling will be conducted according to International Society for Pharmacoeconomics and Outcomes Research (ISPOR) guidelines. Related NICE technology appraisals and published economic evaluations will be reviewed to inform consideration of model design and sources of evidence. The model will probably take the form of a 'Markov-type' health state transition model, although we will consider whether an individual-level simulation model will add value. The model specification will be discussed and agreed with members of the project team and other experts. Input parameters will be estimated from trial data (e.g. by fitting of survival functions), supplemented by evidence from the literature when necessary (e.g. to inform assumptions about longer-term survival). Systematic search methods will be used to identify best-available sources of evidence. Probabilistic sensitivity analysis will be used to estimate how uncertainty over input parameters results in uncertainty over the model results. As with the within-trial analysis, results will be presented as an incremental cost effectiveness ratio, and with a cost-effectiveness scatterplot and CEAC curve. A value of information analysis will also be conducted to estimate the potential value of conducting further research to reduce residual uncertainty. In addition, uncertainties relating to model structure or choice of data sources will be explored through deterministic sensitivity analysis. Subgroup analysis will be used to explore potential differences in cost-effectiveness by patient group.

9 TRANSLATIONAL ANALYSIS

Two types of mandatory biological samples will be collected from CONFIRM patients for translational research within the CONFIRM trial and future ethically approved research; peripheral blood and fresh or archival baseline tissue. The analysis of these samples is integral to the trial and therefore provision of these samples is mandatory and in the inclusion criteria for this trial and built into the main trial consent. Patients will also be asked to donate an optional tissue sample upon disease progression.

There is one central laboratory for this trial:
Mesothelioma Research Programme, University of Leicester

9.1. DIAGNOSTIC BIOPSY OR SURGICAL SAMPLES

Baseline fresh or archival formalin fixed paraffin embedded (FFPE) tissue will be sent to the University of Leicester for all CONFIRM patients. This will be from a tumour mass biopsy. Re-biopsy is required if an archival sample if not available. See the CONFIRM Laboratory Manual for details on how to send and log FFPE samples.

If patients consent, FFPE tissue from a biopsy upon disease progression will also be sent to the University of Leicester.

9.2. PERIPHERAL BLOOD SAMPLES

Peripheral blood samples will be collected all CONFIRM patients at the following timepoints (in accordance with the schedule in section 5):

- Baseline (within three working days prior to first treatment (Cycle 1, day 1)
- 28 ±7 days post treatment discontinuation

1 x 10 ml EDTA tube and 1 x 10 ml red top vacutainer serum tube should be collected and processed according to the CONFIRM Lab Manual and stored at -80°C until the end of the trial. SCTU will request collection of these blood samples at the end of the trial using cryogenic transportation to the University of Leicester.

9.3. CONFIRM TRANSLATIONAL RESEARCH

The goals of the translational research will be to determine the correlation between overall survival and i) PD-L1 expression, ii) mutational burden (estimated by genome-wide analysis of copy number alterations), iii) immunotranscriptomic profile

9.4. FUTURE ETHICALLY APPROVED TRANSLATIONAL RESEARCH

The custodian of the samples will be the Trial Management Group (TMG). Proposals for translational research projects involving the material will be considered by the TMG and/or external ethics committee for approval. Full ethical approval by an NHS research ethics committee must also have been given before the TMG will authorise release of the samples from the trial Tissue Bank for any translational trial.

10 REGULATORY

10.1. CLINICAL TRIAL AUTHORISATION

This trial has a Clinical Trial Authorisation from the UK Competent Authority the Medicines and Healthcare products Regulatory Agency (MHRA).

11 ETHICAL CONSIDERATIONS

The study will be conducted in accordance with the recommendations for physicians involved in research on human patients adopted by the 18th World Medical Assembly, Helsinki 1964 as revised and recognised by governing laws and EU Directives. Each participant's consent to participate in the study should be obtained after a full explanation has been given of treatment options, including the conventional and generally accepted methods of treatment. The right of the participant to refuse to participate in the study without giving reasons must be respected.

After the participant has entered the study, the clinician may give alternative treatment to that specified in the protocol, at any stage, if they feel it to be in the best interest of the participant. However, reasons for doing so should be recorded and the participant will remain within the study for the purpose of follow-up and data analysis according to the treatment option to which they have been allocated. Similarly, the participant remains free to withdraw at any time from protocol treatment and study follow-up without giving reasons and without prejudicing their further treatment.

11.1. ETHICAL APPROVAL

The study protocol has received the favourable opinion of a Research Ethics Committee or Institutional Review Board (IRB) in the approved national participating countries.

11.2. INFORMED CONSENT PROCESS

Informed consent is a process that is initiated prior to an individual agreeing to participate in a trial and continues throughout the individual's participation. In obtaining and documenting informed consent, the investigator should comply with applicable regulatory requirements and should adhere to the principles of GCP.

Discussion of objectives, risks and inconveniences of the trial and the conditions under which it is to be conducted are to be provided to the participant by appropriately delegated staff with knowledge in obtaining informed consent with reference to the patient information leaflet. This information will emphasise that participation in the trial is voluntary and that the participant may withdraw from the trial at any time and for any reason. The participant will be given the opportunity to ask any questions that may arise and provided the opportunity to discuss the trial with family members, friend or an independent healthcare professional outside of the research team and time to consider the information prior to agreeing to participate.

11.3. CONFIDENTIALITY

SCTU will preserve the confidentiality of participants taking part in the trial. The investigator must ensure that participant's anonymity will be maintained and that their identities are protected from unauthorised parties. On CRFs participants will not be identified by their names, but by an identification code.

12 SPONSOR

SCTU, Chief Investigator and other appropriate organisations have been delegated specific duties by the Sponsor and this is documented in the trial task allocation matrix. These include but are not limited to: management of Serious Adverse Events/Reactions and onward reporting of SUSARs; management of deviations and onward reporting of potential Serious Breaches.

The duties assigned to the trial sites (NHS Trusts or others taking part in this study) are detailed in the Non-Commercial Agreement.

12.1. INDEMNITY

The University of Southampton's public and professional indemnity insurance policy provides an indemnity to UoS employees for their potential liability for harm to participants during the conduct of the research. This does not in any way affect an NHS Trust's responsibility for any clinical negligence on the part of its staff.

12.2. FUNDING

Funding is provided by Cancer Research UK through a research grant from the Clinical Research Committee, as well as core funding to SCTU. Additional support is provided by BMS.

12.2.1 *Site payments*

The payments assigned to the trial sites (NHS Trusts or others taking part in this study) are detailed in the Non-Commercial Agreement. This study is adopted onto the NIHR portfolio (CPMS 32290). This enables Trusts to apply to their Local Clinical Research Network for service support costs, if required.

12.2.2 *Participant payments*

Participants will not be paid for participation in this study.

12.3. AUDITS AND INSPECTIONS

The study may be subject to inspection and audit by University of Southampton (under their remit as Sponsor), SCTU (as the Sponsor's delegate) and other regulatory bodies to ensure adherence to the

principles of GCP, Research Governance Framework for Health and Social Care, applicable contracts/agreements and national regulations.

13 TRIAL OVERSIGHT GROUPS

The day-to-day management of the trial will be co-ordinated through the SCTU and oversight will be maintained by the Trial Management Group, the Trial Steering Committee and the Data Monitoring and Ethics Committee.

13.1. TRIAL MANAGEMENT GROUP (TMG)

The TMG is responsible for overseeing progress of the study, including both the clinical and practical aspects. The Chair of the TMG will be the Chief Investigator of the study.

The CONFIRM TMG charter defines the membership, terms of reference, roles, responsibilities, authority, decision-making and relationships of the TMG, including the timing of meetings, frequency and format of meetings and relationships with other trial committees.

13.2. TRIAL STEERING COMMITTEE (TSC)

The TSC act as the oversight body on behalf of the Sponsor and Funder. The TSC will meet in person at least yearly and have at least one further teleconference meeting during the year. The majority of members of the TSC, including the Chair, should be independent of the trial.

The SCTU TSC charter defines the membership, terms of reference, roles, responsibilities, authority, decision-making and relationships of the TSC, including the timing of meetings, frequency and format of meetings and relationships with other trial committees.

13.3. INDEPENDENT DATA MONITORING COMMITTEE (IDMC)

The aim of the IDMC is to safeguard the interests of trial participants, monitor the main outcome measures including safety and efficacy, and monitor the overall conduct of the study.

The CONFIRM IDMC charter defines the membership, terms of reference, roles, responsibilities, authority, decision-making and relationships of the IDMC, including the timing of meetings, methods of providing information to and from the IDMC, frequency and format of meetings, statistical issues and relationships with other trial committees.

14 DATA MANAGEMENT

Participant data will be entered remotely at site and retained in accordance with the current Data Protection Regulations. The PI is responsible for ensuring the accuracy, completeness, and timeliness of the data entered.

The participant data is pseudo anonymised by assigning each participant a participant identifier code which is used to identify the participant during the study and for any participant- specific clarification between SCTU and site. The site retains a participant identification code list which is only available to site staff.

The Participant Information Sheet and Informed Consent Form will specify the participant data to be collected and how it will be managed or might be shared; including handling of all Patient Identifiable Data (PID) and sensitive PID adhering to relevant data protection law.

Trained personnel with specific roles assigned will be granted access to the electronic case report forms (eCRF). eCRF completion guidelines will be provided to the investigator sites to aid data entry of participant information.

Site staff should endeavour to enter trial data within 10 business days of the scheduled visit. Only the Investigator and personnel authorised by them should enter or change data in the eCRFs. When requested, laboratory data must be transcribed, with all investigator observations entered into the eCRF. The original laboratory reports must be retained by the Investigator for future reference.

A Data Management Plan (DMP) providing full details of the study specific data management strategy for the trial will be available and a Trial Schedule with planned and actual milestones, eCRF tracking and central monitoring for active trial management created.

Data queries will either be automatically generated within the eCRF, or manually raised by the study team, if required. All alterations made to the eCRF will be visible via an audit trail which provides the identity of the person who made the change, plus the date and time.

At the end of the trial after all queries have been resolved and the database frozen, the PI will confirm the data integrity by electronically signing all the eCRFs. The eCRFs will be archived according to SCTU policy and a PDF copy including all clinical and meta data returned to the PI for each participant.

14.1. DATA SHARING REQUESTS FOR RESULTS THAT ARE AVAILABLE IN THE PUBLIC DOMAIN

In order to meet our ethical obligation to responsibly share data generated by interventional clinical trials, SCTU operate a transparent data sharing request process. As a minimum, anonymous data will be available for request from three months after publication of an article, to researchers who provide a completed Data Sharing request form that describes a methodologically sound proposal, for the purpose of the approved proposal and if appropriate a signed Data Sharing Agreement. Data will be shared once all parties have signed relevant data sharing documentation.

Researchers interested in our data are asked to complete the Request for Data Sharing form (CTU/FORM/5219) [template located on the SCTU website, www.southampton.ac.uk/ctu] to provide a brief research proposal on how they wish to use the data. It will include; the objectives, what data are requested, timelines for use, intellectual property and publication rights, data release definition in the contract and participant informed consent etc. If considered necessary, a Data Sharing Agreement from Sponsor may be required.

Data may be requested from the Data Sharing Committee at SCTU. Any request will be considered on a monthly basis.

15 MONITORING

15.1. CENTRAL MONITORING

Data stored at SCTU will be checked for missing or unusual values (range checks) and checked for consistency within participants over time. Any suspect data will be returned to the site in the form of data queries. Data queries will be produced at SCTU in the trial database. Sites will respond to the queries providing an explanation/resolution to the discrepancies. There are a number of monitoring features in place at SCTU to ensure reliability and validity of the trial data, which are detailed in the trial monitoring plan (TMP).

Consent forms received by SCTU staff will be checked regularly following the TMP and relevant SCTU SOPs.

15.2. CLINICAL SITE MONITORING

Clinical site monitoring frequency will be determined by the recruitment figures at each participating centre as detailed in the TMP. Triggered site monitoring will occur where required.

15.2.1 *Source Data Verification*

On receipt of a written request from SCTU, the PI will allow the SCTU direct access to relevant source documentation for verification of data entered onto the eCRF (taking into account data protection regulations). Access should also be given to study staff and departments (e.g. pharmacy).

The participants' medical records and other relevant data may also be reviewed by appropriate qualified personnel independent from the SCTU appointed to audit the study, including representatives of the Competent Authority. Details will remain confidential and participants' names will not be recorded outside the study site.

15.3. SOURCE DATA

Source documents are where data are first recorded, and from which participants' CRF data are obtained. These include, but are not limited to, hospital records (from which medical history and previous and concurrent medication may be summarised), clinical and office charts, laboratory and pharmacy records, diaries, microfiches, radiographs, and correspondence.

16 RECORD RETENTION AND ARCHIVING

Trial documents will be retained in a secure location during and after the trial has finished.

The PI or delegate must maintain adequate and accurate records to enable the conduct of the trial to be fully documented and the trial data to be subsequently verified. After trial closure the PI will maintain all source documents and trial related documents. All source documents will be retained for a period of 25 years following the end of the trial.

Sites are responsible for archiving the investigator site file and participants' medical records.

The Sponsor is responsible for archiving the TMF and other relevant trial documentation.

17 PUBLICATION POLICY

Data from all centres will be analysed together and published as soon as possible.

Individual investigators may not publish data concerning their patients that are directly relevant to questions posed by the trial until the TMG has published its report. The TMG will form the basis of the writing committee and advice on the nature of publications. All publications shall include a list of investigators, and if there are named authors, these should include the Chief Investigator, Co-Investigators, Trial Manager, and Statistician(s) involved in the trial. Named authors will be agreed by the CI and Director of SCTU. If there are no named authors then a 'writing committee' will be identified.

18 REFERENCES

1. Fennell, D.A. and R.M. Rudd, *Defective core-apoptosis signalling in diffuse malignant pleural mesothelioma: opportunities for effective drug development.* Lancet Oncol, 2004. **5**(6): p. 354-62.
2. Fennell, D.A., et al., *Advances in the systemic therapy of malignant pleural mesothelioma.* Nat Clin Pract Oncol, 2008. **5**(3): p. 136-47.
3. van Meerbeeck, J.P., et al., *Randomized phase III study of cisplatin with or without raltitrexed in patients with malignant pleural mesothelioma: an intergroup study of the European Organisation for Research and Treatment of Cancer Lung Cancer Group and the National Cancer Institute of Canada.* J Clin Oncol, 2005. **23**(28): p. 6881-9.
4. Vogelzang, N.J., et al., *Phase III study of pemetrexed in combination with cisplatin versus cisplatin alone in patients with malignant pleural mesothelioma.* J Clin Oncol, 2003. **21**(14): p. 2636-44.
5. Zalcman, G., et al., *Bevacizumab for newly diagnosed pleural mesothelioma in the Mesothelioma Avastin Cisplatin Pemetrexed Study (MAPS): a randomised, controlled, open-label, phase 3 trial.* Lancet, 2016. **387**(10026): p. 1405-14.
6. Herbst, R.S., et al., *Pembrolizumab versus docetaxel for previously treated, PD-L1-positive, advanced non-small-cell lung cancer (KEYNOTE-010): a randomised controlled trial.* Lancet, 2016. **387**(10027): p. 1540-50.
7. Langer, C.J., et al., *Carboplatin and pemetrexed with or without pembrolizumab for advanced, non-squamous non-small-cell lung cancer: a randomised, phase 2 cohort of the open-label KEYNOTE-021 study.* Lancet Oncol, 2016.
8. Reck, M., et al., *Pembrolizumab versus Chemotherapy for PD-L1-Positive Non-Small-Cell Lung Cancer.* N Engl J Med, 2016.
9. Mansfield, A.S., et al., *B7-H1 expression in malignant pleural mesothelioma is associated with sarcomatoid histology and poor prognosis.* J Thorac Oncol, 2014. **9**(7): p. 1036-40.
10. Khanna, S., et al., *Malignant Mesothelioma Effusions Are Infiltrated by CD3+ T Cells Highly Expressing PD-L1 and the PD-L1+ Tumor Cells within These Effusions Are Susceptible to ADCC by the Anti-PD-L1 Antibody Avelumab.* J Thorac Oncol, 2016. **11**(11): p. 1993-2005.
11. Tumeh, P.C., et al., *PD-1 blockade induces responses by inhibiting adaptive immune resistance.* Nature, 2014. **515**(7528): p. 568-71.
12. Alley, E.W., et al., *Clinical safety and activity of pembrolizumab in patients with malignant pleural mesothelioma (KEYNOTE-028): preliminary results from a non-randomised, open-label, phase 1b trial.* The Lancet Oncology, 2017. **18**(5): p. 623-630.
13. Hassan, R., et al., *Avelumab (MSB0010718C; anti-PD-L1) in patients with advanced unresectable mesothelioma from the JAVELIN solid tumor phase Ib trial: Safety, clinical activity, and PD-L1 expression.* Journal of Clinical Oncology, 2016. **34**(15_suppl): p. 8503-8503.
14. Bickel, A., et al., *Pembrolizumab-associated minimal change disease in a patient with malignant pleural mesothelioma.* BMC Cancer, 2016. **16**: p. 656.
15. Garon, E.B., et al., *Pembrolizumab for the treatment of non-small-cell lung cancer.* N Engl J Med, 2015. **372**(21): p. 2018-28.
16. Brahmer, J., et al., *Nivolumab versus Docetaxel in Advanced Squamous-Cell Non-Small-Cell Lung Cancer.* N Engl J Med, 2015. **373**(2): p. 123-35.
17. Rizvi, N.A., et al., *Cancer immunology. Mutational landscape determines sensitivity to PD-1 blockade in non-small cell lung cancer.* Science, 2015. **348**(6230): p. 124-8.
18. Ivanov, S.V., et al., *Genomic events associated with progression of pleural malignant mesothelioma.* Int J Cancer, 2009. **124**(3): p. 589-99.
19. Calabro, L., et al., *Tremelimumab for patients with chemotherapy-resistant advanced malignant mesothelioma: an open-label, single-arm, phase 2 trial.* Lancet Oncol, 2013. **14**(11): p. 1104-11.
20. Krug, L.M., et al., *Vorinostat in patients with advanced malignant pleural mesothelioma who have progressed on previous chemotherapy (VANTAGE-014): a phase 3, double-blind, randomised, placebo-controlled trial.* The Lancet Oncology, 2015. **16**(4): p. 447-456.

19 SUMMARY OF SIGNIFICANT CHANGES TO THE PROTOCOL

Protocol date and version	Summary of significant changes
V1 27-Oct-2016	Original document
V2 01-Dec-2017	Amylase and lipase added to assessments; 3.2. DOSE RATIONALE OF NIVOLUMAB added; details of highly effective contraception added
V3 08-Feb-2018	Assessment and treatment windows defined; centre removed as stratification factor; screening and baseline visits differentiated; minor clarifications to text
V4 26-Apr-2018	Lipase removed from assessments. AEs to be reported from start of treatment rather than consent. CPMS ID added to page 40.
V5 31-May-2018	Clarified that events fulfilling criteria for Serious Adverse Events (SAE) that occur between consent and initiation of treatment should be reported as SAEs if they are deemed to be related to the screening procedures.
V6 14-Feb-2019	Addition of PFS as co-primary endpoint; inclusion of second line treatment in eligibility criteria; option of running amylase or lipase testing in Serum Chemistry; death due to disease progression of mesothelioma not required to be reported as SAE. Additional exclusion criteria - if patient enrolled on IND 227 they are ineligible for Confirm
V7 11-Jun2020	Option throughout to perform AST or ALT.<br>Addition of ±3 day window for CT scans.<br>Clarification that patients are to be followed up for a minimum of 6 months post treatment discontinuation.<br>Clarified post-treatment follow up visits and assessments.<br>Clarification to contraceptive requirements.<br>Additional guidance for treatment of infusion related reactions included.<br>Clarification of permitted steroid use.<br>Unblinding separated into emergency and non-emergency unblinding.<br>Nivolumab preparation and administration updated in line with updated IB.<br>Addition of temperature excursion reporting.<br>Clarification on SAE reporting requirements for deaths.<br>Safety reporting windows clarified.<br>RSI updated to IB v18.<br>Updates to exceptions for SAE reporting.<br>Clarification on how co-primary endpoints will be analysed.<br>Removal of interim analyses and added that PD-L1 positive subgroup analyses will be carried out as part of the trial's main analysis.<br>Clarification on translational samples.<br>Data Sharing section updated.<br>Addition of Myocarditis AE Management Algorithm.

APPENDIX 1 ECOG PERFORMANCE STATUS

GRADE	PERFORMANCE STATUS
0	Able to carry out all normal activity without restriction.
1	Restricted in physically strenuous activity but ambulatory and able to carry out light work.
2	Ambulatory and capable of all self-care but unable to carry out any work; up and about more 50% of waking hours.
3	Capable of only limited self-care confined to bed or chair more than 50% of waking hours.
4	Completely disabled; cannot carry out any self-care; totally confined to bed and chair.

* Eastern Cooperative Oncology Group, Robert Comis M.D., Group Chair, 1982, (Oken, et al, 1982)

APPENDIX 2 RECIST CRITERIA

Modified RECIST criteria are described in this document:

http://annonc.oxfordjournals.org/content/15/2/257.full.pdf

RECIST Version 1.1, January 2009, can be accessed at this link:

https://project.eortc.org/recist/wp-content/uploads/sites/4/2015/03/RECISTGuidelines.pdf

APPENDIX 3 CTCAE VERSION 4.03

Please go to the following website to access the CTCAE Version 4.03:

https://evs.nci.nih.gov/ftp1/CTCAE/CTCAE_4.03/CTCAE_4.03_2010-06-14_QuickReference_5x7.pdf

APPENDIX 4 SAFETY MANAGEMENT ALGORITHMS

These general guidelines constitute guidance to the Investigator and may be supplemented by discussions with the Chief Investigator and BMS's Medical Monitor.

A general principle is that differential diagnoses should be diligently evaluated according to standard medical practice. Non-inflammatory aetiologies should be considered and appropriately treated.

Corticosteroids are a primary therapy for immuno-oncology drug-related adverse events. The oral equivalent of the recommended IV doses may be considered for ambulatory patients with low-grade toxicity. The lower bioavailability of oral corticosteroids should be taken into account when switching to the equivalent dose of oral corticosteroids.

Consultation with a medical or surgical specialist, especially prior to an invasive diagnostic or therapeutic procedure, is recommended.

GI Adverse Event Management Algorithm

Rule out non-inflammatory causes. If non-inflammatory cause is identified, treat accordingly and continue I-O therapy. Opiates/narcotics may mask symptoms of perforation. Infliximab should not be used in cases of perforation or sepsis.

Grade of Diarrhea/ Colitis (NCI CTCAE v4)	Management	Follow-up
**Grade 1**<br>Diarrhea:< 4 stools/day over baseline;<br>Colitis: asymptomatic	• Continue I-O therapy per protocol<br>• Symptomatic treatment	• Close monitoring for worsening symptoms.<br>• Educate patient to report worsening immediately<br>If worsens:<br>• Treat as Grade 2 or 3/4
**Grade 2**<br>Diarrhea: 4-6 stools per day over baseline; IV fluids indicated <24 hrs; not interfering with ADL<br>Colitis: abdominal pain; blood in stool	• Delay I-O therapy per protocol<br>• Symptomatic treatment	If improves to grade 1:<br>• Resume I-O therapy per protocol<br>If persists > 5-7 days or recur:<br>• 0.5-1.0 mg/kg/day methylprednisolone or oral equivalent<br>• When symptoms improve to grade 1, taper steroids over at least 1 month, consider prophylactic antibiotics for opportunistic infections, and resume I-O therapy per protocol.<br>If worsens or persists > 3-5 days with oral steroids:<br>• Treat as grade 3/4
**Grade 3-4**<br>Diarrhea (G3): ≥7 stools per day over baseline; incontinence; IV fluids ≥24 hrs; interfering with ADL<br>Colitis (G3): severe abdominal pain, medical intervention indicated, peritoneal signs<br>G4: life-threatening, perforation	• Discontinue I-O therapy per protocol<br>• 1.0 to 2.0 mg/kg/day methylprednisolone IV or IV equivalent<br>• Add prophylactic antibiotics for opportunistic infections<br>• Consider lower endoscopy	If improves:<br>• Continue steroids until grade 1, then taper over at least 1 month<br>If persists > 3-5 days, or recurs after improvement:<br>• Add infliximab 5 mg/kg (if no contraindication). Note: Infliximab should not be used in cases of perforation or sepsis

Patients on IV steroids may be switched to an equivalent dose of oral corticosteroids (e.g. prednisone) at start of tapering or earlier, once sustained clinical improvement is observed. Lower bioavailability of oral corticosteroids should be taken into account when switching to the equivalent dose of oral corticosteroids.

Updated 05-Jul-2016

Renal Adverse Event Management Algorithm

Rule out non-inflammatory causes. If non-inflammatory cause, treat accordingly and continue I-O therapy

Grade of Creatinine Elevation (NCI CTCAE v4)	Management	Follow-up
**Grade 1**<br>Creatinine > ULN and > than baseline but ≤ 1.5x baseline	• Continue I-O therapy per protocol<br>• Monitor creatinine weekly	<u>If returns to baseline</u>:<br>•Resume routine creatinine monitoring per protocol<br><u>If worsens:</u><br>•Treat as Grade 2 or 3/4
**Grade 2-3**<br>Creatinine > 1.5x baseline to ≤ 6x ULN	• Delay I-O therapy per protocol<br>• Monitor creatinine every 2-3 days<br>• 0.5 to 1.0 mg/kg/day methylprednisolone IV or oral equivalent<br>• Consider renal biopsy with nephrology consult	<u>If returns to Grade 1:</u><br>•Taper steroids over at least 1 month, consider prophylactic antibiotics for opportunistic infections, and resume I-O therapy and routine creatinine monitoring per protocol<br><br><u>If elevations persist > 7 days or worsen:</u><br>•Treat as Grade 4
**Grade 4**<br>Creatinine > 6x ULN	• Discontinue I-O therapy per protocol<br>• Monitor creatinine daily<br>• 1.0-2.0 mg/kg/day methylprednisolone IV or IV equivalent<br>• Consult nephrologist<br>• Consider renal biopsy	<u>If returns to Grade 1</u>: Taper steroids over at least 1 month and add prophylactic antibiotics for opportunistic infections

Patients on IV steroids may be switched to an equivalent dose of oral corticosteroids (e.g. prednisone) at start of tapering or earlier, once sustained clinical improvement is observed. Lower bioavailability of oral corticosteroids should be taken into account when switching to the equivalent dose of oral corticosteroids.

Updated 05-Jul-2016

Pulmonary Adverse Event Management Algorithm

Rule out non-inflammatory causes. If non-inflammatory cause, treat accordingly and continue I-O therapy. Evaluate with imaging and pulmonary consultation.

Grade of Pneumonitis (NCI CTCAE v4)	Management	Follow-up
**Grade 1**<br>Radiographic changes only	• Consider delay of I-O therapy<br>• Monitor for symptoms every 2-3 days<br>• Consider Pulmonary and ID consults	• Re-image at least every 3 weeks<br><u>If worsens:</u><br>• Treat as Grade 2 or 3-4
**Grade 2**<br>Mild to moderate new symptoms	• Delay I-O therapy per protocol<br>• Pulmonary and ID consults<br>• Monitor symptoms daily, consider hospitalization<br>• 1.0 mg/kg/day methyl-prednisolone IV or oral equivalent<br>• Consider bronchoscopy , lung biopsy	• Re-image every 1-3 days<br><u>If improves</u>:<br>• When symptoms return to near baseline, taper steroids over at least 1 month and then resume I-O therapy per protocol and consider prophylactic antibiotics<br><u>If not improving after 2 weeks or worsening:</u><br>• Treat as Grade 3-4
**Grade 3-4**<br>Severe new symptoms; New/worsening hypoxia; Life-threatening	• Discontinue I-O therapy per protocol<br>• Hospitalize<br>• Pulmonary and ID consults<br>• 2-4 mg/kg/day methylprednisolone IV or IV equivalent<br>• Add prophylactic antibiotics for opportunistic infections<br>• Consider bronchoscopy, lung biopsy	<u>If improves to baseline:</u><br>• Taper steroids over at least 6 weeks<br><u>If not improving after 48 hours or worsening:</u><br>• Add additional immunosuppression

Patients on IV steroids may be switched to an equivalent dose of oral corticosteroids (e.g. prednisone) at start of tapering or earlier, once sustained clinical improvement is observed. Lower bioavailability of oral corticosteroids should be taken into account when switching to the equivalent dose of oral corticosteroids.

Updated 05-Jul-2016

Hepatic Adverse Event Management Algorithm

Rule out non-inflammatory causes. If non-inflammatory cause, treat accordingly and continue I-O therapy. Consider imaging for obstruction.

Grade of Liver Test Elevation (NCI CTCAE v4)	Management	Follow-up
**Grade 1**<br>AST or ALT > ULN to 3.0 x ULN <u>and/or</u> T. bili > ULN to 1.5 x ULN	• Continue I-O therapy per protocol	• Continue LFT monitoring per protocol<br><u>If worsens:</u><br>• Treat as Grade 2 or 3-4
**Grade 2**<br>AST or ALT > 3.0 to ≤ 5 x ULN <u>and/or</u> T. bili > 1.5 to ≤ 3 x ULN	• Delay I-O therapy per protocol<br>• Increase frequency of monitoring to every 3 days	<u>If returns to baseline</u>:<br>•Resume routine monitoring, resume I-O therapy per protocol<br><br><u>If elevations persist > 5-7 days or worsen :</u><br>• 0.5-1 mg/kg/day methylprednisolone or oral equivalent and when LFT returns to grade 1 or baseline, taper steroids over at least 1 month, consider prophylactic antibiotics for opportunistic infections, and resume I-O therapy per protocol
**Grade 3-4**<br>AST or ALT > 5 x ULN <u>or</u> T.bili >3 x ULN	• Discontinue I-O therapy*<br>• Increase frequency of monitoring to every 1-2 days<br>• 1.0 to 2.0 mg/kg/day methylprednisolone IV or IV equivalent**<br>• Add prophylactic antibiotics for opportunistic infections<br>• Consult gastroenterologist	<u>If returns to grade 2</u>:<br>• Taper steroids over at least 1 month<br><br><u>If does not improve in >3-5 days, worsens or rebounds:</u><br>• Add mycophenolate mofetil 1 g BID<br>• If no response within an additional 3-5 days, consider other immunosuppressants per local guidelines

Patients on IV steroids may be switched to an equivalent dose of oral corticosteroids (e.g. prednisone) at start of tapering or earlier, once sustained clinical improvement is observed. Lower bioavailability of oral corticosteroids should be taken into account when switching to the equivalent dose of oral corticosteroids.

***I-O therapy may be delayed rather than discontinued if AST/ALT ≤ 8 x ULN or T.bili ≤ 5 x ULN.**

**The recommended starting dose for grade 4 hepatitis is 2 mg/kg/day methylprednisolone IV.

Updated 05-Jul-2016

Endocrinopathy Management Algorithm

Rule out non-inflammatory causes. If non-inflammatory cause, treat accordingly and continue I-O therapy. Consider visual field testing, endocrinology consultation, and imaging.

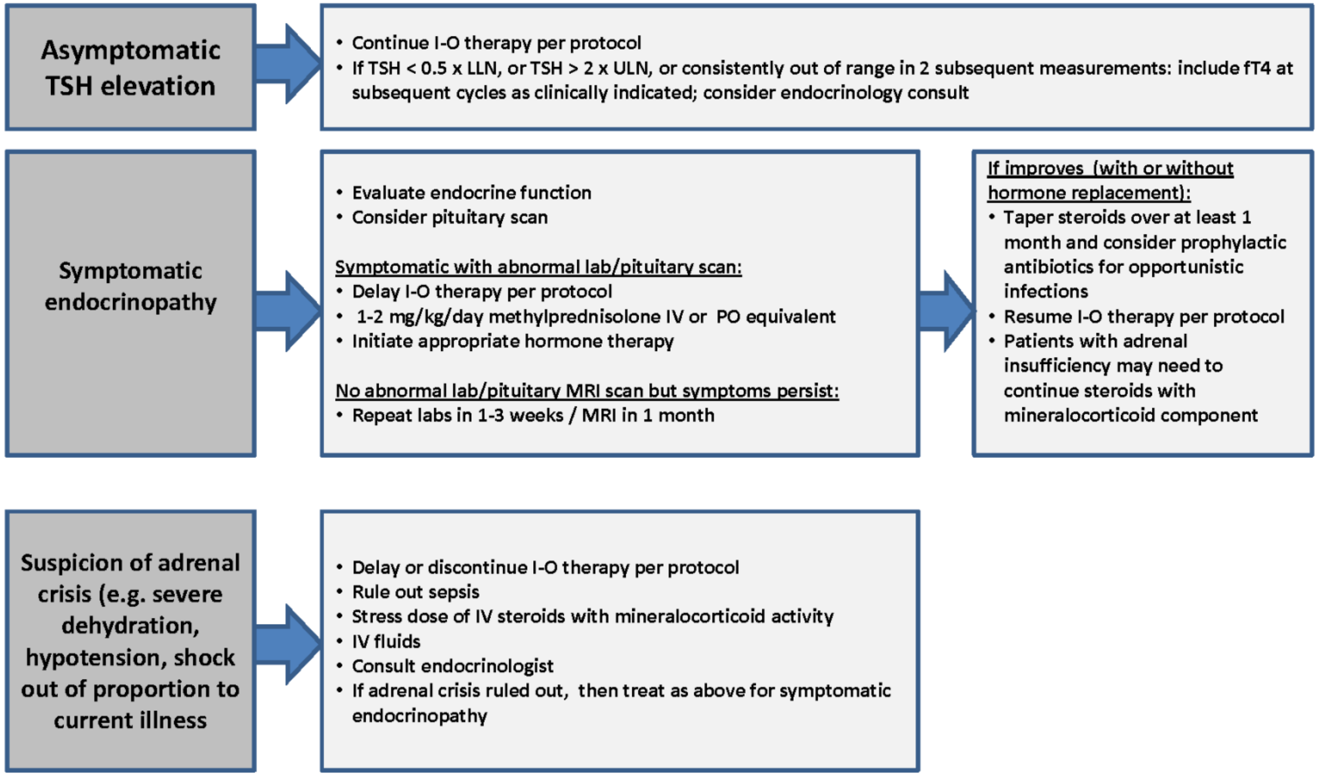

Patients on IV steroids may be switched to an equivalent dose of oral corticosteroids (e.g. prednisone) at start of tapering or earlier, once sustained clinical improvement is observed. Lower bioavailability of oral corticosteroids should be taken into account when switching to the equivalent dose of oral corticosteroids.

Updated 05-Jul-2016

Skin Adverse Event Management Algorithm

Rule out non-inflammatory causes. If non-inflammatory cause, treat accordingly and continue I-O therapy.

Grade of Rash (NCI CTCAE v4)	Management	Follow-up
**Grade 1-2**<br>Covering ≤ 30% BSA*	• Symptomatic therapy (e.g. antihistamines, topical steroids)<br>• Continue I-O therapy per protocol	<u>If persists > 1-2 weeks or recurs:</u><br>• Consider skin biopsy<br>• Delay I-O therapy per protocol<br>• Consider 0.5-1.0 mg/kg/day methylprednisolone IV or oral equivalent. Once improving , taper steroids over at least 1 month, consider prophylactic antibiotics for opportunistic infections, and resume I-O therapy per protocol<br><u>If worsens:</u><br>• Treat as Grade 3-4
**Grade 3-4**<br>Covering >30% BSA; Life threatening consequences*^	• Delay or discontinue I-O therapy per protocol<br>• Consider skin biopsy<br>• Dermatology consult<br>• 1.0-2.0 mg/kg/day IV methylprednisolone IV or IV equivalent	<u>If improves to Grade 1:</u><br>• Taper steroids over at least 1 month and add prophylactic antibiotics for opportunistic infections<br>• Resume I-O therapy per protocol

Patients on IV steroids may be switched to an equivalent dose of oral corticosteroids (e.g. prednisone) at start of tapering or earlier, once sustained clinical improvement is observed. Lower bioavailability of oral corticosteroids should be taken into account when switching to the equivalent dose of oral corticosteroids.

*Refer to NCI CTCAE v4 for term-specific grading criteria.

^If SJS/TEN is suspected, withhold I-O therapy and refer patient for specialized care for assessment and treatment. If SJS or TEN is diagnosed, permanently discontinue I-O therapy.

Updated 05-Jul-2016

Neurological Adverse Event Management Algorithm

Rule out non-inflammatory causes. If non-inflammatory cause, treat accordingly and continue I-O therapy.

Grade of Neurological Toxicity (NCI CTCAE v4)	Management	Follow-up
**Grade 1**<br>Asymptomatic or mild symptoms; Intervention not indicated	• Continue I-O therapy per protocol	Continue to monitor the patient.<br><u>If worsens:</u><br>• Treat as Grade 2 or 3-4
**Grade 2**<br>Moderate symptoms; Limiting instrumental ADL	• Delay I-O therapy per protocol<br>• Treat symptoms per local guidelines<br>• Consider 0.5 to 1.0 mg/kg/day methylprednisolone IV or PO equivalent	<u>If improves to baseline</u>:<br>•Resume I-O therapy per protocol when improved to baseline<br><u>If worsens</u>:<br>• Treat as Grade 3-4
**Grade 3-4**<br>Severe symptoms; Limiting self-care ADL; Life-threatening	• Discontinue I-O therapy per protocol<br>• Obtain neurology consult<br>• Treat symptoms per local guidelines<br>• 1.0-2.0 mg/kg/day IV methylprednisolone IV or IV equivalent<br>• Add prophylactic antibiotics for opportunistic infections	<u>If improves to Grade 2</u>:<br>• Taper steroids over at least 1 month<br><br><u>If worsens or atypical presentation:</u><br>• Consider IVIG or other immunosuppressive therapies per local guidelines

Patients on IV steroids may be switched to an equivalent dose of oral corticosteroids (e.g. prednisone) at start of tapering or earlier, once sustained clinical improvement is observed. Lower bioavailability of oral corticosteroids should be taken into account when switching to the equivalent dose of oral corticosteroids.

Updated 05-Jul-2016

Myocarditis Adverse Event Management Algorithm

Grade of Myocarditis (NCI CTCAE v4)	Management	Follow-up
**Grade 2**<br><br>Symptoms with mild to moderate activity or exertion	• Delay I-O therapy; hospitalization with cardiac monitoring<br>• Urgent cardiology consultation for evaluation and management<br>o Troponin and BNP<br>o ECG ± continuous cardiac monitoring<br>o Echocardiogram<br>o Cardiac MRI<br>• Prompt initiation of 2 mg/kg/day methylprednisolone IV or equivalent	• If worsens, intensify treatment according to grade<br>• Upon recovery, taper steroids over at least 1 month with close monitoring of troponin and BNP as well as for new symptoms<br>• Repeat cardiac MRI for post treatment assessment and cardiology follow-up<br>• Retreatment may be considered after recovery and completion of steroid taper
**Grade 3:** Severe with symptoms at rest or with minimal activity or exertion; intervention indicated<br><br>**Grade 4:** Life-threatening consequences; urgent intervention indicated (e.g., continuous IV therapy or mechanical hemodynamic support)	• Permanently discontinue I-O therapy<br>• Hospitalize to intensive cardiac monitoring<br>• Cardiac evaluation to include:<br>– Troponin and BNP monitoring<br>– ECG ± continuous cardiac monitoring<br>– Echocardiogram<br>– Cardiac MRI<br>– Myocardial biopsy if feasible<br>• Immediate initiation of 2 mg/kg/day methylprednisolone IV or 1 g IV bolus<br>• Consider adding a second immunosuppressive agent<br>Additionally, for Grade 4:<br>• Hospitalize/transfer to institution with expertise in intensive cardiac monitoring<br>• Consider ATG as second agent given its immediate effect	• If no improvement, consider additional immunosuppression<br>• Upon recovery, taper steroids over at least 1 month with close monitoring of troponin and BNP as well as for new symptoms<br>• Repeat cardiac MRI for post treatment assessments and cardiology follow-up

Patients on IV steroids may be switched to an equivalent dose of oral corticosteroids (eg, prednisone) at start of tapering or earlier, once sustained clinical improvement is observed. Lower bioavailability of oral corticosteroids should be taken into account when switching to the equivalent dose of oral corticosteroids.
Prophylactic antibiotics should be considered in the setting of ongoing immunosuppression.
ATG = anti-thymocyte globulin; BNP = B-type natriuretic peptide; ECG = electrocardiogram; IV = intravenous; MRI = magnetic resonance imaging
